# Supplementary material for: Innovative approach to improve information accuracy in a two-district cross-sectional study in Bihar, India
Source: BMJ Open. 2022 Jan 6;12(1):e051427. doi: 10.1136/bmjopen-2021-051427 (PMC8739057; doi:10.1136/bmjopen-2021-051427)
Supplement: Supplementary data [file bmjopen-2021-051427supp001.pdf]

Supplementary Information 1

Statistical Annealing Theory

To construct a standard error (SE) for the HMIS coverage estimator, we use the average mean square error (MSE) between the HMIS and LQAS coverage estimators over all the blocks composing one district. The MSE is calculated for the whole district, and can be decomposed in two ways:

$$MSE = \frac{1}{K}\sum_{k=1}^{K}(p_{HMIS\_k} - p_{LQAS\_k})^2 \quad \text{(first decomposition)}$$
$$= \sigma_{HMIS}^2 + \sigma_{LQAS}^2 \quad \text{(second decomposition)}$$

where:

* K is the total number of blocks in the district;
* k is the index for the block in the concerned district, and ranges from 1 to K;
* σ_{HMIS} is the SE of the HMIS coverage p_{HMIS};
* σ_{LQAS} is the SE of the LQAS coverage p_{LQAS}.

The first decomposition measures how much the HMIS and LQAS coverage estimators differ across the blocks composing the district. The second decomposition shows the MSE is also the sum of the two variances of HMIS and LQAS estimators, assuming each source of data was collected independently from each other. This decomposition also assumes that both HMIS and LQAS are unbiased, i.e. that the expectation of both estimates is the same). We calculate the MSE using the first decomposition and subtract the LQAS variance to obtain the HMIS variance (and thus σ_{HMIS}).

Combined coverage estimator, SE and confidence interval

We define the combined coverage estimator and its SE as a weighted average of the HMIS and LQAS estimators:

$$p_{combined} = w * p_{HMIS} + (1 - w) * p_{LQAS}$$
$$\sigma_{combined} = \sqrt{w^2 * \sigma_{HMIS}^2 + (1 - w)^2 * \sigma_{LQAS}^2}$$

Where the weighting factor w takes values between 0 and 1:

$$w = \frac{\sigma_{LQAS}^2}{\sigma_{HMIS}^2 + \sigma_{LQAS}^2} = \frac{\sigma_{LQAS}^2}{MSE - \sigma_{LQAS}^2 + \sigma_{LQAS}^2} = \frac{\sigma_{LQAS}^2}{MSE}$$

The 95% CI for the combined coverage estimator is calculated using the standard formula, $p_{combined} \pm 1.96 \times \sigma_{combined}$.
