# Supplementary material for: Innovative approach to improve information accuracy in a two-district cross-sectional study in Bihar, India
Source: BMJ Open. 2022 Jan 6;12(1):e051427. doi: 10.1136/bmjopen-2021-051427 (PMC8739057; doi:10.1136/bmjopen-2021-051427)
Supplement: Supplementary data [file bmjopen-2021-051427supp002.pdf]

Supplementary Information 2

ANC & Birth preparedness indicators

For the proportion of women who had 3 or more ANC during their pregnancy, the weighting factor ranges from 0.35 to 0.49 in Aurangabad, and from 0.09 to 0.13 in Gopalganj (Table S1). The combined estimate differs from the LQAS estimate by no more than 10%. Standard errors for the HMIS data are 2.8 to 3.4 times larger than those calculated for the combined estimates.

Table S1: Women who had 3 or more ANC visits during their pregnancy.

Block	p_{HMIS}	σ_{HMIS}	p_{lqas}	σ_{lqas}	w	$p_{combined}$	$\sigma_{combined}$	95% CI	$\sigma_{HMIS} / \sigma_{combined}$
Aurangabad Sadar	0.55	0.08	0.55	0.08	0.48	0.55	0.058	(0.44,0.66)	1.44
Barun	0.48	0.09	0.76	0.07	0.35	0.66	0.055	(0.56,0.77)	1.68
Daudnagar	0.71	0.08	0.53	0.08	0.49	0.62	0.058	(0.5,0.73)	1.43
Deo	0.6	0.08	0.58	0.08	0.48	0.59	0.058	(0.48,0.7)	1.45
Goh	0.67	0.09	0.66	0.08	0.44	0.66	0.058	(0.55,0.78)	1.51
Haspura	0.54	0.08	0.61	0.08	0.47	0.58	0.058	(0.46,0.69)	1.46
Kutumba	0.8	0.09	0.66	0.08	0.44	0.72	0.058	(0.61,0.83)	1.51
Madanpur	0.7	0.09	0.66	0.08	0.44	0.67	0.058	(0.56,0.79)	1.51
Nabinagar	0.62	0.08	0.55	0.08	0.48	0.58	0.058	(0.47,0.7)	1.44
Obra	0.73	0.09	0.66	0.08	0.44	0.69	0.058	(0.58,0.8)	1.51
Rafiganj	0.61	0.08	0.61	0.08	0.47	0.61	0.058	(0.49,0.72)	1.46
**Aurangabad District Average**	**0.64**	**0.03**	**0.62**	**0.02**		**0.63**	**0.018**	**(0.6,0.67)**	**1.49**
Baikunthpur	0.72	0.21	0.53	0.08	0.13	0.55	0.076	(0.4,0.7)	2.79
Barauli	0.77	0.21	0.45	0.08	0.13	0.49	0.075	(0.34,0.64)	2.8
Bhorey	0.7	0.21	0.58	0.08	0.13	0.59	0.075	(0.45,0.74)	2.82
Bijaipur	0.8	0.21	0.74	0.07	0.1	0.74	0.068	(0.61,0.88)	3.17
Gopalganj Sadar	0.8	0.21	0.55	0.08	0.13	0.58	0.075	(0.44,0.73)	2.8
Hathua	0.97	0.21	0.47	0.08	0.13	0.54	0.076	(0.39,0.69)	2.79
Kateya	0.8	0.21	0.74	0.07	0.1	0.74	0.068	(0.61,0.88)	3.17
Kuchaikote	0.9	0.21	0.47	0.08	0.13	0.53	0.076	(0.38,0.68)	2.79
Manjha	0.61	0.22	0.79	0.07	0.09	0.77	0.063	(0.65,0.9)	3.42
Pach Deuri	0.5	0.21	0.47	0.08	0.13	0.48	0.076	(0.33,0.62)	2.79
Phulwaria	0.56	0.21	0.58	0.08	0.13	0.58	0.075	(0.43,0.72)	2.82
Sidhwalia	0.72	0.21	0.66	0.08	0.12	0.67	0.072	(0.52,0.81)	2.94
Thawe	0.62	0.22	0.79	0.07	0.09	0.77	0.063	(0.65,0.9)	3.42
Uchkagaon	0.75	0.22	0.76	0.07	0.09	0.76	0.066	(0.63,0.89)	3.28

**Gopalganj District Average**	**0.75**	**0.06**	**0.59**	**0.02**		**0.61**	**0.021**	**(0.57,0.65)**	**2.91**

For the proportion of women who received TT1 during their pregnancy, the weighting factor ranges from 0.002 to 0.009 in Aurangabad, and from 0.006 to 0.038 in Gopalganj (Table S2). The combined estimate differs from the LQAS estimate by no more than 1%. Standard errors for the HMIS data are 5.1 to 20.2 times larger than those calculated for the combined estimates.

Table S2 Women who received TT1 during their pregnancy.

Block	p_{HMIS}	σ_{HMIS}	p_{lqas}	σ_{lqas}	w	$p_{combined}$	$\sigma_{combined}$	95% CI	σ_{HMIS} / $\sigma_{combined}$
Aurangabad Sadar	0.64	0.39	0.95	0.04	0.01	0.94	0.036	(0.87,1)	10.78
Barun	0.61	0.39	0.97	0.03	0	0.97	0.026	(0.92,1)	15.04
Daudnagar	0.61	0.39	1	0.02	0	1	0.019	(0.96,1)	20.19
Deo	0.59	0.39	0.97	0.03	0	0.97	0.026	(0.92,1)	15.04
Goh	0.54	0.39	0.95	0.04	0.01	0.94	0.036	(0.87,1)	10.78
Haspura	0.59	0.39	1	0.02	0	1	0.019	(0.96,1)	20.19
Kutumba	0.62	0.39	1	0.02	0	1	0.019	(0.96,1)	20.19
Madanpur	0.52	0.39	1	0.02	0	1	0.019	(0.96,1)	20.19
Nabinagar	0.87	0.39	0.97	0.03	0	0.97	0.026	(0.92,1)	15.04
Obra	0.54	0.39	1	0.02	0	1	0.019	(0.96,1)	20.19
Rafiganj	0.54	0.39	1	0.02	0	1	0.019	(0.96,1)	20.19
**Aurangabad District Average**	**0.61**	**0.12**	**0.98**	**0.01**		**0.98**	**0.008**	**(0.97,1)**	**15.6**
Baikunthpur	0.65	0.25	1	0.02	0.01	1	0.019	(0.96,1)	13.15
Barauli	0.95	0.25	0.95	0.04	0.02	0.95	0.036	(0.88,1)	7.02
Bhorey	0.88	0.25	1	0.02	0.01	1	0.019	(0.96,1)	13.15
Bijaipur	0.81	0.25	0.95	0.04	0.02	0.94	0.036	(0.87,1)	7.02
Gopalganj Sadar	0.97	0.25	0.95	0.04	0.02	0.95	0.036	(0.88,1)	7.02
Hathua	0.48	0.25	0.97	0.03	0.01	0.97	0.026	(0.92,1)	9.79
Kateya	0.84	0.25	0.89	0.05	0.04	0.89	0.049	(0.8,0.99)	5.11
Kuchaikote	0.93	0.25	1	0.02	0.01	1	0.019	(0.96,1)	13.15
Manjha	0.75	0.25	0.92	0.04	0.03	0.92	0.043	(0.83,1)	5.81
Pach Deuri	0.6	0.25	1	0.02	0.01	1	0.019	(0.96,1)	13.15
Phulwaria	0.63	0.25	1	0.02	0.01	1	0.019	(0.96,1)	13.15
Sidhwalia	0.68	0.25	0.97	0.03	0.01	0.97	0.026	(0.92,1)	9.79
Thawe	0.72	0.25	1	0.02	0.01	1	0.019	(0.96,1)	13.15
Uchkagaon	0.87	0.25	0.95	0.04	0.02	0.95	0.036	(0.88,1)	7.02

**Gopalganj District Average**	**0.78**	**0.07**	**0.97**	**0.01**		**0.97**	**0.008**	**(0.95,0.98)**	**8.82**

For the proportion of women who received TT2 during their pregnancy, the weighting factor ranges from 0.11 to 0.24 in Aurangabad, and from 0.08 to 0.19 in Gopalganj (Table S3). The combined estimate differs from the LQAS estimate by no more than 7%. Standard errors for the HMIS data are 2 to 3.5 times larger than those calculated for the combined estimates.

Table S3 Women who received TT2 during their pregnancy.

Block	p_{HMIS}	σ_{HMIS}	p_{lqas}	σ_{lqas}	w	$p_{combined}$	$\sigma_{combined}$	95% CI	$\sigma\_HMIS / \sigma\_combined$
Aurangabad Sadar	0.55	0.14	0.63	0.08	0.23	0.61	0.069	(0.48,0.75)	2.1
Barun	0.8	0.15	0.71	0.07	0.2	0.73	0.066	(0.6,0.86)	2.23
Daudnagar	0.82	0.14	0.61	0.08	0.23	0.66	0.069	(0.52,0.79)	2.07
Deo	0.78	0.15	0.74	0.07	0.19	0.75	0.064	(0.62,0.87)	2.3
Goh	0.8	0.14	0.58	0.08	0.24	0.63	0.07	(0.5,0.77)	2.05
Haspura	0.8	0.14	0.58	0.08	0.24	0.63	0.07	(0.49,0.77)	2.05
Kutumba	0.82	0.15	0.71	0.07	0.2	0.73	0.066	(0.6,0.86)	2.23
Madanpur	0.84	0.14	0.55	0.08	0.24	0.62	0.07	(0.48,0.76)	2.03
Nabinagar	0.79	0.15	0.87	0.05	0.11	0.86	0.052	(0.76,0.96)	2.99
Obra	0.85	0.15	0.74	0.07	0.19	0.76	0.064	(0.63,0.88)	2.3
Rafiganj	0.85	0.15	0.71	0.07	0.2	0.74	0.066	(0.61,0.87)	2.23
**Aurangabad District Average**	**0.8**	**0.05**	**0.68**	**0.02**		**0.71**	**0.02**	**(0.67,0.75)**	**2.26**
Baikunthpur	0.86	0.17	0.87	0.05	0.1	0.87	0.052	(0.77,0.97)	3.22
Barauli	1.04	0.17	0.89	0.05	0.08	0.91	0.048	(0.81,1)	3.54
Bhorey	0.85	0.17	0.87	0.05	0.1	0.87	0.052	(0.76,0.97)	3.22
Bijaipur	0.9	0.17	0.84	0.06	0.11	0.85	0.056	(0.74,0.96)	2.98
Gopalganj Sadar	0.98	0.16	0.71	0.07	0.17	0.76	0.067	(0.63,0.89)	2.4
Hathua	0.93	0.16	0.79	0.07	0.14	0.81	0.061	(0.69,0.93)	2.67
Kateya	1.03	0.16	0.71	0.07	0.17	0.77	0.067	(0.64,0.9)	2.4
Kuchaikote	0.87	0.17	0.89	0.05	0.08	0.89	0.048	(0.8,0.99)	3.54
Manjha	0.87	0.16	0.76	0.07	0.15	0.78	0.063	(0.65,0.9)	2.56
Pach Deuri	0.4	0.16	0.74	0.07	0.16	0.68	0.065	(0.55,0.81)	2.47
Phulwaria	0.7	0.17	0.87	0.05	0.1	0.85	0.052	(0.75,0.95)	3.22
Sidhwalia	0.74	0.16	0.76	0.07	0.15	0.76	0.063	(0.63,0.88)	2.56
Thawe	0.84	0.16	0.66	0.08	0.19	0.69	0.069	(0.56,0.83)	2.29
Uchkagaon	0.92	0.16	0.76	0.07	0.15	0.79	0.063	(0.66,0.91)	2.56

**Gopalganj District Average**	**0.87**	**0.05**	**0.81**	**0.02**		**0.82**	**0.016**	**(0.79,0.85)**	**2.96**

For the proportion of women who received at least 100 IFA tablets during their pregnancy, the weighting factor ranges from 0.002 to 0.02 in Aurangabad, and from 0.001 to 0.01 in Gopalganj (Table S4). The combined estimate differs from the LQAS estimate by no more than 1%. Standard errors for the HMIS data are 7 to 32.3 times larger than those calculated for the combined estimates.

Table S4 Women who received at least 100 IFA tablets during their pregnancy.

Block	p_{HMIS}	σ_{HMIS}	p_{lqas}	σ_{lqas}	w	$p_{combined}$	$\sigma_{combined}$	95% CI	$\sigma\_HMIS / \sigma\_combined$
Aurangabad Sadar	0.13	0.39	0.05	0.04	0.01	0.05	0.036	(0,0.12)	10.68
Barun	0.46	0.39	0.03	0.03	0	0.03	0.026	(0,0.08)	14.9
Daudnagar	0.59	0.39	0	0.02	0	0	0.019	(0,0.04)	20.01
Deo	0.45	0.39	0	0.02	0	0	0.019	(0,0.04)	20.01
Goh	0.18	0.39	0	0.02	0	0	0.019	(0,0.04)	20.01
Haspura	0.16	0.38	0.08	0.04	0.01	0.08	0.043	(0,0.17)	8.85
Kutumba	0.34	0.39	0.05	0.04	0.01	0.06	0.036	(0,0.13)	10.68
Madanpur	0.42	0.39	0	0.02	0	0	0.019	(0,0.04)	20.01
Nabinagar	0.6	0.38	0.13	0.05	0.02	0.14	0.054	(0.03,0.25)	7.06
Obra	0.34	0.39	0	0.02	0	0	0.019	(0,0.04)	20.01
Rafiganj	0.51	0.39	0	0.02	0	0	0.019	(0,0.04)	20.01
**Aurangabad District Average**	**0.39**	**0.12**	**0.03**	**0.01**		**0.04**	**0.01**	**(0.02,0.05)**	**12.02**
Baikunthpur	0.52	0.62	0.03	0.03	0	0.03	0.026	(0,0.08)	24.08
Barauli	0.4	0.62	0	0.02	0	0	0.019	(0,0.04)	32.33
Bhorey	0.79	0.62	0.11	0.05	0.01	0.11	0.05	(0.01,0.21)	12.56
Bijaipur	0.29	0.62	0.03	0.03	0	0.03	0.026	(0,0.08)	24.08
Gopalganj Sadar	0.63	0.62	0.05	0.04	0	0.05	0.036	(0,0.13)	17.26
Hathua	0.86	0.62	0.03	0.03	0	0.03	0.026	(0,0.08)	24.08
Kateya	0.8	0.62	0.03	0.03	0	0.03	0.026	(0,0.08)	24.08
Kuchaikote	0.6	0.62	0.03	0.03	0	0.03	0.026	(0,0.08)	24.08
Manjha	0.36	0.62	0.05	0.04	0	0.05	0.036	(0,0.12)	17.26
Pach Deuri	1.47	0.62	0.13	0.05	0.01	0.14	0.055	(0.03,0.25)	11.4
Phulwaria	0.4	0.62	0.05	0.04	0	0.05	0.036	(0,0.12)	17.26
Sidhwalia	0.38	0.62	0.05	0.04	0	0.05	0.036	(0,0.12)	17.26
Thawe	0.59	0.62	0.05	0.04	0	0.05	0.036	(0,0.13)	17.26
Uchkagaon	0.43	0.62	0	0.02	0	0	0.019	(0,0.04)	32.33

**Gopalganj District Average**	**0.59**	**0.18**	**0.04**	**0.01**		**0.04**	**0.009**	**(0.03,0.06)**	**20.2**

Birth history indicators

For the proportion of infants who had institutional delivery, the weighting factor ranges from 0.01 to 0.02 in Aurangabad, and from 0.005 to 0.01 in Gopalganj (Table S5). The combined estimate differs from the LQAS estimate by no more than 1%. Standard errors for the HMIS data are 6.7 to 14.6 times larger than those calculated for the combined estimates.

Table S5 Institutional Deliveries (all institutions)

Block	p_{HMIS}	σ_{HMIS}	p_{lqas}	σ_{lqas}	w	$p_{combined}$	$\sigma_{combined}$	95% CI	$\sigma\_HMIS / \sigma\_combined$
Aurangabad Sadar	0.03	0.44	0.88	0.04	0.01	0.87	0.043	(0.78,0.95)	10.11
Barun	0.28	0.44	0.82	0.05	0.01	0.82	0.05	(0.72,0.92)	8.73
Daudnagar	0.41	0.44	0.81	0.05	0.01	0.8	0.052	(0.7,0.9)	8.41
Deo	0.38	0.43	0.63	0.06	0.02	0.63	0.063	(0.5,0.75)	6.88
Goh	0.38	0.43	0.6	0.06	0.02	0.59	0.064	(0.47,0.72)	6.77
Haspura	0.51	0.44	0.77	0.06	0.02	0.77	0.055	(0.66,0.88)	7.91
Kutumba	0.41	0.44	0.86	0.05	0.01	0.85	0.046	(0.77,0.94)	9.56
Madanpur	0.4	0.44	0.7	0.06	0.02	0.7	0.06	(0.58,0.81)	7.26
Nabinagar	0.25	0.43	0.63	0.06	0.02	0.62	0.063	(0.5,0.75)	6.88
Obra	0.3	0.44	0.84	0.05	0.01	0.84	0.048	(0.74,0.93)	9.1
Rafiganj	0.38	0.43	0.58	0.07	0.02	0.57	0.065	(0.45,0.7)	6.72
**Aurangabad District Average**	**0.34**	**0.13**	**0.73**	**0.02**		**0.72**	**0.018**	**(0.69,0.76)**	**7.59**
Baikunthpur	0.44	0.49	0.79	0.05	0.01	0.79	0.054	(0.68,0.89)	9.17
Barauli	0.47	0.49	0.84	0.05	0.01	0.84	0.048	(0.74,0.93)	10.25
Bhorey	0.48	0.49	0.81	0.05	0.01	0.8	0.052	(0.7,0.91)	9.47
Bijaipur	0.35	0.49	0.82	0.05	0.01	0.82	0.05	(0.72,0.92)	9.83
Gopalganj Sadar	0	0.49	0.84	0.05	0.01	0.83	0.048	(0.74,0.93)	10.25
Hathua	0.55	0.49	0.91	0.04	0.01	0.91	0.037	(0.84,0.98)	13.21
Kateya	0.48	0.49	0.93	0.03	0	0.93	0.034	(0.86,0.99)	14.63
Kuchaikote	0.34	0.49	0.77	0.06	0.01	0.77	0.055	(0.66,0.87)	8.91
Manjha	0.2	0.49	0.81	0.05	0.01	0.8	0.052	(0.7,0.9)	9.47
Pach Deuri	0.46	0.49	0.77	0.06	0.01	0.77	0.055	(0.66,0.88)	8.91
Phulwaria	0.42	0.49	0.84	0.05	0.01	0.84	0.048	(0.74,0.93)	10.25
Sidhwalia	0.31	0.49	0.81	0.05	0.01	0.8	0.052	(0.7,0.9)	9.47
Thawe	0.33	0.49	0.93	0.03	0	0.93	0.034	(0.86,0.99)	14.63
Uchkagaon	0.32	0.49	0.91	0.04	0.01	0.91	0.037	(0.84,0.98)	13.21

**Gopalganj District Average**	**0.37**	**0.14**	**0.84**	**0.01**		**0.83**	**0.014**	**(0.8,0.86)**	**9.96**

For the proportion of infants who had institutional delivery in a public facility, the weighting factor ranges from 0.066 to 0.073 in Aurangabad, and from 0.05 to 0.06 in Gopalganj (Table S6). The combined estimate differs from the LQAS estimate by no more than 4%. Standard errors for the HMIS data are 3.7 to 4.4 times larger than those calculated for the combined estimates.

Table S6 Public Institutional Deliveries

Block	p_{HMIS}	σ_{HMIS}	p_{lqas}	σ_{lqas}	w	$p_{combined}$	$\sigma_{combined}$	95% CI	$\sigma\_HMIS / \sigma\_combined$
Aurangabad Sadar	0.03	0.24	0.65	0.06	0.07	0.61	0.061	(0.49,0.73)	3.88
Barun	0.28	0.24	0.56	0.07	0.07	0.54	0.063	(0.42,0.67)	3.73
Daudnagar	0.41	0.24	0.51	0.07	0.07	0.5	0.064	(0.38,0.63)	3.7
Deo	0.38	0.24	0.44	0.07	0.07	0.43	0.063	(0.31,0.56)	3.73
Goh	0.38	0.24	0.44	0.07	0.07	0.43	0.063	(0.31,0.56)	3.73
Haspura	0.51	0.24	0.61	0.06	0.07	0.61	0.062	(0.48,0.73)	3.8
Kutumba	0.41	0.24	0.61	0.06	0.07	0.6	0.062	(0.48,0.72)	3.8
Madanpur	0.4	0.24	0.58	0.07	0.07	0.57	0.063	(0.44,0.69)	3.75
Nabinagar	0.25	0.24	0.4	0.06	0.07	0.39	0.063	(0.27,0.52)	3.77
Obra	0.3	0.24	0.56	0.07	0.07	0.54	0.063	(0.42,0.67)	3.73
Rafiganj	0.38	0.24	0.44	0.07	0.07	0.43	0.063	(0.31,0.56)	3.73
**Aurangabad District Average**	**0.34**	**0.07**	**0.52**	**0.02**		**0.51**	**0.019**	**(0.47,0.54)**	**3.76**
Baikunthpur	0.44	0.25	0.58	0.07	0.06	0.57	0.063	(0.45,0.69)	4
Barauli	0.47	0.25	0.61	0.06	0.06	0.61	0.062	(0.48,0.73)	4.06
Bhorey	0.48	0.25	0.56	0.07	0.06	0.56	0.064	(0.43,0.68)	3.98
Bijaipur	0.35	0.25	0.65	0.06	0.06	0.63	0.061	(0.51,0.75)	4.14
Gopalganj Sadar	0	0.25	0.56	0.07	0.06	0.53	0.064	(0.4,0.65)	3.98
Hathua	0.55	0.25	0.65	0.06	0.06	0.64	0.061	(0.52,0.76)	4.14
Kateya	0.48	0.25	0.7	0.06	0.05	0.69	0.059	(0.57,0.81)	4.32
Kuchaikote	0.34	0.25	0.58	0.07	0.06	0.56	0.063	(0.44,0.69)	4
Manjha	0.2	0.25	0.53	0.07	0.06	0.51	0.064	(0.38,0.63)	3.96
Pach Deuri	0.46	0.25	0.6	0.06	0.06	0.59	0.063	(0.46,0.71)	4.03
Phulwaria	0.42	0.25	0.49	0.07	0.06	0.49	0.064	(0.36,0.61)	3.95
Sidhwalia	0.31	0.25	0.72	0.06	0.05	0.7	0.058	(0.58,0.81)	4.4
Thawe	0.33	0.25	0.47	0.07	0.06	0.46	0.064	(0.34,0.59)	3.96
Uchkagaon	0.32	0.25	0.58	0.07	0.06	0.56	0.063	(0.44,0.69)	4

**Gopalganj District Average**	**0.37**	**0.07**	**0.59**	**0.02**		**0.58**	**0.018**	**(0.54,0.61)**	**4.04**

FLW support and Neonatal Health indicators

For the proportion of infants who were weighed during delivery, the weighting factor ranges from 0.04 to 0.08 in Aurangabad, and from 0.03 to 0.05 in Gopalganj (Table S7). The combined estimate differs from the LQAS estimate by no more than 3%. Standard errors for the HMIS data are 3.5 to 6.2 times larger than those calculated for the combined estimates.

Table S7 Infants who were weighed during delivery.

Block	p_{HMIS}	σ_{HMIS}	p_{lqas}	σ_{lqas}	w	$p_{combined}$	$\sigma_{combined}$	95% CI	$\sigma\_HMIS / \sigma\_combined$
Aurangabad Sadar	0.1	0.27	0.84	0.06	0.04	0.81	0.058	(0.7,0.92)	4.73
Barun	0.49	0.27	0.76	0.07	0.06	0.75	0.067	(0.62,0.88)	4.06
Daudnagar	0.52	0.27	0.63	0.08	0.08	0.62	0.075	(0.48,0.77)	3.58
Deo	0.46	0.27	0.58	0.08	0.08	0.57	0.077	(0.42,0.72)	3.5
Goh	0.52	0.27	0.55	0.08	0.08	0.55	0.077	(0.4,0.7)	3.47
Haspura	0.61	0.27	0.63	0.08	0.08	0.63	0.075	(0.48,0.78)	3.58
Kutumba	0.53	0.27	0.71	0.07	0.07	0.7	0.071	(0.56,0.84)	3.8
Madanpur	0.57	0.27	0.71	0.07	0.07	0.7	0.071	(0.56,0.84)	3.8
Nabinagar	0.66	0.27	0.66	0.08	0.08	0.66	0.074	(0.51,0.8)	3.64
Obra	0.46	0.27	0.82	0.06	0.05	0.8	0.061	(0.68,0.92)	4.45
Rafiganj	0.6	0.27	0.42	0.08	0.08	0.44	0.077	(0.29,0.59)	3.5
**Aurangabad District Average**	**0.51**	**0.08**	**0.66**	**0.02**		**0.65**	**0.022**	**(0.61,0.69)**	**3.75**
Baikunthpur	0.58	0.34	0.76	0.07	0.04	0.76	0.068	(0.62,0.89)	4.96
Barauli	0.52	0.33	0.61	0.08	0.05	0.6	0.077	(0.45,0.75)	4.31
Bhorey	0.65	0.33	0.63	0.08	0.05	0.63	0.076	(0.48,0.78)	4.37
Bijaipur	0.48	0.33	0.74	0.07	0.04	0.73	0.07	(0.59,0.86)	4.79
Gopalganj Sadar	0.05	0.33	0.74	0.07	0.04	0.71	0.07	(0.57,0.84)	4.79
Hathua	0.54	0.34	0.87	0.05	0.03	0.86	0.054	(0.75,0.97)	6.24
Kateya	0.6	0.34	0.82	0.06	0.03	0.81	0.062	(0.69,0.93)	5.44
Kuchaikote	0.4	0.34	0.79	0.07	0.04	0.77	0.065	(0.65,0.9)	5.17
Manjha	0.29	0.33	0.68	0.08	0.05	0.67	0.074	(0.52,0.81)	4.54
Pach Deuri	0.56	0.33	0.68	0.08	0.05	0.68	0.074	(0.53,0.82)	4.54
Phulwaria	0.5	0.33	0.66	0.08	0.05	0.65	0.075	(0.5,0.8)	4.45
Sidhwalia	0.37	0.34	0.79	0.07	0.04	0.77	0.065	(0.65,0.9)	5.17
Thawe	0.35	0.34	0.82	0.06	0.03	0.8	0.062	(0.68,0.92)	5.44
Uchkagaon	0.44	0.34	0.84	0.06	0.03	0.83	0.058	(0.72,0.94)	5.78

**Gopalganj District Average**	**0.45**	**0.09**	**0.74**	**0.02**		**0.73**	**0.019**	**(0.7,0.77)**	**4.94**

For the proportion of infants who were breast fed within an hour of their birth, the weighting factor ranges from 0.15 to 0.20 in Aurangabad, and from 0.07 to 0.13 in Gopalganj (Table S8). The combined estimate differs from the LQAS estimate by no more than 8%. Standard errors for the HMIS data are 2.2 to 3.8 times larger than those calculated for the combined estimates.

Table S8 Infants who were breast fed within an hour of their birth.

Block	p_{HMIS}	σ_{HMIS}	p_{lqas}	σ_{lqas}	w	$p_{combined}$	$\sigma_{combined}$	95% CI	$\sigma\_HMIS / \sigma\_combined$
Aurangabad Sadar	0.1	0.16	0.5	0.08	0.2	0.42	0.072	(0.28,0.56)	2.22
Barun	0.49	0.17	0.76	0.07	0.15	0.72	0.064	(0.6,0.85)	2.61
Daudnagar	0.52	0.16	0.42	0.08	0.2	0.44	0.072	(0.3,0.58)	2.24
Deo	0.46	0.16	0.5	0.08	0.2	0.49	0.072	(0.35,0.63)	2.22
Goh	0.52	0.16	0.68	0.08	0.18	0.66	0.068	(0.52,0.79)	2.38
Haspura	0.61	0.16	0.61	0.08	0.19	0.61	0.071	(0.47,0.74)	2.27
Kutumba	0.53	0.16	0.63	0.08	0.19	0.61	0.07	(0.47,0.75)	2.3
Madanpur	0.57	0.16	0.37	0.08	0.19	0.41	0.07	(0.27,0.54)	2.3
Nabinagar	0.66	0.16	0.61	0.08	0.19	0.62	0.071	(0.48,0.76)	2.27
Obra	0.46	0.16	0.63	0.08	0.19	0.6	0.07	(0.46,0.74)	2.3
Rafiganj	0.6	0.16	0.58	0.08	0.2	0.58	0.072	(0.44,0.72)	2.24
**Aurangabad District Average**	**0.51**	**0.05**	**0.58**	**0.02**		**0.57**	**0.022**	**(0.52,0.61)**	**2.3**
Baikunthpur	0.58	0.21	0.66	0.08	0.12	0.65	0.072	(0.51,0.79)	2.94
Barauli	0.52	0.21	0.68	0.08	0.11	0.67	0.071	(0.53,0.81)	3
Bhorey	0.65	0.22	0.84	0.06	0.07	0.83	0.057	(0.72,0.94)	3.83
Bijaipur	0.48	0.22	0.76	0.07	0.09	0.74	0.066	(0.61,0.87)	3.28
Gopalganj Sadar	0.05	0.21	0.53	0.08	0.13	0.47	0.076	(0.32,0.61)	2.79
Hathua	0.58	0.21	0.58	0.08	0.13	0.58	0.075	(0.43,0.73)	2.83
Kateya	0.6	0.21	0.63	0.08	0.12	0.63	0.073	(0.48,0.77)	2.89
Kuchaikote	0.4	0.21	0.55	0.08	0.13	0.53	0.075	(0.39,0.68)	2.81
Manjha	0.3	0.21	0.53	0.08	0.13	0.5	0.076	(0.35,0.65)	2.79
Pach Deuri	0.56	0.22	0.82	0.06	0.08	0.8	0.06	(0.68,0.91)	3.6
Phulwaria	0.5	0.22	0.76	0.07	0.09	0.74	0.066	(0.61,0.87)	3.28
Sidhwalia	0.47	0.21	0.71	0.07	0.11	0.69	0.07	(0.55,0.82)	3.08
Thawe	0.35	0.21	0.63	0.08	0.12	0.6	0.073	(0.45,0.74)	2.89
Uchkagaon	0.44	0.21	0.47	0.08	0.13	0.47	0.076	(0.32,0.62)	2.79

**Gopalganj District Average**	**0.46**	**0.06**	**0.64**	**0.02**		**0.62**	**0.02**	**(0.58,0.66)**	**2.95**

For the proportion of newborns who were visited by any FLW within 24hours of home delivery, the weighting factor ranges from 0.04 to 0.14 in Aurangabad, and from 0.15 to 0.54 in Gopalganj (Table S9). The combined estimate differs from the LQAS estimate by no more than 3%. Standard errors for the HMIS data are 1.4 to 5.2 times larger than those calculated for the combined estimates.

Table S9 Home visit by any FLW within 24 hours of delivery (home deliveries only)

Block	p_{HMIS}	σ_{HMIS}	p_{lqas}	σ_{lqas}	w	$p_{combined}$	$\sigma_{combined}$	95% CI	σ_HMIS / $\sigma\_combined$
Aurangabad Sadar	0.02	0.13	0.03	0.03	0.04	0.03	0.025	(0,0.08)	5.18
Barun	0.16	0.12	0.11	0.05	0.14	0.11	0.046	(0.02,0.2)	2.7
Daudnagar	0	0.13	0.03	0.03	0.04	0.03	0.025	(0,0.08)	5.18
Deo	0.01	0.13	0.08	0.04	0.11	0.07	0.041	(0,0.15)	3.08
Goh	0.03	0.13	0.05	0.04	0.07	0.05	0.035	(0,0.12)	3.71
Haspura	0.1	0.12	0.11	0.05	0.14	0.11	0.046	(0.01,0.2)	2.7
Kutumba	0.08	0.13	0.05	0.04	0.07	0.05	0.035	(0,0.12)	3.71
Madanpur	0.18	0.13	0.05	0.04	0.07	0.06	0.035	(0,0.13)	3.71
Nabinagar	0.4	0.13	0.03	0.03	0.04	0.04	0.025	(0,0.09)	5.18
Obra	0.12	0.13	0.03	0.03	0.04	0.03	0.025	(0,0.08)	5.18
Rafiganj	0.23	0.13	0.08	0.04	0.11	0.1	0.041	(0.01,0.18)	3.08
**Aurangabad District Average**	**0.14**	**0.04**	**0.06**	**0.01**		**0.06**	**0.011**	**(0.04,0.08)**	**3.68**
Baikunthpur	0.09	0.05	0.08	0.04	0.42	0.08	0.033	(0.02,0.15)	1.54
Barauli	0.06	0.05	0.08	0.04	0.42	0.07	0.033	(0,0.14)	1.54
Bhorey	0.18	0.06	0.03	0.03	0.15	0.05	0.024	(0,0.1)	2.6
Bijaipur	0.14	0.06	0.03	0.03	0.15	0.04	0.024	(0,0.09)	2.6
Gopalganj Sadar	0.05	0.06	0.05	0.04	0.29	0.05	0.031	(0,0.11)	1.86
Hathua	0.06	0.05	0.08	0.04	0.42	0.07	0.033	(0,0.13)	1.54
Kateya	0.13	0.06	0.05	0.04	0.29	0.07	0.031	(0.01,0.13)	1.86
Kuchaikote	0.07	0.06	0.03	0.03	0.15	0.03	0.024	(0,0.08)	2.6
Manjha	0.09	0.05	0.11	0.05	0.54	0.1	0.034	(0.03,0.16)	1.36
Pach Deuri	0.11	0.06	0.05	0.04	0.29	0.07	0.031	(0.01,0.13)	1.86
Phulwaria	0.08	0.06	0.05	0.04	0.29	0.06	0.031	(0,0.12)	1.86
Sidhwalia	0.16	0.05	0.08	0.04	0.42	0.11	0.033	(0.05,0.18)	1.54
Thawe	0.03	0.06	0.05	0.04	0.29	0.05	0.031	(0,0.11)	1.86
Uchkagaon	0.12	0.06	0.03	0.03	0.15	0.04	0.024	(0,0.09)	2.6

**Gopalganj District Average**	**0.09**	**0.02**	**0.06**	**0.01**		**0.06**	**0.008**	**(0.05,0.08)**	**1.91**
