# Supplementary material for: Innovative approach to improve information accuracy in a two-district cross-sectional study in Bihar, India
Source: BMJ Open. 2022 Jan 6;12(1):e051427. doi: 10.1136/bmjopen-2021-051427 (PMC8739057; doi:10.1136/bmjopen-2021-051427)
Supplement: Author's manuscript [file bmjopen-2021-051427.draft_revisions.pdf]

BMJ Open

BMJ Open is committed to open peer review. As part of this commitment we make the peer review history of every article we publish publicly available.

When an article is published we post the peer reviewers' comments and the authors' responses online. We also post the versions of the paper that were used during peer review. These are the versions that the peer review comments apply to.

The versions of the paper that follow are the versions that were submitted during the peer review process. They are not the versions of record or the final published versions. They should not be cited or distributed as the published version of this manuscript.

BMJ Open is an open access journal and the full, final, typeset and author-corrected version of record of the manuscript is available on our site with no access controls, subscription charges or pay-per-view fees (http://bmjopen.bmj.com).

If you have any questions on BMJ Open's open peer review process please email info.bmjopen@bmj.com

BMJ Open

Bad information can lead to misguided health programmes that could cost lives of mothers and children: a two-district cross-sectional study in Bihar, India to test an innovative approach to improve information accuracy

Journal:	*BMJ Open*
Manuscript ID	bmjopen-2021-051427
Article Type:	Original research
Date Submitted by the Author:	22-Mar-2021
Complete List of Authors:	Jeffery, Caroline; Liverpool School of Tropical Medicine, Department of International Public Health<br>Pagano, Marcello; Harvard TH Chan School of Public Health, Department of Biostatistics<br>Devkota, Baburam; Liverpool School of Tropical Medicine, Department of International Public Health<br>Valadez, Joseph; Liverpool School of Tropical Medicine, Department of International Public Health
Keywords:	Health policy < HEALTH SERVICES ADMINISTRATION & MANAGEMENT, Public health < INFECTIOUS DISEASES, Health informatics < BIOTECHNOLOGY & BIOINFORMATICS, International health services < HEALTH SERVICES ADMINISTRATION & MANAGEMENT

SCHOLARONE™
Manuscripts

I, the Submitting Author has the right to grant and does grant on behalf of all authors of the Work (as defined in the below author licence), an exclusive licence and/or a non-exclusive licence for contributions from authors who are: i) UK Crown employees; ii) where BMJ has agreed a CC-BY licence shall apply, and/or iii) in accordance with the terms applicable for US Federal Government officers or employees acting as part of their official duties; on a worldwide, perpetual, irrevocable, royalty-free basis to BMJ Publishing Group Ltd ("BMJ") its licensees and where the relevant Journal is co-owned by BMJ to the co-owners of the Journal, to publish the Work in this journal and any other BMJ products and to exploit all rights, as set out in our licence.

The Submitting Author accepts and understands that any supply made under these terms is made by BMJ to the Submitting Author unless you are acting as an employee on behalf of your employer or a postgraduate student of an affiliated institution which is paying any applicable article publishing charge ("APC") for Open Access articles. Where the Submitting Author wishes to make the Work available on an Open Access basis (and intends to pay the relevant APC), the terms of reuse of such Open Access shall be governed by a Creative Commons licence – details of these licences and which Creative Commons licence will apply to this Work are set out in our licence referred to above.

Other than as permitted in any relevant BMJ Author's Self Archiving Policies, I confirm this Work has not been accepted for publication elsewhere, is not being considered for publication elsewhere and does not duplicate material already published. I confirm all authors consent to publication of this Work and authorise the granting of this licence.

For peer review only

Bad information can lead to misguided health programmes that could cost lives of mothers and children: a two-district cross-sectional study in Bihar, India to test an innovative approach to improve information accuracy

Authors: Caroline Jeffery[1], Marcello Pagano[2], Baburam Devkota[1], Joseph J Valadez[1]

[1] Department of International Public Health, Liverpool School of Tropical Medicine, Pembroke Place, Liverpool, UK

[2] Department of Biostatistics, Harvard T.H. Chan School of Public Health, 677 Huntington Avenue, Boston, MA, USA

Corresponding author: Joseph J Valadez, joseph.valadez@lstmed.ac.uk

For peer review only

ABSTRACT

OBJECTIVE

Combine Health Management Information Systems (HMIS) and probability survey data using the statistical annealing technique (AT) to produce more accurate health coverage estimates than either source of data and a measure of HMIS data error.

SETTING

An important source of data, routinely reported by health facilities and used by health professionals for programme decision-making is HMIS data. Its quality is sometimes poor or unknown, and there is no measure of its uncertainty. Using AT we combine district-level HMIS and probability survey data (n=475) for the first time for 10 indicators assessing antenatal care, institutional delivery and neonatal care from 11 blocks of Aurangabad and 14 blocks of Gopalganj districts (N=6,253,965) in Bihar State, India.

PARTICIPANTS

Both districts are rural. Bihar is 82.7% Hindu and 16.9% Islamic.

PRIMARY OUTCOME MEASURES

Survey prevalence measures for 10 indicators, corresponding prevalences using HMIS data, combined prevalences calculated with AT, and Standard Errors for each type of data.

RESULTS

The combined and survey estimates differ by <0.10. The combined and HMIS estimates differ by up to 84.2%, with the HMIS having 1.4 to 32.3 times larger error. Of 20 HMIS versus survey coverage estimate comparisons across the two districts only five differed by <0.10. Of 250 sub-district-level

For peer review only

For peer review only - http://bmjopen.bmj.com/site/about/guidelines.xhtml

comparisons of HMIS versus combined estimates, only 36.4% of the HMIS estimates are within the 95%CI of the combined estimate.

CONCLUSIONS

Our statistical innovation increases the accuracy of information available for local health system decision-making, allows evaluation of indicator accuracy, and increases the accuracy of HMIS estimates. The combined estimates with a measure of error better informs health system professionals about their risks when using HMIS estimates, so they can reduce waste by making better decisions. Our results show that AT is an effective method ready for use internationally to provide affordable information to improve health services.

STRENGTHS AND LIMITATIONS OF THIS STUDY

- Strength: household survey data were captured as a stratified random sample with no apparent design effect leading to an efficient use of information.
- Strength: administrative data comprise 100% of the available recurrent information available in the two selected districts.
- Strength: the study population is very large covering a large geographical area, reducing the likelihood that the results are pertinent only to a small group of mothers with children; results may be generalizable.
- Strength: the process for combining probability and administrative data has been rigorously assessed prior to use in this study.
- Limitation: the study is confined to two districts of Bihar, India which indicates the need for replicating the study in additional States of India and in other country settings.

KEY WORDS

HMIS, recurrent information systems, hybrid prevalence, combining data, prevalence, indicators

For peer review only - http://bmjopen.bmj.com/site/about/guidelines.xhtml

INTRODUCTION

The 17 Sustainable Development Goals (SDGs) were adopted by all United Nations member states in 2015 as an urgent call for action to end poverty and deprivation by following strategies that improve health and education, reduce inequality and spur economic growth. For national strategies to be effective, they must be grounded in the local context. Good quality data to measure the prevalence of disease conditions, or the population's coverage with health services coverage, is an indispensable resource for programme managers and health policy makers to understand their context. This point is equally true in higher income countries as it is in low and middle-income countries (LMIC). In these latter settings, elaborate household surveys are generally considered resource depleting and seldom done. As a result, reliance on data generated routinely through the Health Management Information Systems (HMIS) is commonly the basis for decision-making. HMIS data consists of routine information reported from the health facilities to districts health offices, who submit it to Ministry of Health on a monthly, quarterly, semi-annual and annual basis. A key shortcoming of HMIS data is that it is only representative of those who can access health services. This shortcoming is especially problematic in LMIC where access to healthcare services is constrained by geographic, economic and social determinants.

Demand by global donors for timely and reliable data continues to increase[1]. The Sixtieth World Health Assembly underscored the importance of robust information to strengthen health systems and policies (resolution WHA60.27)[2], and to this end, established a Health Metrics Network partnership to aid countries improve data quality of routine information systems to enable their use for health planning and decision-making[3-6]. The advent of electronic health records was heralded, as studies showed their association with improved clinical care, outcomes[7], and surveillance[8]. Consequently, the advancement of a computerised District Health Information System-2 (DHIS2) was implemented in 40+ countries to integrate data sources for their more rapid analyses, dissemination and use. An evaluation in Tanzania showed it improved timeliness and completeness of reporting[8]. South Africa established a dedicated HIV/AIDS information system recommending the use of the

For peer review only - http://bmjopen.bmj.com/site/about/guidelines.xhtml

earlier DHIS for integrating different information sources[9] and as input for designing national program strategies[10]. A 2010-2015 study in Swaziland recommended embracing electronic medical data systems to reduce the discrepancies occurring between the existing three information systems put in place for malaria elimination[11]. Recently, a similar demand was issued by practitioners working in humanitarian settings for an *epidemiology and demography service* to develop robust and timely information from multiple sources to establish priorities that address the population's needs[12].

One important use of routine information systems is to monitor a population's coverage with health service, as Global Alliance on Vaccines and Immunisations (GAVI) has done for vaccination rates since the 1990s. They attempt to control for known weaknesses in administrative data by carrying out data quality audits by recounting, and contrasting, the number vaccinated in health centres with the number originally reported[13].

Although measurement improves all facets of health management, HMIS related studies mainly focus on improvements in obtaining numerators[1 5 14 15]. The future also demands accurate and precise denominators, and, more importantly, a principled method for assessing their accuracy (e.g., standard errors (SE)) so users understand their risk when using the data. Under normal circumstances, the error present in HMIS remains unknown.

The research question we investigate in this paper: How can we measure the error in the HMIS and improve its accuracy? We address this issue by presenting an innovation called *Statistical Annealing* (Annealing Technique or AT). It builds on our earlier pioneering work in 2011[16] and refined in 2018 [17] that provides an estimator with a narrower 95% Confidence Interval (CI) by combining data from a probability survey and HMIS data. We demonstrate the magnitude of HMIS error and how it can be improved by using AT.

By the early 20th century Bowley[18] and Neyman[19] proved the importance of sampling and the estimated magnitudes of their errors. The standard error (SE)[20] is a commonly used measure of

For peer review only - http://bmjopen.bmj.com/site/about/guidelines.xhtml

error, which is an estimate of deviation of the sample mean from the actual population mean. The SE is used in calculating a 95% Confidence Interval (CI), which relates to the accuracy of the estimate. If survey data are combined (annealed) with HMIS appropriately they produce a single, more accurate coverage estimator[16]. However, concurrent well-designed surveys are a surprisingly neglected sources of information for improving HMIS [21 22].

The objective of AT is to produce a principled measure of health system performance by combining existing HMIS and survey information which also produces an accompanying measure of its accuracy. This hybrid estimate is more accurate than either data source alone. Our proof of principle study, used Child Health Day (CHD) administrative data from Benin and Madagascar provided by UNICEF country offices and household survey data collected at the same time, to verify CHD coverage and the quality of CHD administrative data [23]. This refined approach to AT resulted in the production of a 95% CI for the administrative data and for the combined result. See Supplementary Information 1 for the AT formulae.

The current study assesses the transferability of AT to a new setting, HMIS data from Bihar, India. Also, this study is the first use of AT with true HMIS data, which in the future will be a more typical use for AT, and result in our conclusions about its global applicability. We apply AT to 10 indicators related to antenatal care (ANC), institutional delivery, and neonatal care using data from two districts, Aurangabad (N=3,695,928) and Gopalganj (N=2,558,037).

METHODS

HMIS system in Bihar

The HMIS data in Bihar consists of 318 data elements. These are reported monthly by health sub-centres (HSC), and aggregated at the block primary health centre (BPHC). The *block* is the sub-district administrative unit in India. Staff at the HSCs compile paper registers which they send to the BPHCs where it is entered into a computerized HMIS system, and subsequently maintained electronically.

For peer review only - http://bmjopen.bmj.com/site/about/guidelines.xhtml

District-level indicators comprise information aggregated across the block as well as data from district and referral hospitals.

Data

To develop AT in Bihar, we used the health facility-based HMIS and probability data from a household survey in two districts, Aurangabad and Gopalganj. From the Bihar State Health Society, we obtained two sources of HMIS data: the HMIS data reported by all health facilities in the two districts, at district level and disaggregated to the block-level; and the Expected Level of Achievement (ELA), which is projected data. They calculated the ELA data using the latest 2011 census, and applied an average annual population growth rate using parameter values reported in the Annual Health Survey 2012-13 (e.g., crude birth rate) and in the HMIS data from previous years (e.g., percentage of pregnancies leading to a still birth in 2015-16). We averaged these two data sources, the HMIS and the ELA, to obtain the administrative estimate.

A household probability survey was conducted during 7-25 September 2016 in each district in a standardised manner[24 25] using Lot Quality Assurance Survey (LQAS). The LQAS survey is a two-stage stratified random sample used to assess health service coverage. The strata are administrative blocks (11 in Aurangabad, 14 in Gopalganj), which are sub-district units in India. The strata were weighted by their population size. The first stage sample uses probability proportional to size to randomly sample villages (typically, n=19 per stratum[24]). In each random location a second stage sample identifies an index household using segmentation sampling[26 27] from locally constructed hand-drawn maps. The next closest household is then selected for interview to reduce the likelihood that households not included in the map do not have zero probability of selection. In the selected household, individuals in three target groups (women with children 0-2, 3-5 and 0-5 months of age[28]) are listed with one selected randomly using a random number table. The remaining target group is selected in the next closest house using the same protocol[29]. The resulting sample of individuals is

For peer review only - http://bmjopen.bmj.com/site/about/guidelines.xhtml

random and provides its own standard error (SE) estimator used for calculating a 95% CI. One member of each target group only was selected in a sampled village.

The total number of random samples for each indicator is 209 (Aurangabad) and 266 (Gopalganj). LQAS gives reliable estimates for indicators at the district level[30] and allows the classification of blocks, as “performing adequately” or not. However, in this paper we do not use the data for classification, but rather for estimation.

Indicators for annealing

We based the selection of 10 indicators for annealing on the ease of combining HMIS data temporally to the survey data (Table 1). For the LQAS survey, we measured antenatal care (ANC), institutional delivery, and neonatal care outcomes in the population of women with children 0-2, 3-5 and 0-5 months of age[28] resulting in a total of 627 (Aurangabad) and 798 (Gopalganj) interviews. We used the HMIS data from the last 5-months of reports (April-August 2016) preceding the LQAS survey (Table 1), so that they matched temporally.

The Annealing Technique design

As in earlier work [23], we apply AT in each sub-district for each indicator. The district-level estimators are an aggregation of appropriate block-level coverage data, weighted by the population sizes. Data analysis was done using Stata SE ver15 and R ver-3.4.1.

Survey coverage estimator

For all but two indicators, the numerator of the LQAS coverage is the sum of correct responses in each of two target group (mothers of children 0-2 months and 0-5 months) in each block. The denominator is the sum of the denominators of both groups $(19 \times 2 = 38)$. For the two indicators we used additional data from a third target group, mothers of children 3-5 months, where the denominator is the sum of the denominators of each target group $(19 \times 3 = 57)$.

For peer review only - http://bmjopen.bmj.com/site/about/guidelines.xhtml

A step in the annealing process requires a weighted average of component parts; the LQAS estimator is one such component. For each component, the weight used is inversely proportional to its variance. If the LQAS coverage, p, is away from the extremes of zero or one, we can use the standard binomial formula, $p(1-p)/n$. When the LQAS coverage is zero (or one), we calculate the weight by using a surrogate `standard error' from a 95% CI using Louis' approach[31] and dividing its length (1-0.05$^{(1/n)}$) by (2×1.96), and squaring the resultant.

To assess any block-level clustering effect between target groups sampled in parallel, we use the McNemar exact test for correlated proportions for each indicator[32], across all blocks sampled in each district.

HMIS coverage estimator

The numerator of the HMIS coverage is reported in Table 1 (Column 4). The denominator is the average between the total number of ANC registration per month calculated by the ELA projected data and those reported in the HMIS data (averaged over April-August 2016).

For peer review only - http://bmjopen.bmj.com/site/about/guidelines.xhtml

1 Table 1: List of 10 indicators selected for applying the annealing technique in Bihar State, India

Generic name of Indicator	LQAS Indicator	Targeted groups (LQAS surveys)	HMIS indicator	Targeted months (HMIS reported data)
First trimester registration	Proportion of mothers of 0-2 months old whose pregnancy was registered in the first trimester in the last pregnancy	0-2 months<br>0-5 months	Number of pregnant women registered within first trimester	April-August 2016
3 or more ANC	Proportion of mothers of 0-2 months old who had 3 or more ANC's during their entire period of last pregnancy	0-2 months<br>0-5 months	Number of pregnant women received 3 ANC check-ups during pregnancy	April-August 2016
Received TT1	Proportion of mothers of 0-2 months who received TT1 during their last pregnancy	0-2 months<br>0-5 months	Number of pregnant women given TT1 during current pregnancy	April-August 2016
Received TT2	Proportion of mothers of 0-2 months who received TT2 during their last pregnancy	0-2 months<br>0-5 months	Number of pregnant women given TT2 or booster during current pregnancy	April-August 2016
Received 100 IFA tablets	Proportion of mothers of 0-2 months who received at least 100 IFA tablets during their last pregnancy	0-2 months<br>0-5 months	Total number of pregnant women given 100 IFA tablets	April-August 2016
Institutional deliveries (all)	Proportion of 0-3 months infants who had institutional delivery (survey conducted September 2016)	0-2 months<br>3-5 months<br>0-5 months	Total number of deliveries conducted in institutional setting (private and public) including C sections	April-August 2016

For peer review only - http://bmjopen.bmj.com/site/about/guidelines.xhtml

Public Institutional Deliveries	Proportion of 0-3 months infants who had institutional delivery at public facility (survey conducted September 2016)	0-2 months<br>3-5 months<br>0-5 months	Number of deliveries conducted in public institutions including C sections	April-August 2016
Baby weighed during delivery	Proportion of 0-2 months infants who were weighed during delivery	0-2 months<br>0-5 months	Number of new-borns weighed at birth	April-August 2016
Early Initiation of Breast feeding (EIBF)	Proportion of 0-2 months infants who were breast fed within an hour of their birth	0-2 months<br>0-5 months	Number of new-borns breast fed within one hour of birth	April-August 2016
Post-Partum home visit within 24 hours (home-deliveries only)	Home visit by any FLW within 24 hours of delivery	0-2 months<br>0-5 months	Number of new-borns visited within 24 hours of delivery for deliveries conducted at home.	April-August 2016

For peer review only

To calculate the weight, as we did for the LQAS estimator, to use for the HMIS estimator, we use the fact that both estimators are estimating the same quantity, so their difference should be estimating zero. This can then be used to calculate the weight, as we show in [23] and in the Supplementary Information 2 (Tables S1-S9).

Assessing variation in local-level data quality

To advance the use of AT in multiple settings we developed a tool for sub-district-level analysis. We assess the variation in the quality of the HMIS indicators in 250 block-level comparisons of the 10 indicators $(10 \times (11\ Aurangabad + 14\ Gopalganj))$ and 20 district-level comparisons. We classify the value of the HMIS coverage relative to the CI around the combined estimate. If the HMIS coverage is within 95% CI of $p_{combined}$ the cell is coloured green. If the HMIS estimate is within a reasonable percentage difference of each boundary of the CI, for example 10% (we colour those cells light red if it is above the high boundary or light blue if it is below the lower boundary), we are assured its value is not far from the survey estimate. While when the HMIS coverage is beyond the reasonable percentage difference of each boundary, the discrepancy might be too large to recommend combining the two estimates and to not use the HMIS estimate (we colour these dark red or dark blue).

Ethics Statement

The study was approved by the Indian Institute of Public Health Bhubaneswar Institutional Ethics Committee (IIPHB-IEC-2016/010) and the Ethical Review Board of the Liverpool School of Tropical Medicine (research protocol 16-023RS). We obtained oral rather than written informed consent from all respondents, because of the high illiteracy rate.

For peer review only - http://bmjopen.bmj.com/site/about/guidelines.xhtml

Patient and Public Involvement

This study does not involve patients. Also, the public were not involved in the design, conduct and reporting of the research. The public was engaged as interviewees. To ensure local engagement all data capture was carried out in close coordination with the State Ministry of Health of Bihar. We also shared the results with them and offered further dissemination of results, and engaged them for data use and action planning activities.

RESULTS

Data characteristics and AT characteristics

Among the 10 indicators, the Mean Squared Error (MSE) between the two sources of data ranges from 0.013 to 0.193 in Aurangabad, and between 0.005 and 0.391 in Gopalganj (See Table 2 for the indicators). Three blocks in Gopalganj have an HMIS coverage larger than 100% (two or more tetanus toxoid vaccinations (TT2): 104% in Barauli and 103% in Kateya; iron folic acid (IFA) tablets distribution: 147% in Pach Deuri). The HMIS coverage averaged over blocks ranges between 12% and 80% in Aurangabad, and between 10% and 84% in Gopalganj. The LQAS coverage averaged over the blocks ranges between 3% and 98% in Aurangabad, and between 5% and 97% in Gopalganj. The McNemar tests did not detect block-level clustering except for the four tests with indicators measuring institutional delivery in all facilities or in public facilities.

For peer review only

Table 2: Summary information before application of AT

District	Indicator	MSE	Average value of p_{HMIS} over all blocks†	Average value of p_{LQAS} over all blocks†	McNemar test p-values ‡
Aurangabad	Pregnancy registered during first 3 months	0.105	0.66	0.42	0.47
	3 or more ANC	0.013	0.64	0.62	0.46
	Mothers who were protected against tetanus during pregnancy (Neonatal TT)	0.152	0.61	0.98	0.71
	Mothers who were protected against tetanus TT2	0.027	0.79	0.67	0.68
	Mothers who received iron folic acid for 100 days or more when they were pregnant	0.150	0.38	0.03	0.71
	Institutional delivery (Both private and public facility)	0.193	0.34	0.74	0-2 vs 3-5mo: 0.73<br>0-2 vs 0-5mo: 0.39<br>0-5 vs 3-5mo: 0.20
	Institutional delivery (public facility)	0.060	0.34	0.53	0-2 vs 3-5mo: 0.05<br>0-2 vs 0-5mo: 0.84<br>0-5 vs 3-5mo: 0.04[§]
	Baby Weighed within one hour of birth	0.078	0.50	0.67	0.65
	Early Initiation of Breast feeding (EIBF)	0.032	0.50	0.57	0.49
	Home visit by any FLW within 24 hours of delivery	0.018	0.12	0.06	0.18
Gopalganj	Pregnancy registered during first 3 months	0.078	0.72	0.48	0.86
	3 or more ANC	0.051	0.73	0.63	0.37

For peer review only

For peer review only - http://bmjopen.bmj.com/site/about/guidelines.xhtml

	Mothers who were protected against tetanus during pregnancy (Neonatal TT)	0.065	0.76	0.97	0.81
	Mothers who were protected against tetanus TT2 *	0.031	0.84	0.78	0.91
	Mothers who received iron folic acid for 100 days or more when they were pregnant *	0.391	0.63	0.05	0.20
	Institutional delivery (Both private and public facility)	0.245	0.35	0.85	0-2 vs 3-5mo: 0.04§ 0-2 vs 0-5mo: 0.06 0-5 vs 3-5mo: 0.89
	Institutional delivery (public facility)	0.068	0.35	0.59	0-2 vs 3-5mo: 0.01§ 0-2 vs 0-5mo: 0.03§ 0-5 vs 3-5mo: 0.51
	Baby Weighed within one hour of birth	0.117	0.44	0.75	0.05
	Early Initiation of Breast feeding (EIBF)	0.051	0.45	0.65	0.17
	Home visit by any FLW within 24 hours of delivery	0.005	0.10	0.05	0.51

* Three blocks in Gopalganj have an HMIS coverage larger than 100% (TT2 indicator: 104% in Barauli and 103% in Kateya; IFA tablets indicator: 147% in Pach Deuri)

† 11 blocks in Aurangabad and 14 blocks in Gopalganj.

‡ McNemar test comparing 0-2 vs 0-5 months unless otherwise specified.

§ The McNemar test reports a major difference at $p < 0.05$.

For peer review only - http://bmjopen.bmj.com/site/about/guidelines.xhtml

The weighting factor used in the calculation of the combined estimator varies for each indicator in each district (Figure 1). For six indicators (registration during first trimester of pregnancy, receiving TT1, receiving ≥100 IFA tablets, institutional delivery in any facility, deliveries in public facilities, baby weighed within 1 hour of birth), all block-level weighting factors *w*, are below 0.10 in both districts, indicating the contribution of HMIS estimator to the combined estimator is no higher than 10% (Table 3 and Supplementary Information 2 Tables S1-S9). We use a 1-tail test to check for difference of the weights between the two districts, based on the observed mean difference. The weights for Gopalganj are higher than Aurangabad's for three indicators: first trimester registration, receiving TT1 and having a post-partum visit – for all three, one-sided t-test p-values are <0.01. The Gopalganj weighting factors are lower than Aurangabad's for the other seven indicators: ≥3 ANC visits, receiving TT2, receiving ≥100 IFA tablets, institutional delivery in any facility, deliveries in public facilities, early initiation of breastfeeding (EIBF), baby weighed at delivery (all seven one-sided t-test p-values <0.05). This result indicates that there are more discrepancies between the HMIS estimates and the LQAS estimates in Aurangabad when compared to Gopalganj for the first three indicators, while the discrepancies are greater in Gopalganj for the latter seven indicators. All but one of the weights for the indicator ≥3 ANC visits in Aurangabad are between 0.4 and 0.5, indicating an almost equal contribution of the HMIS and LQAS estimators to the resulting combined estimator. Alternatively, there are large variations in the weights for the indicator measuring the one visit by any Frontline Worker (FLW) within 24-hours of delivery in Gopalganj; while, the MSE is small in this instance.

(Insert Figure 1 here)

[Footnote and Title for Figure 1: *Circles with no filling indicate outlier values

Figure 1: Distribution and average of *w* for each indicator and district*]

For peer review only

For peer review only - http://bmjopen.bmj.com/site/about/guidelines.xhtml

Table 3: Women who registered for ANC during the first three months of pregnancy

Block	p_{HMIS}	σ_{HMIS}	p_{lqas}	σ_{lqas}	w	$p_{combined}$	$\sigma_{combined}$	95% CI	$\sigma_{HMIS}/\sigma_{com}$
Aurangabad Sadar	0.42	0.31	0.39	0.08	0.06	0.4	0.077	(0.25,0.55)	4.08
Barun	0.55	0.31	0.58	0.08	0.06	0.58	0.078	(0.43,0.73)	4.04
Daudnagar	0.67	0.31	0.53	0.08	0.06	0.54	0.078	(0.38,0.69)	3.99
Deo	0.65	0.31	0.55	0.08	0.06	0.56	0.078	(0.41,0.71)	4.01
Goh	0.75	0.32	0.18	0.06	0.04	0.21	0.062	(0.08,0.33)	5.14
Haspura	0.46	0.31	0.55	0.08	0.06	0.55	0.078	(0.39,0.70)	4.01
Kutumba	0.78	0.31	0.42	0.08	0.06	0.44	0.078	(0.29,0.60)	4.04
Madanpur	0.74	0.31	0.32	0.08	0.05	0.34	0.073	(0.20,0.48)	4.29
Nabinagar	0.81	0.31	0.37	0.08	0.06	0.39	0.076	(0.25,0.54)	4.13
Obra	0.68	0.31	0.5	0.08	0.06	0.51	0.079	(0.36,0.67)	3.99
Rafiganj	0.77	0.32	0.26	0.07	0.05	0.29	0.070	(0.15,0.42)	4.53
**Aurangabad District Average**	**0.68**	**0.1**	**0.41**	**0.02**		**0.42**	**0.023**	**(0.38,0.47)**	**4.23**
Baikunthpur	0.73	0.27	0.53	0.08	0.08	0.54	0.078	(0.39,0.70)	3.46
Barauli	0.74	0.27	0.39	0.08	0.08	0.42	0.076	(0.27,0.57)	3.53
Bhorey	0.66	0.27	0.53	0.08	0.08	0.54	0.078	(0.39,0.69)	3.46
Bijaipur	0.81	0.27	0.47	0.08	0.08	0.5	0.078	(0.35,0.65)	3.46
Gopalganj Sadar	0.67	0.27	0.37	0.08	0.08	0.39	0.075	(0.24,0.54)	3.58
Hathua	0.94	0.27	0.71	0.07	0.07	0.73	0.071	(0.59,0.87)	3.81
Kateya	0.89	0.27	0.39	0.08	0.08	0.43	0.076	(0.29,0.58)	3.53
Kuchaikote	0.74	0.27	0.39	0.08	0.08	0.42	0.076	(0.27,0.57)	3.53
Manjha	0.67	0.27	0.61	0.08	0.08	0.61	0.076	(0.46,0.76)	3.53
Pach Deuri	0.49	0.27	0.47	0.08	0.08	0.47	0.078	(0.32,0.63)	3.46
Phulwaria	0.61	0.27	0.5	0.08	0.08	0.51	0.078	(0.36,0.66)	3.45
Sidhwalia	0.79	0.27	0.42	0.08	0.08	0.45	0.077	(0.30,0.60)	3.5
Thawe	0.67	0.27	0.42	0.08	0.08	0.44	0.077	(0.29,0.59)	3.5
Uchkagaon	0.71	0.27	0.42	0.08	0.08	0.44	0.077	(0.29,0.59)	3.5
**Gopalganj District Average**	**0.73**	**0.08**	**0.48**	**0.02**		**0.5**	**0.022**	**(0.46,0.54)**	**3.53**

Comparing indicator estimates of 20 p_{HMIS} versus p_{LQAS} across the two districts, five differed by $\leq$0.10 and six others by $\leq$0.20. The remaining nine indicator estimates show larger differences (Table 3; Tables S1-S9).

For peer review only - http://bmjopen.bmj.com/site/about/guidelines.xhtml

ANC services

All estimates, SEs and CIs are calculated at the block and district levels (as an illustration, see the detailed results for first trimester ANC registration in Table 3; Supplementary Information 2 Tables S1-S4 for other indicators). For the five indicators measuring coverage related to ANC and birth preparedness, the combined block-level estimate differs from the LQAS estimate by 1% (received ≥100 IFA tablets) to 10% (≥3 ANC visits); the block-level combined estimate differs from the HMIS estimate at most by 28% (TT1) to 133% (received ≥100 IFA tablets). SEs for the HMIS data are 1.4 to 32.3 times larger than those calculated for the combined estimates.

Institutional delivery

For the two indicators measuring the proportion of infants institutionally delivered (Tables S5-S6), the combined block-level estimate differs from the LQAS estimate at most by 1% (all facilities) and 4% (public facilities only); the combined estimate differs from the HMIS estimate at most by 84% (all facilities) and 58% (public facilities only). SEs for the HMIS data are 3.7 to 14.6 times larger than those calculated for the combined estimates.

Neonatal health

For the three indicators measuring coverages related to neonatal health (Supplementary Information 2 Tables S7-S9), the combined block-level estimate differs from the LQAS estimate at most by 3% (newborns who were visited by any FLW within 24-hours of home delivery) to 8% (EIBF); the combined estimate differs from the HMIS estimate at most by 36% (newborns who were visited by any FLW within 24-hours of home delivery) to 70% (EIBF). SEs for the HMIS data are 1.4 to 6.2 times larger than those calculated for the combined estimates.

For peer review only

For peer review only - http://bmjopen.bmj.com/site/about/guidelines.xhtml

Local level variation

We first look at the two rows of Figure 2 that summarize the district level measures and see that, on average, Aurangabad's HMIS measures (row 13) behave slightly better than Gopalganj's (row 28). We see that for two indicators both districts are red; for three they are both blue; for two they are both light red; and for two Aurangabad's are light blue versus Gopalganj's dark blue; and, for one Aurangabad's is green and Gopalganj's is light red.

(Insert Figure 2 here)

[Footnote and Title for Figure 2:

*5 categories: (Blue) HMIS coverage is more than 10% below the lower bound. (Light blue) HMIS coverage is less than 10% below the CI lower bound. (Green) HMIS coverage is within the CI. (Light red) HMIS coverage is less than 10% above the CI upper bound, (Red) HMIS coverage is more than 10% above the CI upper bound.

Figure 2: HMIS coverage value compared to the 95% CI of the $p_{combined}$ estimate for 10 Indicators*]

We get a more detailed contrast when investigating the block-level results. Of the 250 comparisons 36.4% of the HMIS estimates are within the CI of the combined estimate with Aurangabad displaying greater accuracy (44.5%) than Gopalganj (30%). For two indicators (100 IFA tablets received and facility based delivery (all)) the results are consistently poor across blocks. The same can be said of TT1 in Aurangabad, but not in Gopalganj. These indicators warrant further investigation into why the two sources of data have such discrepancies. 3+ANC displays good agreement in Aurangabad, but a mixed evaluation in Gopalganj. Similarly, with the remaining indicators, we can detect subtly different results within the two districts. These results suggest substantial variation across blocks, districts and indicators in the HMIS accuracy.

For peer review only - http://bmjopen.bmj.com/site/about/guidelines.xhtml

DISCUSSION

The results of our study show numerous discrepancies of block-level HMIS coverage estimates in both districts compared to the probability samples. However, the extent of these discrepancies vary across the blocks. We would expect consistency among block-level results given their geographic and political proximity within these two districts. Using the combined estimates as our guides, they differ from the respective probability estimates by at most 10%. In contrast, the combined estimates differ from the HMIS estimates by up to 84.2% (FBD(all) in Aurangabad's block Sadar), except for one instance where the difference is 133% due to the administrative coverage being >>100% (distribution IFA during pregnancy in Pach Deuri). SEs for the HMIS estimates are between 1.4 and 32.3 times larger than for the combined estimates (Supplementary Information 2 Tables S1, S9 and S4).

The HMIS data provides two sources of information to calculate the denominator (reported HMIS and Expected Level of Achievement-ELA). Across the 14 blocks of Gopalganj, the two values were very close. In Aurangabad we observe a linear relationship between the two sets of values, with the ELA values being slightly higher than the HMIS recorded values, suggesting the projected data overestimates the numbers reported. Since there was no evidence that one was better than the other, we chose to take the average of both values to define the denominator of the HMIS estimator. This step can be used in future applications.

A general attractiveness of the HMIS results for management purposes is their results apply at the block-level, and we do not wish to lose this property. We are fortunate because this level also corresponds to the strata in the LQAS surveys, and thus, this is the information level provided by the combined estimates. For greater granularity, LQAS is a favourable method to use for AT. In this study, we were also able to increase the sample size by pooling the data from two or three target populations surveyed in parallel within a block.

Our approach allows us to contrast the two districts of Bihar by studying the block-level (sub-district-level) weighting factors *w*. For example, six indicators across both districts, have weights lower than

For peer review only - http://bmjopen.bmj.com/site/about/guidelines.xhtml

0.10, highlighting a large standard error in the HMIS estimates versus those in the probability sample estimates which leads to a wide 95%CI in the HMIS estimates. The contribution of the HMIS estimator to the combined estimator for the indicator measuring visits by any Frontline Worker (FLW) within 24-hours of birth estimator is higher in Gopalganj; the weights range between 0.15 and 0.54, indicating a more balanced contribution between the convenience and the probability sample, meaning that some indicators have similar estimates while others do not. In Aurangabad the only indicator having a contribution weight of the HMIS estimator of over 0.35 in all blocks is three ANC visits. Hence, we are not observing a *systematic* discrepancy across indicators and districts. Some HMIS estimates are similar to the probability survey estimates for specific indicators, other indicators display similarity in only certain sub-districts or districts, while other indicators show consistent discrepancies between HMIS and survey result estimates. Ex post, we can learn from these concordance and discordance patterns how well some indicators are defined and measured across districts.

These Bihar-HMIS AT results differ from the Benin and Madagascar-CHD AT results. In the latter study the administrative data consistently over-estimated prevalence [23]. Such is not the case here as 25.6% of indicators over-estimate and 38% under-estimate coverage. Future research should map the variation in data quality by country and indicators as well as sub-nationally, and understand the reasons for the discrepancies between the HMIS and probability sample. These discrepancies might be due to one or more reasons: poor quality denominators in the HMIS in the state or in specific districts; inaccurate or incomplete recording of the HMIS numerator data in one district or at the block-level; or incomplete transmission of HMIS records from a health centre to the block-level for aggregation[33]. It is also possible that the survey and HMIS indicators are not measuring quite the same health system product. The perception of a respondent to a survey question in their own home and that of the health worker in a health centre may differ. Such indicators may need replacement as well. Related to this issue is a question of a more general nature. In situations when two estimates differ substantially, is it advisable to combine conflicting estimates? We think this

For peer review only - http://bmjopen.bmj.com/site/about/guidelines.xhtml

issue can be answered by the data: as the estimates are different, a compromise suggested by the data is to give appropriate weight to the disparate estimates when combining them – a principle which we demonstrate. As to the prescription for future strengthening of the HMIS and DHIS2, this is a separate issue the solution of which depends on information obtained from principled future research.

Our AT method uses statistical principles to combine data coming from different sources and calculates error for all sources as well as for the resulting combined estimate. This innovative product is valuable. The AT approach is different to WHO's well-intentioned attempt to improve HMIS estimates with Computation Logic; its estimates rely on professional judgements and do not produce error terms or CIs–a limitation noted by the authors[34]. Therefore, the risk associated with their resultant is unknown. The Institute for Health Metrics and Evaluation applies more complex quantitative analyses rather than just human judgments. It produces an estimate of the number of additional children covered or overestimated[35]. However, like Computational Logic, it does not report a measure of its error.

We are living in an information conscious era with continual demand for, and the ability to produce, more and better data[1 36] obtained from multiple sources. Using accurate information can improve a population's health and reduce waste. We guide the integration of existing data sources to provide a more complete assessment of national health and to reduce the cost to the health system. While we consider numerous data sources, HMIS, which generally do not produce SEs, are touted as having increasing importance for improving the coverage and quality of health services[37 38]. Yet, HMIS are not accurate. Improving and measuring the quality of health information systems is greatly needed[39]. Our examples exemplify a ubiquitous situation, namely, the difficulty with having a good estimator of the denominator. These problems occur in developed and low resource countries[15], leading some policy-makers to call for a restructuring of information systems[39]. The current strategy for improving information involves rolling-out a computerized District Health Information System-2 (DHIS2).

For peer review only - http://bmjopen.bmj.com/site/about/guidelines.xhtml

Though promising, whether the DHIS2 improves data quality is yet to be determined. Nevertheless, its concern is production of quality numerators. Our study shows that AT detected variation in the quality of the HMIS by indicator and sub-district location, and can be used to strengthen systems like the DHIS2 by identifying indicators and their location where improvement is needed. While it does not tell us the reasons for the errors it shows where they exist and their magnitude. This information can be used to understand and correct the sources of errors, which can improve health programmes and reduce waste.

These results indicate the need to establish a service working side by side of the DHIS2 to inculcate hybrid estimation using AT as a standard component of district information systems. It should be an independent partner of the DHIS2, under the stewardship of lead international agencies such as UNICEF, the Global Fund to Fight AIDS, Tuberculosis and Malaria, USAID, World Bank and bi-lateral organisations (USAID, DFID). It would work independently and transparently to advance hybrid estimation not only in support of DHIS2, but also, and possibly more importantly, to support local public health practitioners and national policy-makers focus their programs to address priorities.

CONCLUSION

Our statistical innovation, hybrid prevalence estimation using statistical AT, requires concomitant data from a random survey sample[40] conducted at the sub-district-level, and sub-district-level HMIS data. The LQAS survey in Bihar was undertaken for another purpose and so its use in AT was at no additional expense. Other statistical surveys, including the Demographic and Health Survey, are potential candidates. Provided both datasets are available in multiple sub-districts, we can estimate the variability of each indicator between sub-districts, and thus, construct not only a combined estimate but also its 95%CI at both sub-district and district levels. and a 95%CI for the HMIS estimator. With the new visual tool presented here, AT results can be quickly interpreted.

AT is intended to improve the quality and use of the HMIS while reducing waste due to using inaccurate information. This study shows that bringing data from existing household surveys

For peer review only - http://bmjopen.bmj.com/site/about/guidelines.xhtml

together with HMIS data permits the calculation of more accurate and precise decentralized prevalence measures by combining HMIS and probability samples at very little cost. In addition to measuring coverage, it also allows us to evaluate the HMIS in practice and point to corrective measures. Such results lead to better systems for tracking the public's health.

ACKNOWLEDGMENTS

We gratefully acknowledge the essential roles of Hemant Das, Alok Prahdan and Sanjay Biswa for their careful field work supporting the LQAS survey and the HMIS data retrieval. We thank Prof Imelda Bates, Prof Brian Faragher and Nancy Vollmer for their valuable feedback on an earlier version of this manuscript.

AUTHORS' CONTRIBUTIONS

JJV, MP developed the research question; MP, CJ led the development of the mathematical statistics for the annealing technique; JV, BD developed the survey design; JV developed the survey methodology; BD managed the survey and data quality; CJ carried out the statistical analyses; JJV obtained the funding and donor support for the research; JJV, MP, CJ interpreted the data; CJ was responsible for data curation; all authors wrote and reviewed the paper.

FUNDING

This research was funded by the Bill and Melinda Gates Foundation Investment ID OPP1142889

DATA SHARING STATEMENT

Household survey data are available upon reasonable request. The recurrent data belong to the Ministry of Health of the State of Bihar and requests for data must be made to them.

For peer review only - http://bmjopen.bmj.com/site/about/guidelines.xhtml

COMPETING INTERESTS

The authors have read the statement on competing interests and declare that they have none.

For peer review only

For peer review only - http://bmjopen.bmj.com/site/about/guidelines.xhtml

REFERENCES

1. Chan M, Kazatchkine M, Lob-Levyt J, et al. Meeting the demand for results and accountability: a call for action on health data from eight global health agencies. *PLoS Med* 2010;7(1):e1000223. doi: 10.1371/journal.pmed.1000223 [published Online First: 2010/02/04]
2. World Health Organization. Sixtieth World Health Assembly: Resolution and Decisions. Geneva: World Health Organisation, 2007.
3. Mavimbe JC, Braa J, Bjune G. Assessing immunization data quality from routine reports in Mozambique. *BMC Public Health* 2005;5:108. doi: 10.1186/1471-2458-5-108
4. Odhiambo-Otieno GW. Evaluation of existing district health management information systems a case study of the district health systems in Kenya. *Int J Med Inform* 2005;74(9):733-44. doi: 10.1016/j.ijmedinf.2005.05.007
5. Aqil A, Lippeveld T, Hozumi D. PRISM framework: a paradigm shift for designing, strengthening and evaluating routine health information systems. *Health Policy Plan* 2009;24(3):217-28. doi: 10.1093/heapol/czp010
6. Mutemwa RI. HMIS and decision-making in Zambia: re-thinking information solutions for district health management in decentralized health systems. *Health Policy Plan* 2006;21(1):40-52. doi: 10.1093/heapol/czj003
7. Cebul RD, Love TE, Jain AK, et al. Electronic health records and quality of diabetes care. *N Engl J Med* 2011;365(9):825-33. doi: 10.1056/NEJMsa1102519
8. Mtema Z, Changalucha J, Cleaveland S, et al. Mobile Phones As Surveillance Tools: Implementing and Evaluating a Large-Scale Intersectoral Surveillance System for Rabies in Tanzania. *PLoS Med* 2016;13(4):e1002002. doi: 10.1371/journal.pmed.1002002
9. Kawonga M, Blaauw D, Fonn S. Aligning vertical interventions to health systems: a case study of the HIV monitoring and evaluation system in South Africa. *Health research policy and systems* 2012;10(1):2.
10. Phaswana-Mafuya N, Peltzer K, Ladzani R, et al. Pre- and post-intervention assessment of a PMTCT-programme-strengthening initiative in a rural area of the Eastern Cape, South Africa. *Afr J AIDS Res* 2011;10(1):83-93. doi: 10.2989/16085906.2011.575551
11. Zulu Z, Kunene S, Mkhonta N, et al. Three parallel information systems for malaria elimination in Swaziland, 2010–2015: are the numbers the same? *Public Health Action* 2018;8(1):S13-S17. doi: 10.5588/pha.17.0058
12. Checchi F, Warsame A, Treacy-Wong V, et al. Public health information in crisis-affected populations: a review of methods and their use for advocacy and action. *Lancet* 2017;390(10109):2297-313. doi: 10.1016/S0140-6736(17)30702-X [published Online First: 2017/06/13]
13. Ronveaux O, Rickert D, Hadler S, et al. The immunization data quality audit: verifying the quality and consistency of immunization monitoring systems. *Bull World Health Organ* 2005;83(7):503-10. [published Online First: 2005/09/24]
14. Admon AJ, Bazile J, Makungwa H, et al. Assessing and improving data quality from community health workers: a successful intervention in Neno, Malawi. *Public Health Action* 2013;3(1):56-59. doi: 10.5588/pha.12.0071 [published Online First: 2013/03/21]
15. Woodworth GF, Baird CJ, Garces-Ambrossi G, et al. Inaccuracy of the administrative database: comparative analysis of two databases for the diagnosis and treatment of intracranial aneurysms. *Neurosurgery* 2009;65(2):251-6; discussion 56-7. doi: 10.1227/01.NEU.0000347003.35690.7A [published Online First: 2009/07/25]
16. Hedt BL, Pagano M. Health indicators: eliminating bias from convenience sampling estimators. *Stat Med* 2011;30(5):560-8. doi: 10.1002/sim.3920
17. Jeffery C, Pagano M, Hemingway J, et al. Hybrid prevalence estimation: Method to improve intervention coverage estimations. *Proc Natl Acad Sci U S A* 2018;115(51):13063-68. doi: 10.1073/pnas.1810287115 [published Online First: 2018/12/07]

For peer review only - http://bmjopen.bmj.com/site/about/guidelines.xhtml

18. Bowley AL. Measurement of the precision attained in sampling. *Bulletin of the International Statistical Institute, 22, Supplement to Liv 1, [*1926;22(Supplement):6-62.
19. Neyman J. On the two different aspects of the representative method: the method of stratified and the method of purposive selection. *Journal of the Royal Statistical Society* 1934;97:558-606.
20. Campbell DT, Stanley J. Quasi Experimental Design. Chicago: Rand McNally 1963.
21. Wilbur CL. Vital statistics for the twelfth United States Census. *Quarterly Publications Journal of the American Statistical Association* 1896-97;5:188-200.
22. Observations et exp6riences concernant des d6nombrements repr6sentatifs. Bulletin of the International Statistical Institute; 1895-6; Berne.
23. Jeffery C, Pagano M, Hemingway J, et al. Hybrid prevalence estimation: a method to improve intervention coverage estimations. *Proceedings of the National Academy of Sciences* In Press
24. Robertson SE, Valadez JJ. Global review of health care surveys using lot quality assurance sampling (LQAS), 1984–2004. *Social Science & Medicine* 2006;63:1648-60.
25. Valadez JJ. Assessing child survival programs in developing countries: testing lot quality assurance sampling. Boston: Harvard University Press 1991.
26. Turner AG, Magnani RJ, Shuaib M. A not quite as quick but much cleaner alternative to the Expanded Programme on Immunization (EPI) Cluster Survey design. *International journal of epidemiology* 1996;25(1):198-203.
27. Davis RH, Valadez JJ. Improving the collection of knowledge, attitude and practice data with community surveys: a comparison of two second-stage sampling methods. *Health Policy Plan* 2014;29(8):1054-60. doi: 10.1093/heapol/czt088
28. Wilbur C. Hadden BD, Hemant Das, Caroline Jeffery, Joseph J. Valadez,. Classical LQAS Compared to CARE LQAS+. Liverpool: Liverpool School of Tropical Medicine, 2017.
29. Valadez JJ, Weiss W, Leburg C, et al. Assessing Community Health Programs: A Trainer's Guide. Using LQAS for Baseline Surveys and Regular Monitoring. St Albans: Teaching-aids At Low Cost 2007.
30. Anoke SC, Mwai P, Jeffery C, et al. Comparing two survey methods of measuring health-related indicators: Lot Quality Assurance Sampling and Demographic Health Surveys. *Trop Med Int Health* 2015;20(12):1756-70. doi: 10.1111/tmi.12605
31. Louis TA. Confidence intervals for a binomial parameter after observing no successes. *The American Statistician* 1981;35(3):154.
32. Rosner B. Fundamentals of Biostatistics. Belmont, CA: Thomson-Brooks/Cole 2006.
33. Chaulagai CN, Moyo CM, Koot J, et al. Design and implementation of a health management information system in Malawi: issues, innovations and results. *Health Policy Plan* 2005;20(6):375-84. doi: 10.1093/heapol/czi044
34. Burton A, Kowalski R, Gacic-Dobo M, et al. A formal representation of the WHO and UNICEF estimates of national immunization coverage: a computational logic approach. *PLoS One* 2012;7(10):e47806. doi: 10.1371/journal.pone.0047806 [published Online First: 2012/11/08]
35. Lim SS, Stein DB, Charrow A, et al. Tracking progress towards universal childhood immunisation and the impact of global initiatives: a systematic analysis of three-dose diphtheria, tetanus, and pertussis immunisation coverage. *Lancet* 2008;372(9655):2031-46. doi: 10.1016/S0140-6736(08)61869-3 [published Online First: 2008/12/17]
36. Boerma T, Eozenou P, Evans D, et al. Monitoring progress towards universal health coverage at country and global levels. *PLoS Med* 2014;11(9):e1001731. doi: 10.1371/journal.pmed.1001731
37. Bruce C. Health management information systems in lower income countries: an analysis of system design, implementation and utilization in Ghana and Nepal. Amsterdam: Royal Tropical Institute, 1997.
38. Lippeveld Theo, Suerborn R, Bodart C. Design and implementation of health information systems. Geneva: World Health Organization 2000.

For peer review only - http://bmjopen.bmj.com/site/about/guidelines.xhtml

39. Lippeveld T, Sauerborn R, Sapirie S. Health information systems--making them work. *World Health Forum* 1997;18(2):176-84.
40. Bethlehem JG. The rise of survey sampling. In: Institute ASsoRted, ed. CBS Discussion Paper: 1572-0314. The Hague: Statistics Netherlands, 2009:28.

For peer review only

For peer review only - http://bmjopen.bmj.com/site/about/guidelines.xhtml

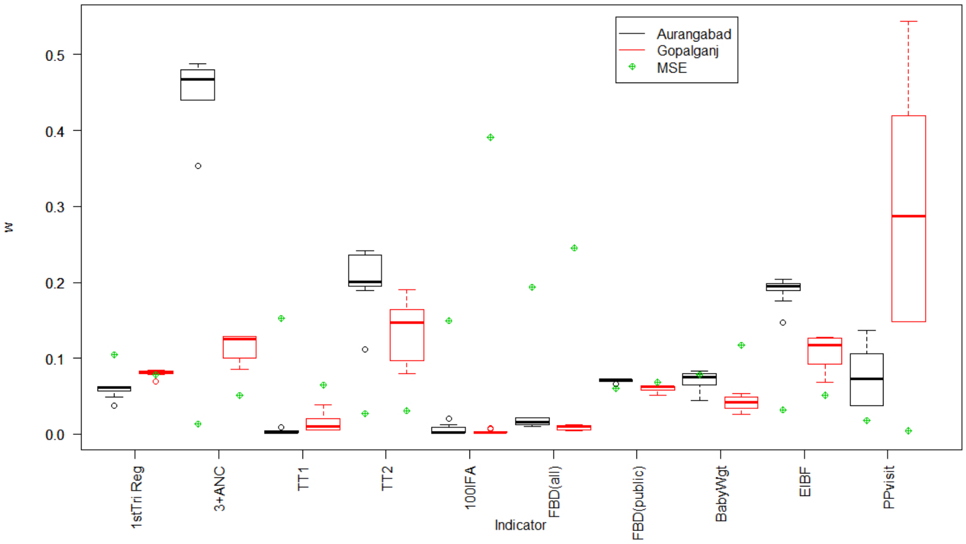

Figure 1: Distribution and average of w for each indicator and district*

407x257mm (72 x 72 DPI)

Block or district	1stTri Reg	3+ANC	TT1	TT2	100IFA	FBD(all)	FBD(public)	BabyWgt	EIBF	PPvisit
Aurangabad Sadar	green	green	blue	green	lightred	blue	blue	blue	blue	green
Barun	green	lightblue	blue	green	red	blue	blue	blue	blue	green
Daudnagar	green	green	blue	lightred	red	blue	green	green	green	green
Deo	green	green	blue	green	red	blue	green	green	green	green
Goh	red	green	blue	lightred	red	lightblue	green	green	green	green
Haspura	green	green	blue	lightred	green	blue	green	green	green	green
Kutumba	red	green	blue	green	red	blue	lightblue	lightblue	green	green
Madanpur	red	green	blue	lightred	red	blue	lightblue	green	lightred	lightred
Nabinagar	red	green	lightblue	green	red	blue	lightblue	green	green	red
Obra	lightred	green	blue	green	red	blue	blue	blue	green	lightred
Rafiganj	red	green	blue	green	red	lightblue	green	lightred	green	lightred
**Aurangabad District Average**	**red**	**green**	**blue**	**lightred**	**red**	**blue**	**blue**	**lightblue**	**lightblue**	**lightred**
Baikunthpur	lightred	lightred	blue	green	red	blue	lightblue	lightblue	green	green
Barauli	red	red	green	lightred	red	blue	lightblue	green	lightblue	green
Bhorey	green	green	lightblue	green	red	blue	green	green	lightblue	lightred
Bijaipur	red	green	lightblue	green	red	blue	blue	blue	blue	lightred
Gopalganj Sadar	red	lightred	green	lightred	red	blue	blue	blue	blue	green
Hathua	lightred	red	blue	lightred	red	blue	green	blue	green	green
Kateya	red	green	green	red	red	blue	lightblue	lightblue	green	green
Kuchaikote	red	red	lightblue	green	red	blue	blue	blue	green	green
Manjha	green	lightblue	lightblue	green	red	blue	blue	blue	lightblue	green
Pach Deuri	green	green	blue	blue	red	blue	lightblue	green	blue	green
Phulwaria	green	green	blue	lightblue	red	blue	green	lightblue	blue	green
Sidhwalia	red	green	blue	green	red	blue	blue	blue	lightblue	green
Thawe	lightred	lightblue	blue	lightred	red	blue	lightblue	blue	blue	green
Uchkagaon	red	green	lightblue	lightred	red	blue	blue	blue	green	lightred
**Gopalganj District Average**	**red**	**lightred**	**blue**	**lightred**	**red**	**blue**	**blue**	**blue**	**blue**	**lightred**

Figure 2: HMIS coverage value compared to the 95% CI of the pcombined estimate for 10 Indicators*

281x148mm (300 x 300 DPI)

Supplementary Information 1

Statistical Annealing Theory

To construct a standard error (SE) for the HMIS coverage estimator, we use the average mean square error (MSE) between the HMIS and LQAS coverage estimators over all the blocks composing one district. The MSE is calculated for the whole district, and can be decomposed in two ways:

$$MSE = \frac{1}{K}\sum_{k=1}^{K}(p_{HMIS\_k} - p_{LQAS\_k})^2 \quad \text{(first decomposition)}$$
$$= \sigma_{HMIS}^2 + \sigma_{LQAS}^2 \quad \text{(second decomposition)}$$

where:

* K is the total number of blocks in the district;
* k is the index for the block in the concerned district, and ranges from 1 to K;
* σ_{HMIS} is the SE of the HMIS coverage p_{HMIS};
* σ_{LQAS} is the SE of the LQAS coverage p_{LQAS}.

The first decomposition measures how much the HMIS and LQAS coverage estimators differ across the blocks composing the district. The second decomposition shows the MSE is also the sum of the two variances of HMIS and LQAS estimators, assuming each source of data was collected independently from each other. We calculate the MSE using the first decomposition and subtract the LQAS variance to obtain the HMIS variance (and thus σ_{HMIS}).

Combined coverage estimator, SE and confidence interval

We define the combined coverage estimator and its SE as a weighted average of the HMIS and LQAS estimators:

$$p_{combined} = w * p_{HMIS} + (1-w) * p_{LQAS}$$
$$\sigma_{combined} = \sqrt{w^2 * \sigma_{HMIS}^2 + (1-w)^2 * \sigma_{LQAS}^2}$$

Where the weighting factor w takes values between 0 and 1:

$$w = \frac{\sigma_{LQAS}^2}{\sigma_{HMIS}^2 + \sigma_{LQAS}^2} = \frac{\sigma_{LQAS}^2}{MSE - \sigma_{LQAS}^2 + \sigma_{LQAS}^2} = \frac{\sigma_{LQAS}^2}{MSE}$$

The 95% CI for the combined coverage estimator is calculated using the standard formula, $p_{combined} \pm 1.96 \times \sigma_{combined}$.

For peer review only

Supplementary Information 2

ANC & Birth preparedness indicators

For the proportion of women who had 3 or more ANC during their pregnancy, the weighting factor ranges from 0.35 to 0.49 in Aurangabad, and from 0.09 to 0.13 in Gopalganj (Table S1). The combined estimate differs from the LQAS estimate by no more than 10%. Standard errors for the HMIS data are 2.8 to 3.4 times larger than those calculated for the combined estimates.

Table S1: Women who had 3 or more ANC visits during their pregnancy.

Block	p_{HMIS}	σ_{HMIS}	p_{lqas}	σ_{lqas}	w	$p_{combined}$	$\sigma_{combined}$	95% CI	σ_{HMIS} / $\sigma_{combined}$
Aurangabad Sadar	0.55	0.08	0.55	0.08	0.48	0.55	0.058	(0.44,0.66)	1.44
Barun	0.48	0.09	0.76	0.07	0.35	0.66	0.055	(0.56,0.77)	1.68
Daudnagar	0.71	0.08	0.53	0.08	0.49	0.62	0.058	(0.5,0.73)	1.43
Deo	0.6	0.08	0.58	0.08	0.48	0.59	0.058	(0.48,0.7)	1.45
Goh	0.67	0.09	0.66	0.08	0.44	0.66	0.058	(0.55,0.78)	1.51
Haspura	0.54	0.08	0.61	0.08	0.47	0.58	0.058	(0.46,0.69)	1.46
Kutumba	0.8	0.09	0.66	0.08	0.44	0.72	0.058	(0.61,0.83)	1.51
Madanpur	0.7	0.09	0.66	0.08	0.44	0.67	0.058	(0.56,0.79)	1.51
Nabinagar	0.62	0.08	0.55	0.08	0.48	0.58	0.058	(0.47,0.7)	1.44
Obra	0.73	0.09	0.66	0.08	0.44	0.69	0.058	(0.58,0.8)	1.51
Rafiganj	0.61	0.08	0.61	0.08	0.47	0.61	0.058	(0.49,0.72)	1.46
**Aurangabad District Average**	**0.64**	**0.03**	**0.62**	**0.02**		**0.63**	**0.018**	**(0.6,0.67)**	**1.49**
Baikunthpur	0.72	0.21	0.53	0.08	0.13	0.55	0.076	(0.4,0.7)	2.79
Barauli	0.77	0.21	0.45	0.08	0.13	0.49	0.075	(0.34,0.64)	2.8
Bhorey	0.7	0.21	0.58	0.08	0.13	0.59	0.075	(0.45,0.74)	2.82
Bijaipur	0.8	0.21	0.74	0.07	0.1	0.74	0.068	(0.61,0.88)	3.17
Gopalganj Sadar	0.8	0.21	0.55	0.08	0.13	0.58	0.075	(0.44,0.73)	2.8
Hathua	0.97	0.21	0.47	0.08	0.13	0.54	0.076	(0.39,0.69)	2.79
Kateya	0.8	0.21	0.74	0.07	0.1	0.74	0.068	(0.61,0.88)	3.17
Kuchaikote	0.9	0.21	0.47	0.08	0.13	0.53	0.076	(0.38,0.68)	2.79
Manjha	0.61	0.22	0.79	0.07	0.09	0.77	0.063	(0.65,0.9)	3.42
Pach Deuri	0.5	0.21	0.47	0.08	0.13	0.48	0.076	(0.33,0.62)	2.79
Phulwaria	0.56	0.21	0.58	0.08	0.13	0.58	0.075	(0.43,0.72)	2.82
Sidhwalia	0.72	0.21	0.66	0.08	0.12	0.67	0.072	(0.52,0.81)	2.94
Thawe	0.62	0.22	0.79	0.07	0.09	0.77	0.063	(0.65,0.9)	3.42
Uchkagaon	0.75	0.22	0.76	0.07	0.09	0.76	0.066	(0.63,0.89)	3.28

For peer review only

**Gopalganj District Average**	**0.75**	**0.06**	**0.59**	**0.02**		**0.61**	**0.021**	**(0.57,0.65)**	**2.91**

For the proportion of women who received TT1 during their pregnancy, the weighting factor ranges from 0.002 to 0.009 in Aurangabad, and from 0.006 to 0.038 in Gopalganj (Table S2). The combined estimate differs from the LQAS estimate by no more than 1%. Standard errors for the HMIS data are 5.1 to 20.2 times larger than those calculated for the combined estimates.

Table S2 Women who received TT1 during their pregnancy.

Block	p_{HMIS}	σ_{HMIS}	p_{lqas}	σ_{lqas}	w	$p_{combined}$	$\sigma_{combined}$	95% CI	σ_{HMIS} / $\sigma_{combined}$
Aurangabad Sadar	0.64	0.39	0.95	0.04	0.01	0.94	0.036	(0.87,1)	10.78
Barun	0.61	0.39	0.97	0.03	0	0.97	0.026	(0.92,1)	15.04
Daudnagar	0.61	0.39	1	0.02	0	1	0.019	(0.96,1)	20.19
Deo	0.59	0.39	0.97	0.03	0	0.97	0.026	(0.92,1)	15.04
Goh	0.54	0.39	0.95	0.04	0.01	0.94	0.036	(0.87,1)	10.78
Haspura	0.59	0.39	1	0.02	0	1	0.019	(0.96,1)	20.19
Kutumba	0.62	0.39	1	0.02	0	1	0.019	(0.96,1)	20.19
Madanpur	0.52	0.39	1	0.02	0	1	0.019	(0.96,1)	20.19
Nabinagar	0.87	0.39	0.97	0.03	0	0.97	0.026	(0.92,1)	15.04
Obra	0.54	0.39	1	0.02	0	1	0.019	(0.96,1)	20.19
Rafiganj	0.54	0.39	1	0.02	0	1	0.019	(0.96,1)	20.19
**Aurangabad District Average**	**0.61**	**0.12**	**0.98**	**0.01**		**0.98**	**0.008**	**(0.97,1)**	**15.6**
Baikunthpur	0.65	0.25	1	0.02	0.01	1	0.019	(0.96,1)	13.15
Barauli	0.95	0.25	0.95	0.04	0.02	0.95	0.036	(0.88,1)	7.02
Bhorey	0.88	0.25	1	0.02	0.01	1	0.019	(0.96,1)	13.15
Bijaipur	0.81	0.25	0.95	0.04	0.02	0.94	0.036	(0.87,1)	7.02
Gopalganj Sadar	0.97	0.25	0.95	0.04	0.02	0.95	0.036	(0.88,1)	7.02
Hathua	0.48	0.25	0.97	0.03	0.01	0.97	0.026	(0.92,1)	9.79
Kateya	0.84	0.25	0.89	0.05	0.04	0.89	0.049	(0.8,0.99)	5.11
Kuchaikote	0.93	0.25	1	0.02	0.01	1	0.019	(0.96,1)	13.15
Manjha	0.75	0.25	0.92	0.04	0.03	0.92	0.043	(0.83,1)	5.81
Pach Deuri	0.6	0.25	1	0.02	0.01	1	0.019	(0.96,1)	13.15
Phulwaria	0.63	0.25	1	0.02	0.01	1	0.019	(0.96,1)	13.15
Sidhwalia	0.68	0.25	0.97	0.03	0.01	0.97	0.026	(0.92,1)	9.79
Thawe	0.72	0.25	1	0.02	0.01	1	0.019	(0.96,1)	13.15
Uchkagaon	0.87	0.25	0.95	0.04	0.02	0.95	0.036	(0.88,1)	7.02

For peer review only

**Gopalganj District Average**	**0.78**	**0.07**	**0.97**	**0.01**		**0.97**	**0.008**	**(0.95,0.98)**	**8.82**

For the proportion of women who received TT2 during their pregnancy, the weighting factor ranges from 0.11 to 0.24 in Aurangabad, and from 0.08 to 0.19 in Gopalganj (Table S3). The combined estimate differs from the LQAS estimate by no more than 7%. Standard errors for the HMIS data are 2 to 3.5 times larger than those calculated for the combined estimates.

Table S3 Women who received TT2 during their pregnancy.

Block	p_{HMIS}	σ_{HMIS}	p_{lqas}	σ_{lqas}	w	$p_{combined}$	$\sigma_{combined}$	95% CI	σ_HMIS / σ_combined
Aurangabad Sadar	0.55	0.14	0.63	0.08	0.23	0.61	0.069	(0.48,0.75)	2.1
Barun	0.8	0.15	0.71	0.07	0.2	0.73	0.066	(0.6,0.86)	2.23
Daudnagar	0.82	0.14	0.61	0.08	0.23	0.66	0.069	(0.52,0.79)	2.07
Deo	0.78	0.15	0.74	0.07	0.19	0.75	0.064	(0.62,0.87)	2.3
Goh	0.8	0.14	0.58	0.08	0.24	0.63	0.07	(0.5,0.77)	2.05
Haspura	0.8	0.14	0.58	0.08	0.24	0.63	0.07	(0.49,0.77)	2.05
Kutumba	0.82	0.15	0.71	0.07	0.2	0.73	0.066	(0.6,0.86)	2.23
Madanpur	0.84	0.14	0.55	0.08	0.24	0.62	0.07	(0.48,0.76)	2.03
Nabinagar	0.79	0.15	0.87	0.05	0.11	0.86	0.052	(0.76,0.96)	2.99
Obra	0.85	0.15	0.74	0.07	0.19	0.76	0.064	(0.63,0.88)	2.3
Rafiganj	0.85	0.15	0.71	0.07	0.2	0.74	0.066	(0.61,0.87)	2.23
**Aurangabad District Average**	**0.8**	**0.05**	**0.68**	**0.02**		**0.71**	**0.02**	**(0.67,0.75)**	**2.26**
Baikunthpur	0.86	0.17	0.87	0.05	0.1	0.87	0.052	(0.77,0.97)	3.22
Barauli	1.04	0.17	0.89	0.05	0.08	0.91	0.048	(0.81,1)	3.54
Bhorey	0.85	0.17	0.87	0.05	0.1	0.87	0.052	(0.76,0.97)	3.22
Bijaipur	0.9	0.17	0.84	0.06	0.11	0.85	0.056	(0.74,0.96)	2.98
Gopalganj Sadar	0.98	0.16	0.71	0.07	0.17	0.76	0.067	(0.63,0.89)	2.4
Hathua	0.93	0.16	0.79	0.07	0.14	0.81	0.061	(0.69,0.93)	2.67
Kateya	1.03	0.16	0.71	0.07	0.17	0.77	0.067	(0.64,0.9)	2.4
Kuchaikote	0.87	0.17	0.89	0.05	0.08	0.89	0.048	(0.8,0.99)	3.54
Manjha	0.87	0.16	0.76	0.07	0.15	0.78	0.063	(0.65,0.9)	2.56
Pach Deuri	0.4	0.16	0.74	0.07	0.16	0.68	0.065	(0.55,0.81)	2.47
Phulwaria	0.7	0.17	0.87	0.05	0.1	0.85	0.052	(0.75,0.95)	3.22
Sidhwalia	0.74	0.16	0.76	0.07	0.15	0.76	0.063	(0.63,0.88)	2.56
Thawe	0.84	0.16	0.66	0.08	0.19	0.69	0.069	(0.56,0.83)	2.29
Uchkagaon	0.92	0.16	0.76	0.07	0.15	0.79	0.063	(0.66,0.91)	2.56

For peer review only

**Gopalganj District Average**	**0.87**	**0.05**	**0.81**	**0.02**		**0.82**	**0.016**	**(0.79,0.85)**	**2.96**

For the proportion of women who received at least 100 IFA tablets during their pregnancy, the weighting factor ranges from 0.002 to 0.02 in Aurangabad, and from 0.001 to 0.01 in Gopalganj (Table S4). The combined estimate differs from the LQAS estimate by no more than 1%. Standard errors for the HMIS data are 7 to 32.3 times larger than those calculated for the combined estimates.

Table S4 Women who received at least 100 IFA tablets during their pregnancy.

Block	p_{HMIS}	σ_{HMIS}	p_{lqas}	σ_{lqas}	w	$p_{combined}$	$\sigma_{combined}$	95% CI	σ_HMIS / $\sigma\_combined$
Aurangabad Sadar	0.13	0.39	0.05	0.04	0.01	0.05	0.036	(0,0.12)	10.68
Barun	0.46	0.39	0.03	0.03	0	0.03	0.026	(0,0.08)	14.9
Daudnagar	0.59	0.39	0	0.02	0	0	0.019	(0,0.04)	20.01
Deo	0.45	0.39	0	0.02	0	0	0.019	(0,0.04)	20.01
Goh	0.18	0.39	0	0.02	0	0	0.019	(0,0.04)	20.01
Haspura	0.16	0.38	0.08	0.04	0.01	0.08	0.043	(0,0.17)	8.85
Kutumba	0.34	0.39	0.05	0.04	0.01	0.06	0.036	(0,0.13)	10.68
Madanpur	0.42	0.39	0	0.02	0	0	0.019	(0,0.04)	20.01
Nabinagar	0.6	0.38	0.13	0.05	0.02	0.14	0.054	(0.03,0.25)	7.06
Obra	0.34	0.39	0	0.02	0	0	0.019	(0,0.04)	20.01
Rafiganj	0.51	0.39	0	0.02	0	0	0.019	(0,0.04)	20.01
**Aurangabad District Average**	**0.39**	**0.12**	**0.03**	**0.01**		**0.04**	**0.01**	**(0.02,0.05)**	**12.02**
Baikunthpur	0.52	0.62	0.03	0.03	0	0.03	0.026	(0,0.08)	24.08
Barauli	0.4	0.62	0	0.02	0	0	0.019	(0,0.04)	32.33
Bhorey	0.79	0.62	0.11	0.05	0.01	0.11	0.05	(0.01,0.21)	12.56
Bijaipur	0.29	0.62	0.03	0.03	0	0.03	0.026	(0,0.08)	24.08
Gopalganj Sadar	0.63	0.62	0.05	0.04	0	0.05	0.036	(0,0.13)	17.26
Hathua	0.86	0.62	0.03	0.03	0	0.03	0.026	(0,0.08)	24.08
Kateya	0.8	0.62	0.03	0.03	0	0.03	0.026	(0,0.08)	24.08
Kuchaikote	0.6	0.62	0.03	0.03	0	0.03	0.026	(0,0.08)	24.08
Manjha	0.36	0.62	0.05	0.04	0	0.05	0.036	(0,0.12)	17.26
Pach Deuri	1.47	0.62	0.13	0.05	0.01	0.14	0.055	(0.03,0.25)	11.4
Phulwaria	0.4	0.62	0.05	0.04	0	0.05	0.036	(0,0.12)	17.26
Sidhwalia	0.38	0.62	0.05	0.04	0	0.05	0.036	(0,0.12)	17.26
Thawe	0.59	0.62	0.05	0.04	0	0.05	0.036	(0,0.13)	17.26
Uchkagaon	0.43	0.62	0	0.02	0	0	0.019	(0,0.04)	32.33

For peer review only

**Gopalganj District Average**	**0.59**	**0.18**	**0.04**	**0.01**		**0.04**	**0.009**	**(0.03,0.06)**	**20.2**

Birth history indicators

For the proportion of infants who had institutional delivery, the weighting factor ranges from 0.01 to 0.02 in Aurangabad, and from 0.005 to 0.01 in Gopalganj (Table S5). The combined estimate differs from the LQAS estimate by no more than 1%. Standard errors for the HMIS data are 6.7 to 14.6 times larger than those calculated for the combined estimates.

Table S5 Institutional Deliveries (all institutions)

Block	p_{HMIS}	σ_{HMIS}	p_{lqas}	σ_{lqas}	w	$p_{combined}$	$\sigma_{combined}$	95% CI	$\sigma\_HMIS / \sigma\_combined$
Aurangabad Sadar	0.03	0.44	0.88	0.04	0.01	0.87	0.043	(0.78,0.95)	10.11
Barun	0.28	0.44	0.82	0.05	0.01	0.82	0.05	(0.72,0.92)	8.73
Daudnagar	0.41	0.44	0.81	0.05	0.01	0.8	0.052	(0.7,0.9)	8.41
Deo	0.38	0.43	0.63	0.06	0.02	0.63	0.063	(0.5,0.75)	6.88
Goh	0.38	0.43	0.6	0.06	0.02	0.59	0.064	(0.47,0.72)	6.77
Haspura	0.51	0.44	0.77	0.06	0.02	0.77	0.055	(0.66,0.88)	7.91
Kutumba	0.41	0.44	0.86	0.05	0.01	0.85	0.046	(0.77,0.94)	9.56
Madanpur	0.4	0.44	0.7	0.06	0.02	0.7	0.06	(0.58,0.81)	7.26
Nabinagar	0.25	0.43	0.63	0.06	0.02	0.62	0.063	(0.5,0.75)	6.88
Obra	0.3	0.44	0.84	0.05	0.01	0.84	0.048	(0.74,0.93)	9.1
Rafiganj	0.38	0.43	0.58	0.07	0.02	0.57	0.065	(0.45,0.7)	6.72
**Aurangabad District Average**	**0.34**	**0.13**	**0.73**	**0.02**		**0.72**	**0.018**	**(0.69,0.76)**	**7.59**
Baikunthpur	0.44	0.49	0.79	0.05	0.01	0.79	0.054	(0.68,0.89)	9.17
Barauli	0.47	0.49	0.84	0.05	0.01	0.84	0.048	(0.74,0.93)	10.25
Bhorey	0.48	0.49	0.81	0.05	0.01	0.8	0.052	(0.7,0.91)	9.47
Bijaipur	0.35	0.49	0.82	0.05	0.01	0.82	0.05	(0.72,0.92)	9.83
Gopalganj Sadar	0	0.49	0.84	0.05	0.01	0.83	0.048	(0.74,0.93)	10.25
Hathua	0.55	0.49	0.91	0.04	0.01	0.91	0.037	(0.84,0.98)	13.21
Kateya	0.48	0.49	0.93	0.03	0	0.93	0.034	(0.86,0.99)	14.63
Kuchaikote	0.34	0.49	0.77	0.06	0.01	0.77	0.055	(0.66,0.87)	8.91
Manjha	0.2	0.49	0.81	0.05	0.01	0.8	0.052	(0.7,0.9)	9.47
Pach Deuri	0.46	0.49	0.77	0.06	0.01	0.77	0.055	(0.66,0.88)	8.91
Phulwaria	0.42	0.49	0.84	0.05	0.01	0.84	0.048	(0.74,0.93)	10.25
Sidhwalia	0.31	0.49	0.81	0.05	0.01	0.8	0.052	(0.7,0.9)	9.47
Thawe	0.33	0.49	0.93	0.03	0	0.93	0.034	(0.86,0.99)	14.63
Uchkagaon	0.32	0.49	0.91	0.04	0.01	0.91	0.037	(0.84,0.98)	13.21

For peer review only

**Gopalganj District Average**	**0.37**	**0.14**	**0.84**	**0.01**		**0.83**	**0.014**	**(0.8,0.86)**	**9.96**

For the proportion of infants who had institutional delivery in a public facility, the weighting factor ranges from 0.066 to 0.073 in Aurangabad, and from 0.05 to 0.06 in Gopalganj (Table S6). The combined estimate differs from the LQAS estimate by no more than 4%. Standard errors for the HMIS data are 3.7 to 4.4 times larger than those calculated for the combined estimates.

Table S6 Public Institutional Deliveries

Block	p_{HMIS}	σ_{HMIS}	p_{lqas}	σ_{lqas}	w	$p_{combined}$	$\sigma_{combined}$	95% CI	$\sigma\_HMIS / \sigma\_combined$
Aurangabad Sadar	0.03	0.24	0.65	0.06	0.07	0.61	0.061	(0.49,0.73)	3.88
Barun	0.28	0.24	0.56	0.07	0.07	0.54	0.063	(0.42,0.67)	3.73
Daudnagar	0.41	0.24	0.51	0.07	0.07	0.5	0.064	(0.38,0.63)	3.7
Deo	0.38	0.24	0.44	0.07	0.07	0.43	0.063	(0.31,0.56)	3.73
Goh	0.38	0.24	0.44	0.07	0.07	0.43	0.063	(0.31,0.56)	3.73
Haspura	0.51	0.24	0.61	0.06	0.07	0.61	0.062	(0.48,0.73)	3.8
Kutumba	0.41	0.24	0.61	0.06	0.07	0.6	0.062	(0.48,0.72)	3.8
Madanpur	0.4	0.24	0.58	0.07	0.07	0.57	0.063	(0.44,0.69)	3.75
Nabinagar	0.25	0.24	0.4	0.06	0.07	0.39	0.063	(0.27,0.52)	3.77
Obra	0.3	0.24	0.56	0.07	0.07	0.54	0.063	(0.42,0.67)	3.73
Rafiganj	0.38	0.24	0.44	0.07	0.07	0.43	0.063	(0.31,0.56)	3.73
**Aurangabad District Average**	**0.34**	**0.07**	**0.52**	**0.02**		**0.51**	**0.019**	**(0.47,0.54)**	**3.76**
Baikunthpur	0.44	0.25	0.58	0.07	0.06	0.57	0.063	(0.45,0.69)	4
Barauli	0.47	0.25	0.61	0.06	0.06	0.61	0.062	(0.48,0.73)	4.06
Bhorey	0.48	0.25	0.56	0.07	0.06	0.56	0.064	(0.43,0.68)	3.98
Bijaipur	0.35	0.25	0.65	0.06	0.06	0.63	0.061	(0.51,0.75)	4.14
Gopalganj Sadar	0	0.25	0.56	0.07	0.06	0.53	0.064	(0.4,0.65)	3.98
Hathua	0.55	0.25	0.65	0.06	0.06	0.64	0.061	(0.52,0.76)	4.14
Kateya	0.48	0.25	0.7	0.06	0.05	0.69	0.059	(0.57,0.81)	4.32
Kuchaikote	0.34	0.25	0.58	0.07	0.06	0.56	0.063	(0.44,0.69)	4
Manjha	0.2	0.25	0.53	0.07	0.06	0.51	0.064	(0.38,0.63)	3.96
Pach Deuri	0.46	0.25	0.6	0.06	0.06	0.59	0.063	(0.46,0.71)	4.03
Phulwaria	0.42	0.25	0.49	0.07	0.06	0.49	0.064	(0.36,0.61)	3.95
Sidhwalia	0.31	0.25	0.72	0.06	0.05	0.7	0.058	(0.58,0.81)	4.4
Thawe	0.33	0.25	0.47	0.07	0.06	0.46	0.064	(0.34,0.59)	3.96
Uchkagaon	0.32	0.25	0.58	0.07	0.06	0.56	0.063	(0.44,0.69)	4

For peer review only

**Gopalganj District Average**	**0.37**	**0.07**	**0.59**	**0.02**		**0.58**	**0.018**	**(0.54,0.61)**	**4.04**

FLW support and Neonatal Health indicators

For the proportion of infants who were weighed during delivery, the weighting factor ranges from 0.04 to 0.08 in Aurangabad, and from 0.03 to 0.05 in Gopalganj (Table S7). The combined estimate differs from the LQAS estimate by no more than 3%. Standard errors for the HMIS data are 3.5 to 6.2 times larger than those calculated for the combined estimates.

Table S7 Infants who were weighed during delivery.

Block	p_{HMIS}	σ_{HMIS}	p_{lqas}	σ_{lqas}	w	$p_{combined}$	$\sigma_{combined}$	95% CI	σ_HMIS / $\sigma\_combined$
Aurangabad Sadar	0.1	0.27	0.84	0.06	0.04	0.81	0.058	(0.7,0.92)	4.73
Barun	0.49	0.27	0.76	0.07	0.06	0.75	0.067	(0.62,0.88)	4.06
Daudnagar	0.52	0.27	0.63	0.08	0.08	0.62	0.075	(0.48,0.77)	3.58
Deo	0.46	0.27	0.58	0.08	0.08	0.57	0.077	(0.42,0.72)	3.5
Goh	0.52	0.27	0.55	0.08	0.08	0.55	0.077	(0.4,0.7)	3.47
Haspura	0.61	0.27	0.63	0.08	0.08	0.63	0.075	(0.48,0.78)	3.58
Kutumba	0.53	0.27	0.71	0.07	0.07	0.7	0.071	(0.56,0.84)	3.8
Madanpur	0.57	0.27	0.71	0.07	0.07	0.7	0.071	(0.56,0.84)	3.8
Nabinagar	0.66	0.27	0.66	0.08	0.08	0.66	0.074	(0.51,0.8)	3.64
Obra	0.46	0.27	0.82	0.06	0.05	0.8	0.061	(0.68,0.92)	4.45
Rafiganj	0.6	0.27	0.42	0.08	0.08	0.44	0.077	(0.29,0.59)	3.5
**Aurangabad District Average**	**0.51**	**0.08**	**0.66**	**0.02**		**0.65**	**0.022**	**(0.61,0.69)**	**3.75**
Baikunthpur	0.58	0.34	0.76	0.07	0.04	0.76	0.068	(0.62,0.89)	4.96
Barauli	0.52	0.33	0.61	0.08	0.05	0.6	0.077	(0.45,0.75)	4.31
Bhorey	0.65	0.33	0.63	0.08	0.05	0.63	0.076	(0.48,0.78)	4.37
Bijaipur	0.48	0.33	0.74	0.07	0.04	0.73	0.07	(0.59,0.86)	4.79
Gopalganj Sadar	0.05	0.33	0.74	0.07	0.04	0.71	0.07	(0.57,0.84)	4.79
Hathua	0.54	0.34	0.87	0.05	0.03	0.86	0.054	(0.75,0.97)	6.24
Kateya	0.6	0.34	0.82	0.06	0.03	0.81	0.062	(0.69,0.93)	5.44
Kuchaikote	0.4	0.34	0.79	0.07	0.04	0.77	0.065	(0.65,0.9)	5.17
Manjha	0.29	0.33	0.68	0.08	0.05	0.67	0.074	(0.52,0.81)	4.54
Pach Deuri	0.56	0.33	0.68	0.08	0.05	0.68	0.074	(0.53,0.82)	4.54
Phulwaria	0.5	0.33	0.66	0.08	0.05	0.65	0.075	(0.5,0.8)	4.45
Sidhwalia	0.37	0.34	0.79	0.07	0.04	0.77	0.065	(0.65,0.9)	5.17
Thawe	0.35	0.34	0.82	0.06	0.03	0.8	0.062	(0.68,0.92)	5.44
Uchkagaon	0.44	0.34	0.84	0.06	0.03	0.83	0.058	(0.72,0.94)	5.78

For peer review only

**Gopalganj District Average**	**0.45**	**0.09**	**0.74**	**0.02**		**0.73**	**0.019**	**(0.7,0.77)**	**4.94**

For the proportion of infants who were breast fed within an hour of their birth, the weighting factor ranges from 0.15 to 0.20 in Aurangabad, and from 0.07 to 0.13 in Gopalganj (Table S8). The combined estimate differs from the LQAS estimate by no more than 8%. Standard errors for the HMIS data are 2.2 to 3.8 times larger than those calculated for the combined estimates.

Table S8 Infants who were breast fed within an hour of their birth.

Block	p_{HMIS}	σ_{HMIS}	p_{lqas}	σ_{lqas}	w	$p_{combined}$	$\sigma_{combined}$	95% CI	σ_HMIS / $\sigma\_combined$
Aurangabad Sadar	0.1	0.16	0.5	0.08	0.2	0.42	0.072	(0.28,0.56)	2.22
Barun	0.49	0.17	0.76	0.07	0.15	0.72	0.064	(0.6,0.85)	2.61
Daudnagar	0.52	0.16	0.42	0.08	0.2	0.44	0.072	(0.3,0.58)	2.24
Deo	0.46	0.16	0.5	0.08	0.2	0.49	0.072	(0.35,0.63)	2.22
Goh	0.52	0.16	0.68	0.08	0.18	0.66	0.068	(0.52,0.79)	2.38
Haspura	0.61	0.16	0.61	0.08	0.19	0.61	0.071	(0.47,0.74)	2.27
Kutumba	0.53	0.16	0.63	0.08	0.19	0.61	0.07	(0.47,0.75)	2.3
Madanpur	0.57	0.16	0.37	0.08	0.19	0.41	0.07	(0.27,0.54)	2.3
Nabinagar	0.66	0.16	0.61	0.08	0.19	0.62	0.071	(0.48,0.76)	2.27
Obra	0.46	0.16	0.63	0.08	0.19	0.6	0.07	(0.46,0.74)	2.3
Rafiganj	0.6	0.16	0.58	0.08	0.2	0.58	0.072	(0.44,0.72)	2.24
**Aurangabad District Average**	**0.51**	**0.05**	**0.58**	**0.02**		**0.57**	**0.022**	**(0.52,0.61)**	**2.3**
Baikunthpur	0.58	0.21	0.66	0.08	0.12	0.65	0.072	(0.51,0.79)	2.94
Barauli	0.52	0.21	0.68	0.08	0.11	0.67	0.071	(0.53,0.81)	3
Bhorey	0.65	0.22	0.84	0.06	0.07	0.83	0.057	(0.72,0.94)	3.83
Bijaipur	0.48	0.22	0.76	0.07	0.09	0.74	0.066	(0.61,0.87)	3.28
Gopalganj Sadar	0.05	0.21	0.53	0.08	0.13	0.47	0.076	(0.32,0.61)	2.79
Hathua	0.58	0.21	0.58	0.08	0.13	0.58	0.075	(0.43,0.73)	2.83
Kateya	0.6	0.21	0.63	0.08	0.12	0.63	0.073	(0.48,0.77)	2.89
Kuchaikote	0.4	0.21	0.55	0.08	0.13	0.53	0.075	(0.39,0.68)	2.81
Manjha	0.3	0.21	0.53	0.08	0.13	0.5	0.076	(0.35,0.65)	2.79
Pach Deuri	0.56	0.22	0.82	0.06	0.08	0.8	0.06	(0.68,0.91)	3.6
Phulwaria	0.5	0.22	0.76	0.07	0.09	0.74	0.066	(0.61,0.87)	3.28
Sidhwalia	0.47	0.21	0.71	0.07	0.11	0.69	0.07	(0.55,0.82)	3.08
Thawe	0.35	0.21	0.63	0.08	0.12	0.6	0.073	(0.45,0.74)	2.89
Uchkagaon	0.44	0.21	0.47	0.08	0.13	0.47	0.076	(0.32,0.62)	2.79

For peer review only

**Gopalganj District Average**	**0.46**	**0.06**	**0.64**	**0.02**		**0.62**	**0.02**	**(0.58,0.66)**	**2.95**

For the proportion of newborns who were visited by any FLW within 24hours of home delivery, the weighting factor ranges from 0.04 to 0.14 in Aurangabad, and from 0.15 to 0.54 in Gopalganj (Table S9). The combined estimate differs from the LQAS estimate by no more than 3%. Standard errors for the HMIS data are 1.4 to 5.2 times larger than those calculated for the combined estimates.

Table S9 Home visit by any FLW within 24 hours of delivery (home deliveries only)

Block	p_{HMIS}	σ_{HMIS}	p_{lqas}	σ_{lqas}	w	$p_{combined}$	$\sigma_{combined}$	95% CI	σ_HMIS / $\sigma\_combined$
Aurangabad Sadar	0.02	0.13	0.03	0.03	0.04	0.03	0.025	(0,0.08)	5.18
Barun	0.16	0.12	0.11	0.05	0.14	0.11	0.046	(0.02,0.2)	2.7
Daudnagar	0	0.13	0.03	0.03	0.04	0.03	0.025	(0,0.08)	5.18
Deo	0.01	0.13	0.08	0.04	0.11	0.07	0.041	(0,0.15)	3.08
Goh	0.03	0.13	0.05	0.04	0.07	0.05	0.035	(0,0.12)	3.71
Haspura	0.1	0.12	0.11	0.05	0.14	0.11	0.046	(0.01,0.2)	2.7
Kutumba	0.08	0.13	0.05	0.04	0.07	0.05	0.035	(0,0.12)	3.71
Madanpur	0.18	0.13	0.05	0.04	0.07	0.06	0.035	(0,0.13)	3.71
Nabinagar	0.4	0.13	0.03	0.03	0.04	0.04	0.025	(0,0.09)	5.18
Obra	0.12	0.13	0.03	0.03	0.04	0.03	0.025	(0,0.08)	5.18
Rafiganj	0.23	0.13	0.08	0.04	0.11	0.1	0.041	(0.01,0.18)	3.08
**Aurangabad District Average**	**0.14**	**0.04**	**0.06**	**0.01**		**0.06**	**0.011**	**(0.04,0.08)**	**3.68**
Baikunthpur	0.09	0.05	0.08	0.04	0.42	0.08	0.033	(0.02,0.15)	1.54
Barauli	0.06	0.05	0.08	0.04	0.42	0.07	0.033	(0,0.14)	1.54
Bhorey	0.18	0.06	0.03	0.03	0.15	0.05	0.024	(0,0.1)	2.6
Bijaipur	0.14	0.06	0.03	0.03	0.15	0.04	0.024	(0,0.09)	2.6
Gopalganj Sadar	0.05	0.06	0.05	0.04	0.29	0.05	0.031	(0,0.11)	1.86
Hathua	0.06	0.05	0.08	0.04	0.42	0.07	0.033	(0,0.13)	1.54
Kateya	0.13	0.06	0.05	0.04	0.29	0.07	0.031	(0.01,0.13)	1.86
Kuchaikote	0.07	0.06	0.03	0.03	0.15	0.03	0.024	(0,0.08)	2.6
Manjha	0.09	0.05	0.11	0.05	0.54	0.1	0.034	(0.03,0.16)	1.36
Pach Deuri	0.11	0.06	0.05	0.04	0.29	0.07	0.031	(0.01,0.13)	1.86
Phulwaria	0.08	0.06	0.05	0.04	0.29	0.06	0.031	(0,0.12)	1.86
Sidhwalia	0.16	0.05	0.08	0.04	0.42	0.11	0.033	(0.05,0.18)	1.54
Thawe	0.03	0.06	0.05	0.04	0.29	0.05	0.031	(0,0.11)	1.86
Uchkagaon	0.12	0.06	0.03	0.03	0.15	0.04	0.024	(0,0.09)	2.6

**Gopalganj District Average**	**0.09**	**0.02**	**0.06**	**0.01**		**0.06**	**0.008**	**(0.05,0.08)**	**1.91**

For peer review only

An innovative approach to improve information accuracy in a two-district cross-sectional study in Bihar, India

Journal:	*BMJ Open*
Manuscript ID	bmjopen-2021-051427.R1
Article Type:	Original research
Date Submitted by the Author:	04-Nov-2021

Complete List of Authors:	Jeffery, Caroline; Liverpool School of Tropical Medicine, Department of International Public Health<br>Pagano, Marcello; Harvard TH Chan School of Public Health, Department of Biostatistics<br>Devkota, Baburam; Liverpool School of Tropical Medicine, Department of International Public Health<br>Valadez, Joseph; Liverpool School of Tropical Medicine, Department of International Public Health

**Primary Subject Heading**:	Global health
Secondary Subject Heading:	Health informatics, Health policy, Health services research, Public health, Evidence based practice
Keywords:	Health policy < HEALTH SERVICES ADMINISTRATION & MANAGEMENT, Public health < INFECTIOUS DISEASES, Health informatics < BIOTECHNOLOGY & BIOINFORMATICS, International health services < HEALTH SERVICES ADMINISTRATION & MANAGEMENT

SCHOLARONE™
Manuscripts

I, the Submitting Author has the right to grant and does grant on behalf of all authors of the Work (as defined in the below author licence), an exclusive licence and/or a non-exclusive licence for contributions from authors who are: i) UK Crown employees; ii) where BMJ has agreed a CC-BY licence shall apply, and/or iii) in accordance with the terms applicable for US Federal Government officers or employees acting as part of their official duties; on a worldwide, perpetual, irrevocable, royalty-free basis to BMJ Publishing Group Ltd ("BMJ") its licensees and where the relevant Journal is co-owned by BMJ to the co-owners of the Journal, to publish the Work in this journal and any other BMJ products and to exploit all rights, as set out in our licence.

The Submitting Author accepts and understands that any supply made under these terms is made by BMJ to the Submitting Author unless you are acting as an employee on behalf of your employer or a postgraduate student of an affiliated institution which is paying any applicable article publishing charge ("APC") for Open Access articles. Where the Submitting Author wishes to make the Work available on an Open Access basis (and intends to pay the relevant APC), the terms of reuse of such Open Access shall be governed by a Creative Commons licence – details of these licences and which Creative Commons licence will apply to this Work are set out in our licence referred to above.

Other than as permitted in any relevant BMJ Author's Self Archiving Policies, I confirm this Work has not been accepted for publication elsewhere, is not being considered for publication elsewhere and does not duplicate material already published. I confirm all authors consent to publication of this Work and authorise the granting of this licence.

For peer review only

An innovative approach to improve information accuracy in a two-district cross-sectional study in Bihar, India

Authors: Caroline Jeffery[1], Marcello Pagano[2], Baburam Devkota[1], Joseph J Valadez[1]

[1] Department of International Public Health, Liverpool School of Tropical Medicine, Pembroke Place, Liverpool, UK

[2] Department of Biostatistics, Harvard T.H. Chan School of Public Health, 677 Huntington Avenue, Boston, MA, USA

Corresponding author: Joseph J Valadez, joseph.valadez@lstmed.ac.uk

For peer review only

For peer review only - http://bmjopen.bmj.com/site/about/guidelines.xhtml

ABSTRACT

OBJECTIVE

Combine Health Management Information Systems (HMIS) and probability survey data using the statistical annealing technique (AT) to produce more accurate health coverage estimates than either source of data and a measure of HMIS data error.

SETTING

This study is set in Bihar, the 5th poorest state in India., where half the population lives below the poverty line. An important source of data, used by health professionals for programme decision-making is routine health facility or HMIS data. Its quality is sometimes poor or unknown, and has no measure of its uncertainty. Using AT we combine district-level HMIS and probability survey data (n=475) for the first time for 10 indicators assessing antenatal care, institutional delivery and neonatal care from 11 blocks of Aurangabad and 14 blocks of Gopalganj districts (N=6,253,965) in Bihar State, India.

PARTICIPANTS

Both districts are rural. Bihar is 82.7% Hindu and 16.9% Islamic.

PRIMARY OUTCOME MEASURES

Survey prevalence measures for 10 indicators, corresponding prevalences using HMIS data, combined prevalences calculated with AT, and Standard Errors for each type of data.

RESULTS

The combined and survey estimates differ by <0.10. The combined and HMIS estimates differ by up to 84.2%, with the HMIS having 1.4 to 32.3 times larger error. Of 20 HMIS versus survey coverage

For peer review only

For peer review only - http://bmjopen.bmj.com/site/about/guidelines.xhtml

estimate comparisons across the two districts only five differed by <0.10. Of 250 sub-district-level comparisons of HMIS versus combined estimates, only 36.4% of the HMIS estimates are within the 95%CI of the combined estimate.

CONCLUSIONS

Our statistical innovation increases the accuracy of information available for local health system decision-making, allows evaluation of indicator accuracy, and increases the accuracy of HMIS estimates. The combined estimates with a measure of error better informs health system professionals about their risks when using HMIS estimates, so they can reduce waste by making better decisions. Our results show that AT is an effective method method ready for additional international assessment while also being used to provide affordable information to improve health services.

STRENGTHS AND LIMITATIONS OF THIS STUDY

- Strength: household survey data were captured as a stratified random sample leading to an efficient use of information.
- Strength: administrative data comprise 100% of the available recurrent information available in the two selected districts.
- Strength: the study population is very large covering a large geographical area, reducing the likelihood that the results are pertinent only to a small group of mothers with children; results may be generalizable.
- Strength: the process for combining probability and administrative data has been assessed using a statistically principled approach prior to use in this study.
- Limitation: the study is confined to two districts of Bihar, India which indicates the need for replicating the study in additional States of India and in other country settings.

For peer review only - http://bmjopen.bmj.com/site/about/guidelines.xhtml

KEY WORDS

HMIS, recurrent information systems, hybrid prevalence, combining data, prevalence, indicators

INTRODUCTION

The 17 Sustainable Development Goals (SDGs) were adopted by all United Nations member states in 2015 as an urgent call for action to end poverty and deprivation by following strategies that improve health and education, reduce inequality and spur economic growth. For national strategies to be effective, they must be grounded in the local context. Good quality data to measure the prevalence of disease conditions, or the population's coverage with health services coverage, is an indispensable resource for programme managers and health policy makers to understand their context. This point is equally true in higher income countries as it is in low and middle-income countries (LMIC). In these latter settings, household surveys are generally considered resource intensive, and being carried out at 3-5 year intervals, are not sufficiently frequency for many decisions. As a result, data generated routinely through the Health Management Information Systems (HMIS) is more frequently used for decision-making and for annual reviews than household surveys. HMIS data consists of routine information reported from the health facilities to districts health offices, who submit it to Ministry of Health on a monthly, quarterly, semi-annual and annual basis. A key shortcoming of HMIS data is that it is only representative of those who can access health services. This shortcoming is especially problematic in LMIC where access to healthcare services is constrained by geographic, economic and social determinants, and in settings where there are competing services in the community that do not report into the HMIS.

Demand by global donors for timely and reliable data continues to increase[1]. The Sixtieth World Health Assembly underscored the importance of robust information to strengthen health systems and policies (resolution WHA60.27)[2], and to this end, established a Health Metrics Network (HMN) partnership to aid countries improve data quality of routine information systems to enable their use for health planning and decision-making[3-6]. This was followed by other resolutions and in 2015 by a

For peer review only - http://bmjopen.bmj.com/site/about/guidelines.xhtml

high-level summit on Measurement and Accountability for Results leading to the formation of the Health Data Collaborative (HDC) in March 2016 and supported by global partners. HDC's mandate is more extensive than that of HMN, having an ultimate objective of aiding countries to improve the quality and availability of health data and their ability to consistently and accurately report on progress towards the health-related Sustainable Development Goals.

The advent of electronic health records was heralded, as studies showed their association with improved clinical care, outcomes[7], and surveillance[8]. Consequently, the advancement of a computerised District Health Information System-2 (DHIS2) was implemented in 73+ countries to integrate data sources for their more rapid analyses, dissemination and use. An evaluation in Tanzania showed it improved timeliness and completeness of reporting[8]. South Africa established a dedicated HIV/AIDS information system recommending the use of the earlier DHIS for integrating different information sources[9] and as input for designing national program strategies[10]. A 2010-2015 study in Swaziland recommended embracing electronic medical data systems to reduce the discrepancies occurring between the existing three information systems put in place for malaria elimination[11]. Recently, a similar demand was issued by practitioners working in humanitarian settings for an *epidemiology and demography service* to develop robust and timely information from multiple sources to establish priorities that address the population's needs[12].

One important use of routine information systems is to monitor a population's coverage with health service, as Global Alliance on Vaccines and Immunisations (GAVI) has done for vaccination rates since the 1990s. They attempt to control for known weaknesses in administrative data by carrying out data quality audits by recounting, and contrasting, the number vaccinated in health centres with the number originally reported[13].

Although measurement improves all facets of health management, HMIS related studies mainly focus on improvements in obtaining numerators[1 5 14 15]. The future also demands accurate and precise denominators, and, more importantly, a principled method for assessing their accuracy (e.g.,

For peer review only - http://bmjopen.bmj.com/site/about/guidelines.xhtml

standard errors (SE)) so users understand their risk when using the data. Under normal circumstances, the error present in HMIS remains unknown.

The research question we investigate in this paper: How can we measure the error in the HMIS and improve its accuracy? We address this issue by presenting an innovation called *Statistical Annealing* (Annealing Technique or AT). It builds on our earlier pioneering work in 2011[16] and refined in 2018 [17] that provides a coverage estimator with a 95% Confidence Interval (CI) by combining data from a probability survey and HMIS data. HMIS coverage estimates have not before had a 95% Confidence Interval (CI) to inform users of their accuracy. We demonstrated the magnitude of HMIS error and how it can be improved by using AT.

By the early 20th century Bowley[18] and Neyman[19] proved the importance of sampling and the estimated magnitudes of their errors. The standard error (SE)[20] is a commonly used measure of error, which is an estimate of deviation of the sample mean from the actual population mean. The SE is used in calculating a 95%CI, which relates to the accuracy of the estimate. If survey data are combined (annealed) with HMIS appropriately they produce a single, more accurate coverage estimator[16]. However, concurrent well-designed surveys have been a surprisingly neglected sources of information for improving HMIS [21 22]. Recent efforts have used surveys to improve HMIS estimates[23 24], although the resulting revised estimates do not have a corresponding 95%CI.

The objective of AT is to produce a principled measure of health system performance by combining existing HMIS and survey information which also produces an accompanying measure of its accuracy. This hybrid estimate is more accurate than either data source alone. Our proof of principle study, used Child Health Day (CHD) administrative data from Benin and Madagascar provided by UNICEF country offices and household survey data collected at the same time, to verify CHD coverage and the quality of CHD administrative data[17]. This refined approach to AT resulted in the production of a 95% CI for the administrative data and for the combined result. See Supplementary Information 1 for the AT formulae.

For peer review only - http://bmjopen.bmj.com/site/about/guidelines.xhtml

The current study assesses the transferability of AT to a new setting, HMIS data from Bihar state located in northeast India. Bihar is the 5th poorest state of India where half the population lives below the poverty line. It is one of the most densely populated states (N = 110million), and has some of the weakest maternal and child health and nutrition indicators in India [25 26]. Both districts are primarily rural with a mix of upper caste and lower caste Hindu residents, and minority Muslim communities.

Also, this study is the first use of AT with true HMIS data, which in the future will be a more typical use for AT, and result in our conclusions about its global applicability. We apply AT to 10 indicators related to antenatal care (ANC), institutional delivery, and neonatal care using data from two districts, Aurangabad (N=3,695,928) and Gopalganj (N=2,558,037), and two sources of data collected for purposes other than this study. HMIS data from the two districts and a probability survey data collected as part of an earlier assessment of maternal and child health care coverage in Bihar [27]. This secondary data analysis uses datasets without personal identifiers.

METHODS

HMIS system in Bihar

The HMIS data in Bihar consists of 318 data elements. These are reported monthly by health sub-centres (HSC) and aggregated at the block primary health centre (BPHC). The *block* is the sub-district administrative unit in India. Staff at the HSCs tally information from paper registers and send a paper report to BPHCs where it is entered into a computerized HMIS system, and subsequently maintained electronically. District-level indicators comprise information aggregated across the block as well as data from district and referral hospitals.

For peer review only - http://bmjopen.bmj.com/site/about/guidelines.xhtml

Data

To develop AT in Bihar, we used the health facility-based HMIS and probability data from a household survey in two districts, Aurangabad and Gopalganj. From the Bihar State Health Society, we obtained two sources of HMIS data: the HMIS data reported by all health facilities in the two districts, at district level and disaggregated to the block-level; and the Expected Level of Achievement (ELA), which is projected data. They calculated the ELA data using the latest 2011 census and applied an average annual population growth rate using parameter values reported in the Annual Health Survey 2012-13 (e.g., crude birth rate) and in the HMIS data from previous years (e.g., percentage of pregnancies leading to a still birth in 2015-16). We averaged these two data sources, the HMIS and the ELA, to obtain the administrative estimate.

A household probability survey was conducted during 7-25 September 2016 in each district in a standardised manner[28 29] using Lot Quality Assurance Survey (LQAS). The LQAS survey is a stratified random sample used to assess health service coverage. The strata are administrative blocks (11 in Aurangabad, 14 in Gopalganj), which are sub-district units in India. The strata were weighted by their population size. The first stage sample uses probability proportional to size to randomly sample villages (typically, n=19 per stratum[28]). In each random location a second stage sample identifies an index household using segmentation sampling[30 31] from locally constructed hand-drawn maps. The next closest household is then selected for interview to reduce the likelihood that households not included in the map do not have zero probability of selection. In the selected household, individuals in three target groups (women with children 0-2, 3-5 and 0-5 months of age[32]) are listed with one selected randomly using a random number table. The remaining target group is selected in the next closest house using the same protocol[33]. The resulting sample of individuals is random and provides its own standard error (SE) estimator used for calculating a 95% CI. One member of each target group only was selected in a sampled village.

For peer review only - http://bmjopen.bmj.com/site/about/guidelines.xhtml

The total number of random samples for each indicator is 209 (Aurangabad) and 266 (Gopalganj). LQAS gives reliable estimates for indicators at the district level[34] and allows the classification of blocks, as "performing adequately" or not. However, in this paper we do not use the data for classification, but rather for estimation.

Indicators for annealing

We based the selection of 10 indicators for annealing on the ease of combining HMIS data temporally to the survey data (Table 1). For the LQAS survey, we measured antenatal care (ANC), institutional delivery, and neonatal care outcomes in the population of women with children 0-2, 3-5 and 0-5 months of age[32] resulting in a total of 627 (Aurangabad) and 798 (Gopalganj) interviews. We used the HMIS data from the last 5-months of reports (April-August 2016) preceding the LQAS survey (Table 1), so that they matched temporally.

The Annealing Technique design

As in earlier work [35], we apply AT in each sub-district for each indicator. The district-level estimators are an aggregation of appropriate block-level coverage data, weighted by the population sizes. Data analysis was done using Stata SE ver15 and R ver-3.4.1.

Survey coverage estimator

For all but two indicators, the numerator of the LQAS coverage is the sum of correct responses in each of two target group (mothers of children 0-2 months and 0-5 months) in each block. The denominator is the sum of the denominators of both groups $(19 \times 2 = 38)$. For the two indicators we used additional data from a third target group, mothers of children 3-5 months, where the denominator is the sum of the denominators of each target group $(19 \times 3 = 57)$.

A step in the annealing process requires a weighted average of component parts; the LQAS estimator is one such component. For each component, the weight used is inversely proportional to its

For peer review only - http://bmjopen.bmj.com/site/about/guidelines.xhtml

For peer review only

variance. If the LQAS coverage, p, is away from the extremes of zero or one, we can use the standard binomial formula, $p(1-p)/n$. When the LQAS coverage is zero (or one), we calculate the weight by using a surrogate `standard error' from a 95% CI using Louis' approach[36] and dividing its length ($1-0.05^{(1/n)}$) by (2×1.96), and squaring the resultant.

To assess any block-level clustering effect between target groups sampled in parallel, we use the two-tail McNemar's test for correlated proportions for each indicator[37], across all blocks sampled in each district.

HMIS coverage estimator

The numerator of the HMIS coverage is reported in Table 1 (Column 4). The denominator is the average between the total number of ANC registration per month calculated by the ELA projected data and those reported in the HMIS data (averaged over April-August 2016).

For peer review only - http://bmjopen.bmj.com/site/about/guidelines.xhtml

Table 1: List of 10 indicators selected for applying the annealing technique in Bihar State, India

Generic name of Indicator	LQAS Indicator	Targeted groups (LQAS surveys)	HMIS indicator	Targeted months (HMIS reported data)
First trimester registration	Proportion of mothers of 0-2 months old whose pregnancy was registered in the first trimester in the last pregnancy	0-2 months<br>0-5 months	Number of pregnant women registered within first trimester	April-August 2016
3 or more ANC	Proportion of mothers of 0-2 months old who had 3 or more ANC's during their entire period of last pregnancy	0-2 months<br>0-5 months	Number of pregnant women received 3 ANC check-ups during pregnancy	April-August 2016
Received TT1	Proportion of mothers of 0-2 months who received TT1 during their last pregnancy	0-2 months<br>0-5 months	Number of pregnant women given TT1 during current pregnancy	April-August 2016
Received TT2	Proportion of mothers of 0-2 months who received TT2 during their last pregnancy	0-2 months<br>0-5 months	Number of pregnant women given TT2 or booster during current pregnancy	April-August 2016
Received 100 IFA tablets	Proportion of mothers of 0-2 months who received at least 100 IFA tablets during their last pregnancy	0-2 months<br>0-5 months	Total number of pregnant women given 100 IFA tablets	April-August 2016
Institutional deliveries (all)	Proportion of 0-3 months infants who had institutional delivery (survey conducted September 2016)	0-2 months<br>3-5 months<br>0-5 months	Total number of deliveries conducted in institutional setting (private and public) including C sections	April-August 2016

For peer review only

Public Institutional Deliveries	Proportion of 0-3 months infants who had institutional delivery at public facility (survey conducted September 2016)	0-2 months<br>3-5 months<br>0-5 months	Number of deliveries conducted in public institutions including C sections	April-August 2016
Baby weighed during delivery	Proportion of 0-2 months infants who were weighed during delivery	0-2 months<br>0-5 months	Number of new-borns weighed at birth	April-August 2016
Early Initiation of Breast feeding (EIBF)	Proportion of 0-2 months infants who were breast fed within an hour of their birth	0-2 months<br>0-5 months	Number of new-borns breast fed within one hour of birth	April-August 2016
Post-Partum home visit within 24 hours (home-deliveries only)	Home visit by any FLW within 24 hours of delivery	0-2 months<br>0-5 months	Number of new-borns visited within 24 hours of delivery for deliveries conducted at home.	April-August 2016

For peer review only - http://bmjopen.bmj.com/site/about/guidelines.xhtml

To calculate the weight, as we did for the LQAS estimator, to use for the HMIS estimator, we use the fact that both estimators are estimating the same quantity, so their difference should be estimating zero. This can then be used to calculate the weight, as we show in [35] and in the Supplementary Information 2 (Tables S1-S9).

Assessing variation in local-level data quality

To advance the use of AT in multiple settings we developed a tool for sub-district-level analysis. We assess the variation in the quality of the HMIS indicators in 250 block-level comparisons of the 10 indicators $(10 \times (11\ Aurangabad + 14\ Gopalganj))$ and 20 district-level comparisons. We classify the value of the HMIS coverage relative to the CI around the combined estimate. If the HMIS coverage is within 95% CI of $p_{combined}$ the cell is coloured green. If the HMIS estimate is within a reasonable percentage difference of each boundary of the CI, for example 10% (we colour those cells light red if it is above the high boundary or light blue if it is below the lower boundary), we are assured its value is not far from the survey estimate. While when the HMIS coverage is beyond the reasonable percentage difference of each boundary, the discrepancy might be too large to recommend combining the two estimates and to not use the HMIS estimate (we colour these dark red or dark blue).

Ethics Statement

The survey data were collected originally for assessment of a maternal and child health care programme in Bihar. The survey protocol was approved in 2016 by the Indian Institute of Public Health Bhubaneswar Institutional Ethics Committee (IIPHB-IEC-2016/010) and the Ethical Review Board of the Liverpool School of Tropical Medicine (research protocol 16-023RS). We obtained oral rather than written informed consent from all respondents, because of the high illiteracy rate. The survey data we later treated as secondary data for the current study which was supported by our

For peer review only - http://bmjopen.bmj.com/site/about/guidelines.xhtml

donor and the Bureau of Statistics for the Ministry of Health for the State of Bihar. After giving permission to access the State HMIS, they facilitated the collection of the HMIS data.

Patient and Public Involvement

This study does not involve patients. Also, the public were not involved in the design, conduct and reporting of the research. The public was engaged as interviewees. To ensure local engagement all data capture was carried out in close coordination with the State Ministry of Health of Bihar. We also shared the results with them and offered further dissemination of results, and engaged them for data use and action planning activities.

RESULTS

Data characteristics and AT characteristics

Among the 10 indicators, the Mean Squared Error (MSE) between the two sources of data ranges from 0.013 to 0.193 in Aurangabad, and between 0.005 and 0.391 in Gopalganj (See Table 2 for the indicators). Three blocks in Gopalganj have an HMIS coverage larger than 100% (two or more tetanus toxoid vaccinations (TT2): 104% in Barauli and 103% in Kateya; iron folic acid (IFA) tablets distribution: 147% in Pach Deuri). The HMIS coverage averaged over blocks ranges between 12% and 80% in Aurangabad, and between 10% and 84% in Gopalganj. The LQAS coverage averaged over the blocks ranges between 3% and 98% in Aurangabad, and between 5% and 97% in Gopalganj. The McNemar tests did not detect block-level clustering except for the four tests with indicators measuring institutional delivery in all facilities or in public facilities.

For peer review only - http://bmjopen.bmj.com/site/about/guidelines.xhtml

Table 2: Summary information before application of AT

District	Indicator	MSE	Average value of p_{HMIS} over all blocks†	Average value of p_{LQAS} over all blocks†	McNemar test p-values ‡
Aurangabad	Pregnancy registered during first 3 months	0.105	0.66	0.42	0.47
	3 or more ANC	0.013	0.64	0.62	0.46
	Mothers who were protected against tetanus during pregnancy (Neonatal TT)	0.152	0.61	0.98	0.71
	Mothers who were protected against tetanus TT2	0.027	0.79	0.67	0.68
	Mothers who received iron folic acid for 100 days or more when they were pregnant	0.150	0.38	0.03	0.71
	Institutional delivery (Both private and public facility)	0.193	0.34	0.74	0-2 vs 3-5mo: 0.73<br>0-2 vs 0-5mo: 0.39<br>0-5 vs 3-5mo: 0.20
	Institutional delivery (public facility)	0.060	0.34	0.53	0-2 vs 3-5mo: 0.05<br>0-2 vs 0-5mo: 0.84<br>0-5 vs 3-5mo: 0.04[§]
	Baby Weighed within one hour of birth	0.078	0.50	0.67	0.65
	Early Initiation of Breast feeding (EIBF)	0.032	0.50	0.57	0.49
	Home visit by any FLW within 24 hours of delivery	0.018	0.12	0.06	0.18
Gopalganj	Pregnancy registered during first 3 months	0.078	0.72	0.48	0.86
	3 or more ANC	0.051	0.73	0.63	0.37

For peer review only - http://bmjopen.bmj.com/site/about/guidelines.xhtml

For peer review only

	Mothers who were protected against tetanus during pregnancy (Neonatal TT)	0.065	0.76	0.97	0.81
	Mothers who were protected against tetanus TT2 *	0.031	0.84	0.78	0.91
	Mothers who received iron folic acid for 100 days or more when they were pregnant *	0.391	0.63	0.05	0.20
	Institutional delivery (Both private and public facility)	0.245	0.35	0.85	0-2 vs 3-5mo: 0.04§<br>0-2 vs 0-5mo: 0.06<br>0-5 vs 3-5mo: 0.89
	Institutional delivery (public facility)	0.068	0.35	0.59	0-2 vs 3-5mo: 0.01§<br>0-2 vs 0-5mo: 0.03§<br>0-5 vs 3-5mo: 0.51
	Baby Weighed within one hour of birth	0.117	0.44	0.75	0.05
	Early Initiation of Breast feeding (EIBF)	0.051	0.45	0.65	0.17
	Home visit by any FLW within 24 hours of delivery	0.005	0.10	0.05	0.45

* Three blocks in Gopalganj have an HMIS coverage larger than 100% (TT2 indicator: 104% in Barauli and 103% in Kateya; IFA tablets indicator: 147% in Pach Deuri)

† 11 blocks in Aurangabad and 14 blocks in Gopalganj.

‡ McNemar test comparing 0-2 vs 0-5 months unless otherwise specified.

§ The McNemar test reports a major difference at p <0.05.

For peer review only - http://bmjopen.bmj.com/site/about/guidelines.xhtml

For peer review only

The weighting factor used in the calculation of the combined estimator varies for each indicator in each district (Figure 1). For six indicators (registration during first trimester of pregnancy, receiving TT1, receiving ≥100 IFA tablets, institutional delivery in any facility, deliveries in public facilities, baby weighed within 1 hour of birth), all block-level weighting factors *w*, are below 0.10 in both districts, indicating the contribution of HMIS estimator to the combined estimator is no higher than 10% (Table 3 and Supplementary Information 2 Tables S1-S9). We use a 1-tail test to check for difference of the weights between the two districts, based on the observed mean difference. The weights for Gopalganj are higher than Aurangabad's for three indicators: first trimester registration, receiving TT1 and having a post-partum visit – for all three, one-sided t-test p-values are <0.01. The Gopalganj weighting factors are lower than Aurangabad's for the other seven indicators: ≥3 ANC visits, receiving TT2, receiving ≥100 IFA tablets, institutional delivery in any facility, deliveries in public facilities, early initiation of breastfeeding (EIBF), baby weighed at delivery (all seven one-sided t-test p-values <0.05). This result indicates that there are more discrepancies between the HMIS estimates and the LQAS estimates in Aurangabad when compared to Gopalganj for the first three indicators, while the discrepancies are greater in Gopalganj for the latter seven indicators. All but one of the weights for the indicator ≥3 ANC visits in Aurangabad are between 0.4 and 0.5, indicating an almost equal contribution of the HMIS and LQAS estimators to the resulting combined estimator. Alternatively, there are large variations in the weights for the indicator measuring the one visit by any Frontline Worker (FLW) within 24-hours of delivery in Gopalganj; while, the MSE is small in this instance.

(Insert Figure 1 here)

[Footnote and Title for Figure 1: *Circles with no filling indicate outlier values

Figure 1: Distribution and average of *w* for each indicator and district*]

For peer review only - http://bmjopen.bmj.com/site/about/guidelines.xhtml

Table 3: Women who registered for ANC during the first three months of pregnancy

Block	p_{HMIS}	σ_{HMIS}	p_{lqas}	σ_{lqas}	w	$p_{combined}$	$\sigma_{combined}$	95% CI	$\sigma_{HMIS}/\ \sigma_{com}$
Aurangabad Sadar	0.42	0.31	0.39	0.08	0.06	0.4	0.077	(0.25,0.55)	4.08
Barun	0.55	0.31	0.58	0.08	0.06	0.58	0.078	(0.43,0.73)	4.04
Daudnagar	0.67	0.31	0.53	0.08	0.06	0.54	0.078	(0.38,0.69)	3.99
Deo	0.65	0.31	0.55	0.08	0.06	0.56	0.078	(0.41,0.71)	4.01
Goh	0.75	0.32	0.18	0.06	0.04	0.21	0.062	(0.08,0.33)	5.14
Haspura	0.46	0.31	0.55	0.08	0.06	0.55	0.078	(0.39,0.70)	4.01
Kutumba	0.78	0.31	0.42	0.08	0.06	0.44	0.078	(0.29,0.60)	4.04
Madanpur	0.74	0.31	0.32	0.08	0.05	0.34	0.073	(0.20,0.48)	4.29
Nabinagar	0.81	0.31	0.37	0.08	0.06	0.39	0.076	(0.25,0.54)	4.13
Obra	0.68	0.31	0.5	0.08	0.06	0.51	0.079	(0.36,0.67)	3.99
Rafiganj	0.77	0.32	0.26	0.07	0.05	0.29	0.070	(0.15,0.42)	4.53
**Aurangabad District Average**	**0.68**	**0.1**	**0.41**	**0.02**		**0.42**	**0.023**	**(0.38,0.47)**	**4.23**
Baikunthpur	0.73	0.27	0.53	0.08	0.08	0.54	0.078	(0.39,0.70)	3.46
Barauli	0.74	0.27	0.39	0.08	0.08	0.42	0.076	(0.27,0.57)	3.53
Bhorey	0.66	0.27	0.53	0.08	0.08	0.54	0.078	(0.39,0.69)	3.46
Bijaipur	0.81	0.27	0.47	0.08	0.08	0.5	0.078	(0.35,0.65)	3.46
Gopalganj Sadar	0.67	0.27	0.37	0.08	0.08	0.39	0.075	(0.24,0.54)	3.58
Hathua	0.94	0.27	0.71	0.07	0.07	0.73	0.071	(0.59,0.87)	3.81
Kateya	0.89	0.27	0.39	0.08	0.08	0.43	0.076	(0.29,0.58)	3.53
Kuchaikote	0.74	0.27	0.39	0.08	0.08	0.42	0.076	(0.27,0.57)	3.53
Manjha	0.67	0.27	0.61	0.08	0.08	0.61	0.076	(0.46,0.76)	3.53
Pach Deuri	0.49	0.27	0.47	0.08	0.08	0.47	0.078	(0.32,0.63)	3.46
Phulwaria	0.61	0.27	0.5	0.08	0.08	0.51	0.078	(0.36,0.66)	3.45
Sidhwalia	0.79	0.27	0.42	0.08	0.08	0.45	0.077	(0.30,0.60)	3.5
Thawe	0.67	0.27	0.42	0.08	0.08	0.44	0.077	(0.29,0.59)	3.5
Uchkagaon	0.71	0.27	0.42	0.08	0.08	0.44	0.077	(0.29,0.59)	3.5
**Gopalganj District Average**	**0.73**	**0.08**	**0.48**	**0.02**		**0.5**	**0.022**	**(0.46,0.54)**	**3.53**

Comparing indicator estimates of 20 p_{HMIS} versus p_{LQAS} across the two districts, five differed by $\leq$0.10 and six others by $\leq$0.20. The remaining nine indicator estimates show larger differences (Table 3; Tables S1-S9).

For peer review only

ANC services

All estimates, SEs and CIs are calculated at the block and district levels (as an illustration, see the detailed results for first trimester ANC registration in Table 3; Supplementary Information 2 Tables S1-S4 for other indicators). For the five indicators measuring coverage related to ANC and birth preparedness, the combined block-level estimate differs from the LQAS estimate by 1% (received ≥100 IFA tablets) to 10% (≥3 ANC visits); the block-level combined estimate differs from the HMIS estimate at most by 28% (TT1) to 133% (received ≥100 IFA tablets). SEs for the HMIS data are 1.4 to 32.3 times larger than those calculated for the combined estimates.

Institutional delivery

For the two indicators measuring the proportion of infants institutionally delivered (Tables S5-S6), the combined block-level estimate differs from the LQAS estimate at most by 1% (all facilities) and 4% (public facilities only); the combined estimate differs from the HMIS estimate at most by 84% (all facilities) and 58% (public facilities only). SEs for the HMIS data are 3.7 to 14.6 times larger than those calculated for the combined estimates.

Neonatal health

For the three indicators measuring coverages related to neonatal health (Supplementary Information 2 Tables S7-S9), the combined block-level estimate differs from the LQAS estimate at most by 3% (newborns who were visited by any FLW within 24-hours of home delivery) to 8% (EIBF); the combined estimate differs from the HMIS estimate at most by 36% (newborns who were visited by any FLW within 24-hours of home delivery) to 70% (EIBF). SEs for the HMIS data are 1.4 to 6.2 times larger than those calculated for the combined estimates.

For peer review only - http://bmjopen.bmj.com/site/about/guidelines.xhtml

Local level variation

We first look at the two rows of Figure 2 that summarize the district level measures and see that, on average, Aurangabad's HMIS measures (row 13) behave slightly better than Gopalganj's (row 28). We see that for two indicators both districts are red; for three they are both blue; for two they are both light red; and for two Aurangabad's are light blue versus Gopalganj's dark blue; and, for one Aurangabad's is green and Gopalganj's is light red.

(Insert Figure 2 here)

[Footnote and Title for Figure 2:

*5 categories: (Blue) HMIS coverage is more than 10% below the lower bound. (Light blue) HMIS coverage is less than 10% below the CI lower bound. (Green) HMIS coverage is within the CI. (Light red) HMIS coverage is less than 10% above the CI upper bound, (Red) HMIS coverage is more than 10% above the CI upper bound.

Figure 2: HMIS coverage value compared to the 95% CI of the $p_{combined}$ estimate for 10 Indicators*]

We get a more detailed contrast when investigating the block-level results. Of the 250 comparisons 36.4% of the HMIS estimates are within the CI of the combined estimate with Aurangabad displaying greater accuracy (44.5%) than Gopalganj (30%). For two indicators (100 IFA tablets received and facility based delivery (all)) the results are consistently poor across blocks. The same can be said of TT1 in Aurangabad, but not in Gopalganj. These indicators warrant further investigation into why the two sources of data have such discrepancies. 3+ANC displays good agreement in Aurangabad, but a mixed evaluation in Gopalganj. Similarly, with the remaining indicators, we can detect subtly different results within the two districts. These results suggest substantial variation across blocks, districts and indicators in the HMIS accuracy.

For peer review only - http://bmjopen.bmj.com/site/about/guidelines.xhtml

DISCUSSION

The results of our study show numerous discrepancies of block-level HMIS coverage estimates in both districts compared to the probability samples. However, the extent of these discrepancies varies across the blocks. We would expect consistency among block-level results given their geographic and political proximity within these two districts. Using the combined estimates as our guides, they differ from the respective probability estimates by at most 10%. In contrast, the combined estimates differ from the HMIS estimates by up to 84.2% (FBD (all) in Aurangabad's block Sadar), except for one instance where the difference is 133% due to the administrative coverage being >>100% (distribution IFA during pregnancy in Pach Deuri). SEs for the HMIS estimates are between 1.4 and 32.3 times larger than for the combined estimates (Supplementary Information 2 Tables S1, S9 and S4).

The HMIS data provides two sources of information to calculate the denominator (reported HMIS and Expected Level of Achievement-ELA). Across the 14 blocks of Gopalganj, the two values were very close. In Aurangabad we observe a linear relationship between the two sets of values, with the ELA values being slightly higher than the HMIS recorded values, suggesting the projected data overestimates the numbers reported. Since there was no evidence that one was better than the other, we chose to take the average of both values to define the denominator of the HMIS estimator. This step can be used in future applications.

A general attractiveness of the HMIS results for management purposes is their results apply at the block-level, and we do not wish to lose this property. We are fortunate because this level also corresponds to the strata in the LQAS surveys, and thus, this is the information level provided by the combined estimates. For greater granularity, LQAS is a favourable method to use for AT. In this study, we were also able to increase the sample size by pooling the data from two or three target populations surveyed in parallel within a block.

For peer review only - http://bmjopen.bmj.com/site/about/guidelines.xhtml

Our approach allows us to contrast the two districts of Bihar by studying the block-level (sub-district-level) weighting factors *w*. For example, six indicators across both districts, have weights lower than 0.10, highlighting a large standard error in the HMIS estimates versus those in the probability sample estimates which leads to a wide 95%CI in the HMIS estimates. The contribution of the HMIS estimator to the combined estimator for the indicator measuring visits by any Frontline Worker (FLW) within 24-hours of birth estimator is higher in Gopalganj; the weights range between 0.15 and 0.54, indicating a more balanced contribution between the convenience and the probability sample, meaning that some indicators have similar estimates while others do not. In Aurangabad the only indicator having a contribution weight of the HMIS estimator of over 0.35 in all blocks is three ANC visits. Hence, we are not observing a *systematic* discrepancy across indicators and districts. Some HMIS estimates are similar to the probability survey estimates for specific indicators, other indicators display similarity in only certain sub-districts or districts, while other indicators show consistent discrepancies between HMIS and survey result estimates. Ex post, we can learn from these concordance and discordance patterns how well some indicators are defined and measured across districts.

These Bihar-HMIS AT results differ from the Benin and Madagascar-CHD AT results. In the latter study the administrative data consistently over-estimated prevalence [35]. Such is not the case here as 25.6% of indicators over-estimate and 38% under-estimate coverage. Future research should map the variation in data quality by country and indicators as well as sub-nationally, and understand the reasons for the discrepancies between the HMIS and probability sample. These discrepancies might be due to one or more reasons: poor quality denominators in the HMIS in the state or in specific districts; inaccurate or incomplete recording of the HMIS numerator data in one district or at the block-level; or incomplete transmission of HMIS records from a health centre to the block-level for aggregation[38]. It is also possible that the survey and HMIS indicators are not measuring quite the same health system product. The perception of a respondent to a survey question in their own home and that of the health worker in a health centre may differ. Such indicators may need

For peer review only - http://bmjopen.bmj.com/site/about/guidelines.xhtml

replacement as well. Related to this issue is a question of a more general nature. In situations when two estimates differ substantially, is it advisable to combine conflicting estimates? We think this issue can be answered by the data: as the estimates are different, a compromise suggested by the data is to give appropriate weight to the disparate estimates when combining them – a principle which we demonstrate. As to the prescription for future strengthening of the HMIS and DHIS2, this is a separate issue the solution of which depends on information obtained from principled future research.

Our AT method uses statistical principles to combine data coming from different sources and calculates error for all sources as well as for the resulting combined estimate. This innovative product is valuable. The AT approach is different to WHO's well-intentioned attempt to improve HMIS estimates with Computation Logic; its estimates rely on professional judgements and do not produce error terms or CIs–a limitation noted by the authors[39]. Therefore, the risk associated with their resultant is unknown. The Institute for Health Metrics and Evaluation applies more complex quantitative analyses rather than just human judgments. It produces an estimate of the number of additional children covered or overestimated[40]. However, like Computational Logic, it does not report a measure of its error.

We are living in an information conscious era with continual demand for, and the ability to produce, more and better data[1 41] obtained from multiple sources. Using accurate information can improve a population's health and reduce waste. We guide the integration of existing data sources to provide a more complete assessment of national health and to reduce the cost to the health system. While we consider numerous data sources, HMIS, which generally do not produce SEs, are touted as having increasing importance for improving the coverage and quality of health services[42 43]. Yet, HMIS are not accurate. Improving and measuring the quality of health information systems is greatly needed[44]. Our examples exemplify a ubiquitous situation, namely, the difficulty with having a good estimator of the denominator. These problems occur in developed and low resource countries[15], leading some

For peer review only - http://bmjopen.bmj.com/site/about/guidelines.xhtml

policy-makers to call for a restructuring of information systems[44]. The current strategy for improving information involves rolling-out a computerized District Health Information System-2 (DHIS2). Though promising, whether the DHIS2 improves data quality is yet to be determined. Nevertheless, its concern is production of quality numerators. Our study shows that AT detected variation in the quality of the HMIS by indicator and sub-district location, and can be used to strengthen systems like the DHIS2 by identifying indicators and their location where improvement is needed. While it does not tell us the reasons for the errors it shows where they exist and their magnitude. This information can be used to understand and correct the sources of errors, which can improve health programmes and reduce waste.

Although we have discussed in detail the statistical principles of our approach, we should make clear its limitations that the global health community can address. Availability of survey data across multiple districts and sub-districts (Blocks in the case of India) is an issue. Survey data are not always widely available or conducted at a frequency that is needed for timely monitoring of health programmes. Concomitant survey data is essential for using AT to improve routine data. However, one single survey is not necessarily what is needed. Multiple surveys carried out at approximately the same time can be used. What is needed is a central data warehouse where surveys sponsored by government, international and civil society are accessible for use in AT, as are clear descriptions of sampling procedures, and data codebooks. We are early in this era of improving data quality. While we have focused in this paper on developing appropriate statistical solutions, other health systems strengthening innovations are also needed. The international community needs to consider survey data as a public good. Whilst their primary use may be for programme strengthening in a small catchment area, when considered together with other databases, survey data can have an enormous impact on improving data quality in the health system as a whole.

These results indicate the need to establish a service working side by side of the DHIS2 to inculcate hybrid estimation using AT as a standard component of district information systems. It should be an

For peer review only - http://bmjopen.bmj.com/site/about/guidelines.xhtml

independent partner of the DHIS2, under the stewardship of lead international agencies such as UNICEF, the Global Fund to Fight AIDS, Tuberculosis and Malaria, USAID, World Bank and bi-lateral organisations (USAID, DFID). It would work independently and transparently to advance hybrid estimation not only in support of DHIS2, but also, and possibly more importantly, to support local public health practitioners and national policy-makers focus their programs to address priorities.

CONCLUSION

Our statistical innovation, hybrid prevalence estimation using statistical AT, requires concomitant data from a random survey sample[45] conducted at the sub-district-level, and sub-district-level HMIS data. The LQAS survey in Bihar was undertaken for another purpose and so its use in AT was at no additional expense. Other statistical surveys, including the Demographic and Health Survey, are potential candidates. Provided both datasets are available in multiple sub-districts, we can estimate the variability of each indicator between sub-districts, and thus, construct not only a combined estimate but also its 95%CI at both sub-district and district levels. and a 95%CI for the HMIS estimator. With the new visual tool presented here, AT results can be quickly interpreted.

AT is intended to improve the quality and use of the HMIS while reducing waste due to using inaccurate information. This study shows that bringing data from existing household surveys together with HMIS data permits the calculation of more accurate and precise decentralized prevalence measures by combining HMIS and probability samples at very little cost. In addition to measuring coverage, it also allows us to evaluate the HMIS in practice and point to corrective measures. Such results lead to better systems for tracking the public's health.

ACKNOWLEDGMENTS

We gratefully acknowledge the essential roles of Hemant Das, Alok Prahdan and Sanjay Biswa for their careful field work supporting the LQAS survey and the HMIS data retrieval. We thank Prof

For peer review only - http://bmjopen.bmj.com/site/about/guidelines.xhtml

Imelda Bates, Prof Brian Faragher and Nancy Vollmer for their valuable feedback on an earlier version of this manuscript.

AUTHORS' CONTRIBUTIONS

JJV, MP developed the research question; MP, CJ led the development of the mathematical statistics for the annealing technique; JV, BD developed the survey design; JV developed the survey methodology; BD managed the survey and data quality; CJ carried out the statistical analyses; JJV obtained the funding and donor support for the research; JJV, MP, CJ interpreted the data; CJ was responsible for data curation; all authors wrote and reviewed the paper.

FUNDING

This research was funded by the Bill and Melinda Gates Foundation Investment ID OPP1142889

DATA SHARING STATEMENT

Household survey data are available upon reasonable request. The recurrent data belong to the Ministry of Health of the State of Bihar and requests for data must be made to them.

COMPETING INTERESTS

The authors have read the statement on competing interests and declare that they have none.

For peer review only

For peer review only - http://bmjopen.bmj.com/site/about/guidelines.xhtml

REFERENCES

1. Chan M, Kazatchkine M, Lob-Levyt J, et al. Meeting the demand for results and accountability: a call for action on health data from eight global health agencies. *PLoS Med* 2010;7(1):e1000223. doi: 10.1371/journal.pmed.1000223 [published Online First: 2010/02/04]
2. World Health Organization. Sixtieth World Health Assembly: Resolution and Decisions. Geneva: World Health Organisation, 2007.
3. Mavimbe JC, Braa J, Bjune G. Assessing immunization data quality from routine reports in Mozambique. *BMC Public Health* 2005;5:108. doi: 10.1186/1471-2458-5-108
4. Odhiambo-Otieno GW. Evaluation of existing district health management information systems a case study of the district health systems in Kenya. *Int J Med Inform* 2005;74(9):733-44. doi: 10.1016/j.ijmedinf.2005.05.007
5. Aqil A, Lippeveld T, Hozumi D. PRISM framework: a paradigm shift for designing, strengthening and evaluating routine health information systems. *Health Policy Plan* 2009;24(3):217-28. doi: 10.1093/heapol/czp010
6. Mutemwa RI. HMIS and decision-making in Zambia: re-thinking information solutions for district health management in decentralized health systems. *Health Policy Plan* 2006;21(1):40-52. doi: 10.1093/heapol/czj003
7. Cebul RD, Love TE, Jain AK, et al. Electronic health records and quality of diabetes care. *N Engl J Med* 2011;365(9):825-33. doi: 10.1056/NEJMsa1102519
8. Mtema Z, Changalucha J, Cleaveland S, et al. Mobile Phones As Surveillance Tools: Implementing and Evaluating a Large-Scale Intersectoral Surveillance System for Rabies in Tanzania. *PLoS Med* 2016;13(4):e1002002. doi: 10.1371/journal.pmed.1002002
9. Kawonga M, Blaauw D, Fonn S. Aligning vertical interventions to health systems: a case study of the HIV monitoring and evaluation system in South Africa. *Health research policy and systems* 2012;10(1):2.
10. Phaswana-Mafuya N, Peltzer K, Ladzani R, et al. Pre- and post-intervention assessment of a PMTCT-programme-strengthening initiative in a rural area of the Eastern Cape, South Africa. *Afr J AIDS Res* 2011;10(1):83-93. doi: 10.2989/16085906.2011.575551
11. Zulu Z, Kunene S, Mkhonta N, et al. Three parallel information systems for malaria elimination in Swaziland, 2010–2015: are the numbers the same? *Public Health Action* 2018;8(1):S13-S17. doi: 10.5588/pha.17.0058
12. Checchi F, Warsame A, Treacy-Wong V, et al. Public health information in crisis-affected populations: a review of methods and their use for advocacy and action. *Lancet* 2017;390(10109):2297-313. doi: 10.1016/S0140-6736(17)30702-X [published Online First: 2017/06/13]
13. Ronveaux O, Rickert D, Hadler S, et al. The immunization data quality audit: verifying the quality and consistency of immunization monitoring systems. *Bull World Health Organ* 2005;83(7):503-10. [published Online First: 2005/09/24]
14. Admon AJ, Bazile J, Makungwa H, et al. Assessing and improving data quality from community health workers: a successful intervention in Neno, Malawi. *Public Health Action* 2013;3(1):56-59. doi: 10.5588/pha.12.0071 [published Online First: 2013/03/21]
15. Woodworth GF, Baird CJ, Garces-Ambrossi G, et al. Inaccuracy of the administrative database: comparative analysis of two databases for the diagnosis and treatment of intracranial aneurysms. *Neurosurgery* 2009;65(2):251-6; discussion 56-7. doi: 10.1227/01.NEU.0000347003.35690.7A [published Online First: 2009/07/25]
16. Hedt BL, Pagano M. Health indicators: eliminating bias from convenience sampling estimators. *Stat Med* 2011;30(5):560-8. doi: 10.1002/sim.3920
17. Jeffery C, Pagano M, Hemingway J, et al. Hybrid prevalence estimation: Method to improve intervention coverage estimations. *Proc Natl Acad Sci U S A* 2018;115(51):13063-68. doi: 10.1073/pnas.1810287115 [published Online First: 2018/12/07]

For peer review only - http://bmjopen.bmj.com/site/about/guidelines.xhtml

18. Bowley AL. Measurement of the precision attained in sampling. *Bulletin of the International Statistical Institute, 22, Supplement to Liv 1, [*1926;22(Supplement):6-62.
19. Neyman J. On the two different aspects of the representative method: the method of stratified and the method of purposive selection. *Journal of the Royal Statistical Society* 1934;97:558-606.
20. Campbell DT, Stanley J. Quasi Experimental Design. Chicago: Rand McNally 1963.
21. Wilbur CL. Vital statistics for the twelfth United States Census. *Quarterly Publications Journal of the American Statistical Association* 1896-97;5:188-200.
22. Observations et exp6riences concernant des d6nombrements repr6sentatifs. Bulletin of the International Statistical Institute; 1895-6; Berne.
23. Maina I, Wanjala P, Soti D, et al. Using health-facility data to assess subnational coverage of maternal and child health indicators, Kenya. *Bull World Health Organ* 2017;95(10):683-94. doi: 10.2471/BLT.17.194399 [published Online First: 2017/11/18]
24. Simmons EM, Singh K, Mpiima J, et al. Assessing coverage of essential maternal and child health interventions using health-facility data in Uganda. *Popul Health Metr* 2020;18(1):26. doi: 10.1186/s12963-020-00236-x [published Online First: 2020/10/11]
25. Care-Family-Health. Integrated Family Health Initiative: Catalysing change for healthy communities. Washington, D.C.: CARE, 2013.
26. NFHS. National Family Health Survey, India. India: Government of India (GOI), 2017.
27. Valadez JJ, Devkota B, Jeffery C, et al. How well do mothers recall their own and their infants' perinatal events? A two-district study using cross-sectional stratified random sampling in Bihar, India. *BMJ Open* 2019;9(12):e031289. doi: 10.1136/bmjopen-2019-031289 [published Online First: 2019/12/21]
28. Robertson SE, Valadez JJ. Global review of health care surveys using lot quality assurance sampling (LQAS), 1984–2004. *Social Science & Medicine* 2006;63:1648-60.
29. Valadez JJ. Assessing child survival programs in developing countries: testing lot quality assurance sampling. Boston: Harvard University Press 1991.
30. Turner AG, Magnani RJ, Shuaib M. A not quite as quick but much cleaner alternative to the Expanded Programme on Immunization (EPI) Cluster Survey design. *International journal of epidemiology* 1996;25(1):198-203.
31. Davis RH, Valadez JJ. Improving the collection of knowledge, attitude and practice data with community surveys: a comparison of two second-stage sampling methods. *Health Policy Plan* 2014;29(8):1054-60. doi: 10.1093/heapol/czt088
32. Wilbur C. Hadden BD, Hemant Das, Caroline Jeffery, Joseph J. Valadez,. Classical LQAS Compared to CARE LQAS+. Liverpool: Liverpool School of Tropical Medicine, 2017.
33. Valadez JJ, Weiss W, Leburg C, et al. Assessing Community Health Programs: A Trainer's Guide. Using LQAS for Baseline Surveys and Regular Monitoring. St Albans: Teaching-aids At Low Cost 2007.
34. Anoke SC, Mwai P, Jeffery C, et al. Comparing two survey methods of measuring health-related indicators: Lot Quality Assurance Sampling and Demographic Health Surveys. *Trop Med Int Health* 2015;20(12):1756-70. doi: 10.1111/tmi.12605
35. Jeffery C, Pagano M, Hemingway J, et al. Hybrid prevalence estimation: a method to improve intervention coverage estimations. *Proceedings of the National Academy of Sciences* In Press
36. Louis TA. Confidence intervals for a binomial parameter after observing no successes. *The American Statistician* 1981;35(3):154.
37. Rosner B. Fundamentals of Biostatistics. Belmont, CA: Thomson-Brooks/Cole 2006.
38. Chaulagai CN, Moyo CM, Koot J, et al. Design and implementation of a health management information system in Malawi: issues, innovations and results. *Health Policy Plan* 2005;20(6):375-84. doi: 10.1093/heapol/czi044
39. Burton A, Kowalski R, Gacic-Dobo M, et al. A formal representation of the WHO and UNICEF estimates of national immunization coverage: a computational logic approach. *PLoS One* 2012;7(10):e47806. doi: 10.1371/journal.pone.0047806 [published Online First: 2012/11/08]

For peer review only - http://bmjopen.bmj.com/site/about/guidelines.xhtml

40. Lim SS, Stein DB, Charrow A, et al. Tracking progress towards universal childhood immunisation and the impact of global initiatives: a systematic analysis of three-dose diphtheria, tetanus, and pertussis immunisation coverage. *Lancet* 2008;372(9655):2031-46. doi: 10.1016/S0140-6736(08)61869-3 [published Online First: 2008/12/17]
41. Boerma T, Eozenou P, Evans D, et al. Monitoring progress towards universal health coverage at country and global levels. *PLoS Med* 2014;11(9):e1001731. doi: 10.1371/journal.pmed.1001731
42. Bruce C. Health management information systems in lower income countries: an analysis of system design, implementation and utilization in Ghana and Nepal. Amsterdam: Royal Tropical Institute, 1997.
43. Lippeveld Theo, Suerborn R, Bodart C. Design and implementation of health information systems. Geneva: World Health Organization 2000.
44. Lippeveld T, Sauerborn R, Sapirie S. Health information systems--making them work. *World Health Forum* 1997;18(2):176-84.
45. Bethlehem JG. The rise of survey sampling. In: Institute ASoER, ed. CBS Discussion Paper: 1572-0314. The Hague: Statistics Netherlands, 2009:28.

For peer review only

For peer review only - http://bmjopen.bmj.com/site/about/guidelines.xhtml

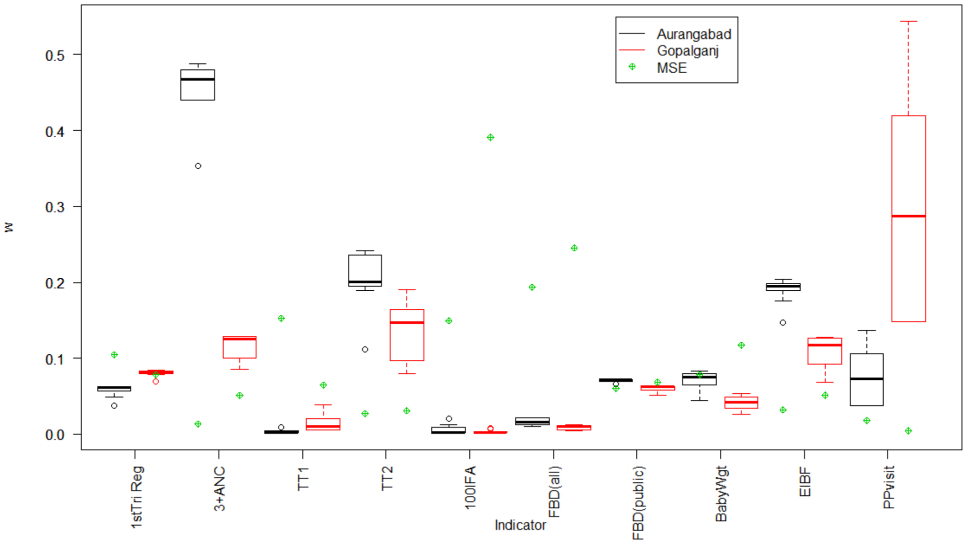

407x257mm (72 x 72 DPI)

Block or district	1stTri Reg	3+ANC	TT1	TT2	100IFA	FBD(all)	FBD(public)	BabyWgt	EIBF	PPvisit
Aurangabad Sadar	green	green	blue	green	lightred	blue	blue	blue	blue	green
Barun	green	lightblue	blue	green	red	blue	blue	blue	blue	green
Daudnagar	green	green	blue	lightred	red	blue	green	green	green	green
Deo	green	green	blue	green	red	blue	green	green	green	green
Goh	red	green	blue	lightred	red	lightblue	green	green	green	green
Haspura	green	green	blue	lightred	green	blue	green	green	green	green
Kutumba	red	green	blue	green	red	blue	lightblue	lightblue	green	green
Madanpur	red	green	blue	lightred	red	blue	lightblue	green	lightred	lightred
Nabinagar	red	green	lightblue	green	red	blue	lightblue	green	green	red
Obra	lightred	green	blue	green	red	blue	blue	blue	green	lightred
Rafiganj	red	green	blue	green	red	lightblue	green	lightred	green	lightred
**Aurangabad District Average**	**red**	**green**	**blue**	**lightred**	**red**	**blue**	**blue**	**lightblue**	**lightblue**	**lightred**
Baikunthpur	lightred	lightred	blue	green	red	blue	lightblue	lightblue	green	green
Barauli	red	red	green	lightred	red	blue	lightblue	green	lightblue	green
Bhorey	green	green	lightblue	green	red	blue	green	green	lightblue	lightred
Bijaipur	red	green	lightblue	green	red	blue	blue	blue	blue	lightred
Gopalganj Sadar	red	lightred	green	lightred	red	blue	blue	blue	blue	green
Hathua	lightred	red	blue	lightred	red	blue	green	blue	green	green
Kateya	red	green	green	red	red	blue	lightblue	lightblue	green	green
Kuchaikote	red	red	lightblue	green	red	blue	blue	blue	green	green
Manjha	green	lightblue	lightblue	green	red	blue	blue	blue	lightblue	green
Pach Deuri	green	green	blue	blue	red	blue	lightblue	green	blue	green
Phulwaria	green	green	blue	lightblue	red	blue	green	lightblue	blue	green
Sidhwalia	red	green	blue	green	red	blue	blue	blue	lightblue	green
Thawe	lightred	lightblue	blue	lightred	red	blue	lightblue	blue	blue	green
Uchkagaon	red	green	lightblue	lightred	red	blue	blue	blue	green	lightred
**Gopalganj District Average**	**red**	**lightred**	**blue**	**lightred**	**red**	**blue**	**blue**	**blue**	**blue**	**lightred**

281x148mm (300 x 300 DPI)

Supplementary Information 1

Statistical Annealing Theory

To construct a standard error (SE) for the HMIS coverage estimator, we use the average mean square error (MSE) between the HMIS and LQAS coverage estimators over all the blocks composing one district. The MSE is calculated for the whole district, and can be decomposed in two ways:

$$MSE = \frac{1}{K}\sum_{k=1}^{K}(p_{HMIS\_k} - p_{LQAS\_k})^2 \quad \text{(first decomposition)}$$
$$= \sigma_{HMIS}^2 + \sigma_{LQAS}^2 \quad \text{(second decomposition)}$$

where:

* K is the total number of blocks in the district;
* k is the index for the block in the concerned district, and ranges from 1 to K;
* σ_{HMIS} is the SE of the HMIS coverage p_{HMIS};
* σ_{LQAS} is the SE of the LQAS coverage p_{LQAS}.

The first decomposition measures how much the HMIS and LQAS coverage estimators differ across the blocks composing the district. The second decomposition shows the MSE is also the sum of the two variances of HMIS and LQAS estimators, assuming each source of data was collected independently from each other. This decomposition also assumes that both HMIS and LQAS are unbiased, i.e. that the expectation of both estimates is the same). We calculate the MSE using the first decomposition and subtract the LQAS variance to obtain the HMIS variance (and thus σ_{HMIS}).

Combined coverage estimator, SE and confidence interval

We define the combined coverage estimator and its SE as a weighted average of the HMIS and LQAS estimators:

$$p_{combined} = w * p_{HMIS} + (1 - w) * p_{LQAS}$$
$$\sigma_{combined} = \sqrt{w^2 * \sigma_{HMIS}^2 + (1 - w)^2 * \sigma_{LQAS}^2}$$

Where the weighting factor w takes values between 0 and 1:

$$w = \frac{\sigma_{LQAS}^2}{\sigma_{HMIS}^2 + \sigma_{LQAS}^2} = \frac{\sigma_{LQAS}^2}{MSE - \sigma_{LQAS}^2 + \sigma_{LQAS}^2} = \frac{\sigma_{LQAS}^2}{MSE}$$

The 95% CI for the combined coverage estimator is calculated using the standard formula, $p_{combined} \pm 1.96 \times \sigma_{combined}$.

For peer review only

Supplementary Information 2

ANC & Birth preparedness indicators

For the proportion of women who had 3 or more ANC during their pregnancy, the weighting factor ranges from 0.35 to 0.49 in Aurangabad, and from 0.09 to 0.13 in Gopalganj (Table S1). The combined estimate differs from the LQAS estimate by no more than 10%. Standard errors for the HMIS data are 2.8 to 3.4 times larger than those calculated for the combined estimates.

Table S1: Women who had 3 or more ANC visits during their pregnancy.

Block	p_{HMIS}	σ_{HMIS}	p_{lqas}	σ_{lqas}	w	$p_{combined}$	$\sigma_{combined}$	95% CI	σ_{HMIS} / $\sigma_{combined}$
Aurangabad Sadar	0.55	0.08	0.55	0.08	0.48	0.55	0.058	(0.44,0.66)	1.44
Barun	0.48	0.09	0.76	0.07	0.35	0.66	0.055	(0.56,0.77)	1.68
Daudnagar	0.71	0.08	0.53	0.08	0.49	0.62	0.058	(0.5,0.73)	1.43
Deo	0.6	0.08	0.58	0.08	0.48	0.59	0.058	(0.48,0.7)	1.45
Goh	0.67	0.09	0.66	0.08	0.44	0.66	0.058	(0.55,0.78)	1.51
Haspura	0.54	0.08	0.61	0.08	0.47	0.58	0.058	(0.46,0.69)	1.46
Kutumba	0.8	0.09	0.66	0.08	0.44	0.72	0.058	(0.61,0.83)	1.51
Madanpur	0.7	0.09	0.66	0.08	0.44	0.67	0.058	(0.56,0.79)	1.51
Nabinagar	0.62	0.08	0.55	0.08	0.48	0.58	0.058	(0.47,0.7)	1.44
Obra	0.73	0.09	0.66	0.08	0.44	0.69	0.058	(0.58,0.8)	1.51
Rafiganj	0.61	0.08	0.61	0.08	0.47	0.61	0.058	(0.49,0.72)	1.46
**Aurangabad District Average**	**0.64**	**0.03**	**0.62**	**0.02**		**0.63**	**0.018**	**(0.6,0.67)**	**1.49**
Baikunthpur	0.72	0.21	0.53	0.08	0.13	0.55	0.076	(0.4,0.7)	2.79
Barauli	0.77	0.21	0.45	0.08	0.13	0.49	0.075	(0.34,0.64)	2.8
Bhorey	0.7	0.21	0.58	0.08	0.13	0.59	0.075	(0.45,0.74)	2.82
Bijaipur	0.8	0.21	0.74	0.07	0.1	0.74	0.068	(0.61,0.88)	3.17
Gopalganj Sadar	0.8	0.21	0.55	0.08	0.13	0.58	0.075	(0.44,0.73)	2.8
Hathua	0.97	0.21	0.47	0.08	0.13	0.54	0.076	(0.39,0.69)	2.79
Kateya	0.8	0.21	0.74	0.07	0.1	0.74	0.068	(0.61,0.88)	3.17
Kuchaikote	0.9	0.21	0.47	0.08	0.13	0.53	0.076	(0.38,0.68)	2.79
Manjha	0.61	0.22	0.79	0.07	0.09	0.77	0.063	(0.65,0.9)	3.42
Pach Deuri	0.5	0.21	0.47	0.08	0.13	0.48	0.076	(0.33,0.62)	2.79
Phulwaria	0.56	0.21	0.58	0.08	0.13	0.58	0.075	(0.43,0.72)	2.82
Sidhwalia	0.72	0.21	0.66	0.08	0.12	0.67	0.072	(0.52,0.81)	2.94
Thawe	0.62	0.22	0.79	0.07	0.09	0.77	0.063	(0.65,0.9)	3.42
Uchkagaon	0.75	0.22	0.76	0.07	0.09	0.76	0.066	(0.63,0.89)	3.28

For peer review only

**Gopalganj District Average**	**0.75**	**0.06**	**0.59**	**0.02**		**0.61**	**0.021**	**(0.57,0.65)**	**2.91**

For the proportion of women who received TT1 during their pregnancy, the weighting factor ranges from 0.002 to 0.009 in Aurangabad, and from 0.006 to 0.038 in Gopalganj (Table S2). The combined estimate differs from the LQAS estimate by no more than 1%. Standard errors for the HMIS data are 5.1 to 20.2 times larger than those calculated for the combined estimates.

Table S2 Women who received TT1 during their pregnancy.

Block	p_{HMIS}	σ_{HMIS}	p_{lqas}	σ_{lqas}	w	$p_{combined}$	$\sigma_{combined}$	95% CI	$\sigma_{HMIS} / \sigma_{combined}$
Aurangabad Sadar	0.64	0.39	0.95	0.04	0.01	0.94	0.036	(0.87,1)	10.78
Barun	0.61	0.39	0.97	0.03	0	0.97	0.026	(0.92,1)	15.04
Daudnagar	0.61	0.39	1	0.02	0	1	0.019	(0.96,1)	20.19
Deo	0.59	0.39	0.97	0.03	0	0.97	0.026	(0.92,1)	15.04
Goh	0.54	0.39	0.95	0.04	0.01	0.94	0.036	(0.87,1)	10.78
Haspura	0.59	0.39	1	0.02	0	1	0.019	(0.96,1)	20.19
Kutumba	0.62	0.39	1	0.02	0	1	0.019	(0.96,1)	20.19
Madanpur	0.52	0.39	1	0.02	0	1	0.019	(0.96,1)	20.19
Nabinagar	0.87	0.39	0.97	0.03	0	0.97	0.026	(0.92,1)	15.04
Obra	0.54	0.39	1	0.02	0	1	0.019	(0.96,1)	20.19
Rafiganj	0.54	0.39	1	0.02	0	1	0.019	(0.96,1)	20.19
**Aurangabad District Average**	**0.61**	**0.12**	**0.98**	**0.01**		**0.98**	**0.008**	**(0.97,1)**	**15.6**
Baikunthpur	0.65	0.25	1	0.02	0.01	1	0.019	(0.96,1)	13.15
Barauli	0.95	0.25	0.95	0.04	0.02	0.95	0.036	(0.88,1)	7.02
Bhorey	0.88	0.25	1	0.02	0.01	1	0.019	(0.96,1)	13.15
Bijaipur	0.81	0.25	0.95	0.04	0.02	0.94	0.036	(0.87,1)	7.02
Gopalganj Sadar	0.97	0.25	0.95	0.04	0.02	0.95	0.036	(0.88,1)	7.02
Hathua	0.48	0.25	0.97	0.03	0.01	0.97	0.026	(0.92,1)	9.79
Kateya	0.84	0.25	0.89	0.05	0.04	0.89	0.049	(0.8,0.99)	5.11
Kuchaikote	0.93	0.25	1	0.02	0.01	1	0.019	(0.96,1)	13.15
Manjha	0.75	0.25	0.92	0.04	0.03	0.92	0.043	(0.83,1)	5.81
Pach Deuri	0.6	0.25	1	0.02	0.01	1	0.019	(0.96,1)	13.15
Phulwaria	0.63	0.25	1	0.02	0.01	1	0.019	(0.96,1)	13.15
Sidhwalia	0.68	0.25	0.97	0.03	0.01	0.97	0.026	(0.92,1)	9.79
Thawe	0.72	0.25	1	0.02	0.01	1	0.019	(0.96,1)	13.15
Uchkagaon	0.87	0.25	0.95	0.04	0.02	0.95	0.036	(0.88,1)	7.02

For peer review only

**Gopalganj District Average**	**0.78**	**0.07**	**0.97**	**0.01**		**0.97**	**0.008**	**(0.95,0.98)**	**8.82**

For the proportion of women who received TT2 during their pregnancy, the weighting factor ranges from 0.11 to 0.24 in Aurangabad, and from 0.08 to 0.19 in Gopalganj (Table S3). The combined estimate differs from the LQAS estimate by no more than 7%. Standard errors for the HMIS data are 2 to 3.5 times larger than those calculated for the combined estimates.

Table S3 Women who received TT2 during their pregnancy.

Block	p_{HMIS}	σ_{HMIS}	p_{lqas}	σ_{lqas}	w	$p_{combined}$	$\sigma_{combined}$	95% CI	$\sigma\_HMIS / \sigma\_combined$
Aurangabad Sadar	0.55	0.14	0.63	0.08	0.23	0.61	0.069	(0.48,0.75)	2.1
Barun	0.8	0.15	0.71	0.07	0.2	0.73	0.066	(0.6,0.86)	2.23
Daudnagar	0.82	0.14	0.61	0.08	0.23	0.66	0.069	(0.52,0.79)	2.07
Deo	0.78	0.15	0.74	0.07	0.19	0.75	0.064	(0.62,0.87)	2.3
Goh	0.8	0.14	0.58	0.08	0.24	0.63	0.07	(0.5,0.77)	2.05
Haspura	0.8	0.14	0.58	0.08	0.24	0.63	0.07	(0.49,0.77)	2.05
Kutumba	0.82	0.15	0.71	0.07	0.2	0.73	0.066	(0.6,0.86)	2.23
Madanpur	0.84	0.14	0.55	0.08	0.24	0.62	0.07	(0.48,0.76)	2.03
Nabinagar	0.79	0.15	0.87	0.05	0.11	0.86	0.052	(0.76,0.96)	2.99
Obra	0.85	0.15	0.74	0.07	0.19	0.76	0.064	(0.63,0.88)	2.3
Rafiganj	0.85	0.15	0.71	0.07	0.2	0.74	0.066	(0.61,0.87)	2.23
**Aurangabad District Average**	**0.8**	**0.05**	**0.68**	**0.02**		**0.71**	**0.02**	**(0.67,0.75)**	**2.26**
Baikunthpur	0.86	0.17	0.87	0.05	0.1	0.87	0.052	(0.77,0.97)	3.22
Barauli	1.04	0.17	0.89	0.05	0.08	0.91	0.048	(0.81,1)	3.54
Bhorey	0.85	0.17	0.87	0.05	0.1	0.87	0.052	(0.76,0.97)	3.22
Bijaipur	0.9	0.17	0.84	0.06	0.11	0.85	0.056	(0.74,0.96)	2.98
Gopalganj Sadar	0.98	0.16	0.71	0.07	0.17	0.76	0.067	(0.63,0.89)	2.4
Hathua	0.93	0.16	0.79	0.07	0.14	0.81	0.061	(0.69,0.93)	2.67
Kateya	1.03	0.16	0.71	0.07	0.17	0.77	0.067	(0.64,0.9)	2.4
Kuchaikote	0.87	0.17	0.89	0.05	0.08	0.89	0.048	(0.8,0.99)	3.54
Manjha	0.87	0.16	0.76	0.07	0.15	0.78	0.063	(0.65,0.9)	2.56
Pach Deuri	0.4	0.16	0.74	0.07	0.16	0.68	0.065	(0.55,0.81)	2.47
Phulwaria	0.7	0.17	0.87	0.05	0.1	0.85	0.052	(0.75,0.95)	3.22
Sidhwalia	0.74	0.16	0.76	0.07	0.15	0.76	0.063	(0.63,0.88)	2.56
Thawe	0.84	0.16	0.66	0.08	0.19	0.69	0.069	(0.56,0.83)	2.29
Uchkagaon	0.92	0.16	0.76	0.07	0.15	0.79	0.063	(0.66,0.91)	2.56

For peer review only

**Gopalganj District Average**	**0.87**	**0.05**	**0.81**	**0.02**		**0.82**	**0.016**	**(0.79,0.85)**	**2.96**

For the proportion of women who received at least 100 IFA tablets during their pregnancy, the weighting factor ranges from 0.002 to 0.02 in Aurangabad, and from 0.001 to 0.01 in Gopalganj (Table S4). The combined estimate differs from the LQAS estimate by no more than 1%. Standard errors for the HMIS data are 7 to 32.3 times larger than those calculated for the combined estimates.

Table S4 Women who received at least 100 IFA tablets during their pregnancy.

Block	p_{HMIS}	σ_{HMIS}	p_{lqas}	σ_{lqas}	w	$p_{combined}$	$\sigma_{combined}$	95% CI	σ_HMIS / $\sigma\_combined$
Aurangabad Sadar	0.13	0.39	0.05	0.04	0.01	0.05	0.036	(0,0.12)	10.68
Barun	0.46	0.39	0.03	0.03	0	0.03	0.026	(0,0.08)	14.9
Daudnagar	0.59	0.39	0	0.02	0	0	0.019	(0,0.04)	20.01
Deo	0.45	0.39	0	0.02	0	0	0.019	(0,0.04)	20.01
Goh	0.18	0.39	0	0.02	0	0	0.019	(0,0.04)	20.01
Haspura	0.16	0.38	0.08	0.04	0.01	0.08	0.043	(0,0.17)	8.85
Kutumba	0.34	0.39	0.05	0.04	0.01	0.06	0.036	(0,0.13)	10.68
Madanpur	0.42	0.39	0	0.02	0	0	0.019	(0,0.04)	20.01
Nabinagar	0.6	0.38	0.13	0.05	0.02	0.14	0.054	(0.03,0.25)	7.06
Obra	0.34	0.39	0	0.02	0	0	0.019	(0,0.04)	20.01
Rafiganj	0.51	0.39	0	0.02	0	0	0.019	(0,0.04)	20.01
**Aurangabad District Average**	**0.39**	**0.12**	**0.03**	**0.01**		**0.04**	**0.01**	**(0.02,0.05)**	**12.02**
Baikunthpur	0.52	0.62	0.03	0.03	0	0.03	0.026	(0,0.08)	24.08
Barauli	0.4	0.62	0	0.02	0	0	0.019	(0,0.04)	32.33
Bhorey	0.79	0.62	0.11	0.05	0.01	0.11	0.05	(0.01,0.21)	12.56
Bijaipur	0.29	0.62	0.03	0.03	0	0.03	0.026	(0,0.08)	24.08
Gopalganj Sadar	0.63	0.62	0.05	0.04	0	0.05	0.036	(0,0.13)	17.26
Hathua	0.86	0.62	0.03	0.03	0	0.03	0.026	(0,0.08)	24.08
Kateya	0.8	0.62	0.03	0.03	0	0.03	0.026	(0,0.08)	24.08
Kuchaikote	0.6	0.62	0.03	0.03	0	0.03	0.026	(0,0.08)	24.08
Manjha	0.36	0.62	0.05	0.04	0	0.05	0.036	(0,0.12)	17.26
Pach Deuri	1.47	0.62	0.13	0.05	0.01	0.14	0.055	(0.03,0.25)	11.4
Phulwaria	0.4	0.62	0.05	0.04	0	0.05	0.036	(0,0.12)	17.26
Sidhwalia	0.38	0.62	0.05	0.04	0	0.05	0.036	(0,0.12)	17.26
Thawe	0.59	0.62	0.05	0.04	0	0.05	0.036	(0,0.13)	17.26
Uchkagaon	0.43	0.62	0	0.02	0	0	0.019	(0,0.04)	32.33

For peer review only

**Gopalganj District Average**	**0.59**	**0.18**	**0.04**	**0.01**		**0.04**	**0.009**	**(0.03,0.06)**	**20.2**

Birth history indicators

For the proportion of infants who had institutional delivery, the weighting factor ranges from 0.01 to 0.02 in Aurangabad, and from 0.005 to 0.01 in Gopalganj (Table S5). The combined estimate differs from the LQAS estimate by no more than 1%. Standard errors for the HMIS data are 6.7 to 14.6 times larger than those calculated for the combined estimates.

Table S5 Institutional Deliveries (all institutions)

Block	p_{HMIS}	σ_{HMIS}	p_{lqas}	σ_{lqas}	w	$p_{combined}$	$\sigma_{combined}$	95% CI	σ_HMIS / σ_combined
Aurangabad Sadar	0.03	0.44	0.88	0.04	0.01	0.87	0.043	(0.78,0.95)	10.11
Barun	0.28	0.44	0.82	0.05	0.01	0.82	0.05	(0.72,0.92)	8.73
Daudnagar	0.41	0.44	0.81	0.05	0.01	0.8	0.052	(0.7,0.9)	8.41
Deo	0.38	0.43	0.63	0.06	0.02	0.63	0.063	(0.5,0.75)	6.88
Goh	0.38	0.43	0.6	0.06	0.02	0.59	0.064	(0.47,0.72)	6.77
Haspura	0.51	0.44	0.77	0.06	0.02	0.77	0.055	(0.66,0.88)	7.91
Kutumba	0.41	0.44	0.86	0.05	0.01	0.85	0.046	(0.77,0.94)	9.56
Madanpur	0.4	0.44	0.7	0.06	0.02	0.7	0.06	(0.58,0.81)	7.26
Nabinagar	0.25	0.43	0.63	0.06	0.02	0.62	0.063	(0.5,0.75)	6.88
Obra	0.3	0.44	0.84	0.05	0.01	0.84	0.048	(0.74,0.93)	9.1
Rafiganj	0.38	0.43	0.58	0.07	0.02	0.57	0.065	(0.45,0.7)	6.72
**Aurangabad District Average**	**0.34**	**0.13**	**0.73**	**0.02**		**0.72**	**0.018**	**(0.69,0.76)**	**7.59**
Baikunthpur	0.44	0.49	0.79	0.05	0.01	0.79	0.054	(0.68,0.89)	9.17
Barauli	0.47	0.49	0.84	0.05	0.01	0.84	0.048	(0.74,0.93)	10.25
Bhorey	0.48	0.49	0.81	0.05	0.01	0.8	0.052	(0.7,0.91)	9.47
Bijaipur	0.35	0.49	0.82	0.05	0.01	0.82	0.05	(0.72,0.92)	9.83
Gopalganj Sadar	0	0.49	0.84	0.05	0.01	0.83	0.048	(0.74,0.93)	10.25
Hathua	0.55	0.49	0.91	0.04	0.01	0.91	0.037	(0.84,0.98)	13.21
Kateya	0.48	0.49	0.93	0.03	0	0.93	0.034	(0.86,0.99)	14.63
Kuchaikote	0.34	0.49	0.77	0.06	0.01	0.77	0.055	(0.66,0.87)	8.91
Manjha	0.2	0.49	0.81	0.05	0.01	0.8	0.052	(0.7,0.9)	9.47
Pach Deuri	0.46	0.49	0.77	0.06	0.01	0.77	0.055	(0.66,0.88)	8.91
Phulwaria	0.42	0.49	0.84	0.05	0.01	0.84	0.048	(0.74,0.93)	10.25
Sidhwalia	0.31	0.49	0.81	0.05	0.01	0.8	0.052	(0.7,0.9)	9.47
Thawe	0.33	0.49	0.93	0.03	0	0.93	0.034	(0.86,0.99)	14.63
Uchkagaon	0.32	0.49	0.91	0.04	0.01	0.91	0.037	(0.84,0.98)	13.21

For peer review only

**Gopalganj District Average**	**0.37**	**0.14**	**0.84**	**0.01**		**0.83**	**0.014**	**(0.8,0.86)**	**9.96**

For the proportion of infants who had institutional delivery in a public facility, the weighting factor ranges from 0.066 to 0.073 in Aurangabad, and from 0.05 to 0.06 in Gopalganj (Table S6). The combined estimate differs from the LQAS estimate by no more than 4%. Standard errors for the HMIS data are 3.7 to 4.4 times larger than those calculated for the combined estimates.

Table S6 Public Institutional Deliveries

Block	p_{HMIS}	σ_{HMIS}	p_{lqas}	σ_{lqas}	w	$p_{combined}$	$\sigma_{combined}$	95% CI	$\sigma\_HMIS / \sigma\_combined$
Aurangabad Sadar	0.03	0.24	0.65	0.06	0.07	0.61	0.061	(0.49,0.73)	3.88
Barun	0.28	0.24	0.56	0.07	0.07	0.54	0.063	(0.42,0.67)	3.73
Daudnagar	0.41	0.24	0.51	0.07	0.07	0.5	0.064	(0.38,0.63)	3.7
Deo	0.38	0.24	0.44	0.07	0.07	0.43	0.063	(0.31,0.56)	3.73
Goh	0.38	0.24	0.44	0.07	0.07	0.43	0.063	(0.31,0.56)	3.73
Haspura	0.51	0.24	0.61	0.06	0.07	0.61	0.062	(0.48,0.73)	3.8
Kutumba	0.41	0.24	0.61	0.06	0.07	0.6	0.062	(0.48,0.72)	3.8
Madanpur	0.4	0.24	0.58	0.07	0.07	0.57	0.063	(0.44,0.69)	3.75
Nabinagar	0.25	0.24	0.4	0.06	0.07	0.39	0.063	(0.27,0.52)	3.77
Obra	0.3	0.24	0.56	0.07	0.07	0.54	0.063	(0.42,0.67)	3.73
Rafiganj	0.38	0.24	0.44	0.07	0.07	0.43	0.063	(0.31,0.56)	3.73
**Aurangabad District Average**	**0.34**	**0.07**	**0.52**	**0.02**		**0.51**	**0.019**	**(0.47,0.54)**	**3.76**
Baikunthpur	0.44	0.25	0.58	0.07	0.06	0.57	0.063	(0.45,0.69)	4
Barauli	0.47	0.25	0.61	0.06	0.06	0.61	0.062	(0.48,0.73)	4.06
Bhorey	0.48	0.25	0.56	0.07	0.06	0.56	0.064	(0.43,0.68)	3.98
Bijaipur	0.35	0.25	0.65	0.06	0.06	0.63	0.061	(0.51,0.75)	4.14
Gopalganj Sadar	0	0.25	0.56	0.07	0.06	0.53	0.064	(0.4,0.65)	3.98
Hathua	0.55	0.25	0.65	0.06	0.06	0.64	0.061	(0.52,0.76)	4.14
Kateya	0.48	0.25	0.7	0.06	0.05	0.69	0.059	(0.57,0.81)	4.32
Kuchaikote	0.34	0.25	0.58	0.07	0.06	0.56	0.063	(0.44,0.69)	4
Manjha	0.2	0.25	0.53	0.07	0.06	0.51	0.064	(0.38,0.63)	3.96
Pach Deuri	0.46	0.25	0.6	0.06	0.06	0.59	0.063	(0.46,0.71)	4.03
Phulwaria	0.42	0.25	0.49	0.07	0.06	0.49	0.064	(0.36,0.61)	3.95
Sidhwalia	0.31	0.25	0.72	0.06	0.05	0.7	0.058	(0.58,0.81)	4.4
Thawe	0.33	0.25	0.47	0.07	0.06	0.46	0.064	(0.34,0.59)	3.96
Uchkagaon	0.32	0.25	0.58	0.07	0.06	0.56	0.063	(0.44,0.69)	4

For peer review only

**Gopalganj District Average**	**0.37**	**0.07**	**0.59**	**0.02**		**0.58**	**0.018**	**(0.54,0.61)**	**4.04**

FLW support and Neonatal Health indicators

For the proportion of infants who were weighed during delivery, the weighting factor ranges from 0.04 to 0.08 in Aurangabad, and from 0.03 to 0.05 in Gopalganj (Table S7). The combined estimate differs from the LQAS estimate by no more than 3%. Standard errors for the HMIS data are 3.5 to 6.2 times larger than those calculated for the combined estimates.

Table S7 Infants who were weighed during delivery.

Block	p_{HMIS}	σ_{HMIS}	p_{lqas}	σ_{lqas}	w	$p_{combined}$	$\sigma_{combined}$	95% CI	σ_HMIS / σ_combined
Aurangabad Sadar	0.1	0.27	0.84	0.06	0.04	0.81	0.058	(0.7,0.92)	4.73
Barun	0.49	0.27	0.76	0.07	0.06	0.75	0.067	(0.62,0.88)	4.06
Daudnagar	0.52	0.27	0.63	0.08	0.08	0.62	0.075	(0.48,0.77)	3.58
Deo	0.46	0.27	0.58	0.08	0.08	0.57	0.077	(0.42,0.72)	3.5
Goh	0.52	0.27	0.55	0.08	0.08	0.55	0.077	(0.4,0.7)	3.47
Haspura	0.61	0.27	0.63	0.08	0.08	0.63	0.075	(0.48,0.78)	3.58
Kutumba	0.53	0.27	0.71	0.07	0.07	0.7	0.071	(0.56,0.84)	3.8
Madanpur	0.57	0.27	0.71	0.07	0.07	0.7	0.071	(0.56,0.84)	3.8
Nabinagar	0.66	0.27	0.66	0.08	0.08	0.66	0.074	(0.51,0.8)	3.64
Obra	0.46	0.27	0.82	0.06	0.05	0.8	0.061	(0.68,0.92)	4.45
Rafiganj	0.6	0.27	0.42	0.08	0.08	0.44	0.077	(0.29,0.59)	3.5
**Aurangabad District Average**	**0.51**	**0.08**	**0.66**	**0.02**		**0.65**	**0.022**	**(0.61,0.69)**	**3.75**
Baikunthpur	0.58	0.34	0.76	0.07	0.04	0.76	0.068	(0.62,0.89)	4.96
Barauli	0.52	0.33	0.61	0.08	0.05	0.6	0.077	(0.45,0.75)	4.31
Bhorey	0.65	0.33	0.63	0.08	0.05	0.63	0.076	(0.48,0.78)	4.37
Bijaipur	0.48	0.33	0.74	0.07	0.04	0.73	0.07	(0.59,0.86)	4.79
Gopalganj Sadar	0.05	0.33	0.74	0.07	0.04	0.71	0.07	(0.57,0.84)	4.79
Hathua	0.54	0.34	0.87	0.05	0.03	0.86	0.054	(0.75,0.97)	6.24
Kateya	0.6	0.34	0.82	0.06	0.03	0.81	0.062	(0.69,0.93)	5.44
Kuchaikote	0.4	0.34	0.79	0.07	0.04	0.77	0.065	(0.65,0.9)	5.17
Manjha	0.29	0.33	0.68	0.08	0.05	0.67	0.074	(0.52,0.81)	4.54
Pach Deuri	0.56	0.33	0.68	0.08	0.05	0.68	0.074	(0.53,0.82)	4.54
Phulwaria	0.5	0.33	0.66	0.08	0.05	0.65	0.075	(0.5,0.8)	4.45
Sidhwalia	0.37	0.34	0.79	0.07	0.04	0.77	0.065	(0.65,0.9)	5.17
Thawe	0.35	0.34	0.82	0.06	0.03	0.8	0.062	(0.68,0.92)	5.44
Uchkagaon	0.44	0.34	0.84	0.06	0.03	0.83	0.058	(0.72,0.94)	5.78

For peer review only

**Gopalganj District Average**	**0.45**	**0.09**	**0.74**	**0.02**		**0.73**	**0.019**	**(0.7,0.77)**	**4.94**

For the proportion of infants who were breast fed within an hour of their birth, the weighting factor ranges from 0.15 to 0.20 in Aurangabad, and from 0.07 to 0.13 in Gopalganj (Table S8). The combined estimate differs from the LQAS estimate by no more than 8%. Standard errors for the HMIS data are 2.2 to 3.8 times larger than those calculated for the combined estimates.

Table S8 Infants who were breast fed within an hour of their birth.

Block	p_{HMIS}	σ_{HMIS}	p_{lqas}	σ_{lqas}	w	$p_{combined}$	$\sigma_{combined}$	95% CI	σ_HMIS / σ_combined
Aurangabad Sadar	0.1	0.16	0.5	0.08	0.2	0.42	0.072	(0.28,0.56)	2.22
Barun	0.49	0.17	0.76	0.07	0.15	0.72	0.064	(0.6,0.85)	2.61
Daudnagar	0.52	0.16	0.42	0.08	0.2	0.44	0.072	(0.3,0.58)	2.24
Deo	0.46	0.16	0.5	0.08	0.2	0.49	0.072	(0.35,0.63)	2.22
Goh	0.52	0.16	0.68	0.08	0.18	0.66	0.068	(0.52,0.79)	2.38
Haspura	0.61	0.16	0.61	0.08	0.19	0.61	0.071	(0.47,0.74)	2.27
Kutumba	0.53	0.16	0.63	0.08	0.19	0.61	0.07	(0.47,0.75)	2.3
Madanpur	0.57	0.16	0.37	0.08	0.19	0.41	0.07	(0.27,0.54)	2.3
Nabinagar	0.66	0.16	0.61	0.08	0.19	0.62	0.071	(0.48,0.76)	2.27
Obra	0.46	0.16	0.63	0.08	0.19	0.6	0.07	(0.46,0.74)	2.3
Rafiganj	0.6	0.16	0.58	0.08	0.2	0.58	0.072	(0.44,0.72)	2.24
**Aurangabad District Average**	**0.51**	**0.05**	**0.58**	**0.02**		**0.57**	**0.022**	**(0.52,0.61)**	**2.3**
Baikunthpur	0.58	0.21	0.66	0.08	0.12	0.65	0.072	(0.51,0.79)	2.94
Barauli	0.52	0.21	0.68	0.08	0.11	0.67	0.071	(0.53,0.81)	3
Bhorey	0.65	0.22	0.84	0.06	0.07	0.83	0.057	(0.72,0.94)	3.83
Bijaipur	0.48	0.22	0.76	0.07	0.09	0.74	0.066	(0.61,0.87)	3.28
Gopalganj Sadar	0.05	0.21	0.53	0.08	0.13	0.47	0.076	(0.32,0.61)	2.79
Hathua	0.58	0.21	0.58	0.08	0.13	0.58	0.075	(0.43,0.73)	2.83
Kateya	0.6	0.21	0.63	0.08	0.12	0.63	0.073	(0.48,0.77)	2.89
Kuchaikote	0.4	0.21	0.55	0.08	0.13	0.53	0.075	(0.39,0.68)	2.81
Manjha	0.3	0.21	0.53	0.08	0.13	0.5	0.076	(0.35,0.65)	2.79
Pach Deuri	0.56	0.22	0.82	0.06	0.08	0.8	0.06	(0.68,0.91)	3.6
Phulwaria	0.5	0.22	0.76	0.07	0.09	0.74	0.066	(0.61,0.87)	3.28
Sidhwalia	0.47	0.21	0.71	0.07	0.11	0.69	0.07	(0.55,0.82)	3.08
Thawe	0.35	0.21	0.63	0.08	0.12	0.6	0.073	(0.45,0.74)	2.89
Uchkagaon	0.44	0.21	0.47	0.08	0.13	0.47	0.076	(0.32,0.62)	2.79

For peer review only

**Gopalganj District Average**	**0.46**	**0.06**	**0.64**	**0.02**		**0.62**	**0.02**	**(0.58,0.66)**	**2.95**

For the proportion of newborns who were visited by any FLW within 24hours of home delivery, the weighting factor ranges from 0.04 to 0.14 in Aurangabad, and from 0.15 to 0.54 in Gopalganj (Table S9). The combined estimate differs from the LQAS estimate by no more than 3%. Standard errors for the HMIS data are 1.4 to 5.2 times larger than those calculated for the combined estimates.

Table S9 Home visit by any FLW within 24 hours of delivery (home deliveries only)

Block	p_{HMIS}	σ_{HMIS}	p_{lqas}	σ_{lqas}	w	$p_{combined}$	$\sigma_{combined}$	95% CI	$\sigma\_HMIS / \sigma\_combined$
Aurangabad Sadar	0.02	0.13	0.03	0.03	0.04	0.03	0.025	(0,0.08)	5.18
Barun	0.16	0.12	0.11	0.05	0.14	0.11	0.046	(0.02,0.2)	2.7
Daudnagar	0	0.13	0.03	0.03	0.04	0.03	0.025	(0,0.08)	5.18
Deo	0.01	0.13	0.08	0.04	0.11	0.07	0.041	(0,0.15)	3.08
Goh	0.03	0.13	0.05	0.04	0.07	0.05	0.035	(0,0.12)	3.71
Haspura	0.1	0.12	0.11	0.05	0.14	0.11	0.046	(0.01,0.2)	2.7
Kutumba	0.08	0.13	0.05	0.04	0.07	0.05	0.035	(0,0.12)	3.71
Madanpur	0.18	0.13	0.05	0.04	0.07	0.06	0.035	(0,0.13)	3.71
Nabinagar	0.4	0.13	0.03	0.03	0.04	0.04	0.025	(0,0.09)	5.18
Obra	0.12	0.13	0.03	0.03	0.04	0.03	0.025	(0,0.08)	5.18
Rafiganj	0.23	0.13	0.08	0.04	0.11	0.1	0.041	(0.01,0.18)	3.08
**Aurangabad District Average**	**0.14**	**0.04**	**0.06**	**0.01**		**0.06**	**0.011**	**(0.04,0.08)**	**3.68**
Baikunthpur	0.09	0.05	0.08	0.04	0.42	0.08	0.033	(0.02,0.15)	1.54
Barauli	0.06	0.05	0.08	0.04	0.42	0.07	0.033	(0,0.14)	1.54
Bhorey	0.18	0.06	0.03	0.03	0.15	0.05	0.024	(0,0.1)	2.6
Bijaipur	0.14	0.06	0.03	0.03	0.15	0.04	0.024	(0,0.09)	2.6
Gopalganj Sadar	0.05	0.06	0.05	0.04	0.29	0.05	0.031	(0,0.11)	1.86
Hathua	0.06	0.05	0.08	0.04	0.42	0.07	0.033	(0,0.13)	1.54
Kateya	0.13	0.06	0.05	0.04	0.29	0.07	0.031	(0.01,0.13)	1.86
Kuchaikote	0.07	0.06	0.03	0.03	0.15	0.03	0.024	(0,0.08)	2.6
Manjha	0.09	0.05	0.11	0.05	0.54	0.1	0.034	(0.03,0.16)	1.36
Pach Deuri	0.11	0.06	0.05	0.04	0.29	0.07	0.031	(0.01,0.13)	1.86
Phulwaria	0.08	0.06	0.05	0.04	0.29	0.06	0.031	(0,0.12)	1.86
Sidhwalia	0.16	0.05	0.08	0.04	0.42	0.11	0.033	(0.05,0.18)	1.54
Thawe	0.03	0.06	0.05	0.04	0.29	0.05	0.031	(0,0.11)	1.86
Uchkagaon	0.12	0.06	0.03	0.03	0.15	0.04	0.024	(0,0.09)	2.6

**Gopalganj District Average**	**0.09**	**0.02**	**0.06**	**0.01**		**0.06**	**0.008**	**(0.05,0.08)**	**1.91**

For peer review only

BMJ Open

An innovative approach to improve information accuracy in a two-district cross-sectional study in Bihar, India

Journal:	*BMJ Open*
Manuscript ID	bmjopen-2021-051427.R2
Article Type:	Original research
Date Submitted by the Author:	06-Dec-2021
Complete List of Authors:	Jeffery, Caroline; Liverpool School of Tropical Medicine, Department of International Public Health<br>Pagano, Marcello; Harvard TH Chan School of Public Health, Department of Biostatistics<br>Devkota, Baburam; Liverpool School of Tropical Medicine, Department of International Public Health<br>Valadez, Joseph; Liverpool School of Tropical Medicine, Department of International Public Health
**Primary Subject Heading**:	Global health
Secondary Subject Heading:	Health informatics, Health policy, Health services research, Public health, Evidence based practice
Keywords:	Health policy < HEALTH SERVICES ADMINISTRATION & MANAGEMENT, Public health < INFECTIOUS DISEASES, Health informatics < BIOTECHNOLOGY & BIOINFORMATICS, International health services < HEALTH SERVICES ADMINISTRATION & MANAGEMENT

SCHOLARONE™
Manuscripts

I, the Submitting Author has the right to grant and does grant on behalf of all authors of the Work (as defined in the below author licence), an exclusive licence and/or a non-exclusive licence for contributions from authors who are: i) UK Crown employees; ii) where BMJ has agreed a CC-BY licence shall apply, and/or iii) in accordance with the terms applicable for US Federal Government officers or employees acting as part of their official duties; on a worldwide, perpetual, irrevocable, royalty-free basis to BMJ Publishing Group Ltd ("BMJ") its licensees and where the relevant Journal is co-owned by BMJ to the co-owners of the Journal, to publish the Work in this journal and any other BMJ products and to exploit all rights, as set out in our licence.

The Submitting Author accepts and understands that any supply made under these terms is made by BMJ to the Submitting Author unless you are acting as an employee on behalf of your employer or a postgraduate student of an affiliated institution which is paying any applicable article publishing charge ("APC") for Open Access articles. Where the Submitting Author wishes to make the Work available on an Open Access basis (and intends to pay the relevant APC), the terms of reuse of such Open Access shall be governed by a Creative Commons licence – details of these licences and which Creative Commons licence will apply to this Work are set out in our licence referred to above.

Other than as permitted in any relevant BMJ Author's Self Archiving Policies, I confirm this Work has not been accepted for publication elsewhere, is not being considered for publication elsewhere and does not duplicate material already published. I confirm all authors consent to publication of this Work and authorise the granting of this licence.

For peer review only

An innovative approach to improve information accuracy in a two-district cross-sectional study in Bihar, India

Authors: Caroline Jeffery[1], Marcello Pagano[2], Baburam Devkota[1], Joseph J Valadez[1]

[1] Department of International Public Health, Liverpool School of Tropical Medicine, Pembroke Place, Liverpool, UK

[2] Department of Biostatistics, Harvard T.H. Chan School of Public Health, 677 Huntington Avenue, Boston, MA, USA

Corresponding author: Joseph J Valadez, joseph.valadez@lstmed.ac.uk

For peer review only

For peer review only - http://bmjopen.bmj.com/site/about/guidelines.xhtml

ABSTRACT

OBJECTIVE

Combine Health Management Information Systems (HMIS) and probability survey data using the statistical annealing technique (AT) to produce more accurate health coverage estimates than either source of data and a measure of HMIS data error.

SETTING

This study is set in Bihar, the 5th poorest state in India., where half the population lives below the poverty line. An important source of data, used by health professionals for programme decision-making is routine health facility or HMIS data. Its quality is sometimes poor or unknown, and has no measure of its uncertainty. Using AT we combine district-level HMIS and probability survey data (n=475) for the first time for 10 indicators assessing antenatal care, institutional delivery and neonatal care from 11 blocks of Aurangabad and 14 blocks of Gopalganj districts (N=6,253,965) in Bihar State, India.

PARTICIPANTS

Both districts are rural. Bihar is 82.7% Hindu and 16.9% Islamic.

PRIMARY OUTCOME MEASURES

Survey prevalence measures for 10 indicators, corresponding prevalences using HMIS data, combined prevalences calculated with AT, and Standard Errors for each type of data.

RESULTS

The combined and survey estimates differ by <0.10. The combined and HMIS estimates differ by up to 84.2%, with the HMIS having 1.4 to 32.3 times larger error. Of 20 HMIS versus survey coverage

For peer review only

estimate comparisons across the two districts only five differed by <0.10. Of 250 sub-district-level comparisons of HMIS versus combined estimates, only 36.4% of the HMIS estimates are within the 95%CI of the combined estimate.

CONCLUSIONS

Our statistical innovation increases the accuracy of information available for local health system decision-making, allows evaluation of indicator accuracy, and increases the accuracy of HMIS estimates. The combined estimates with a measure of error better informs health system professionals about their risks when using HMIS estimates, so they can reduce waste by making better decisions. Our results show that AT is an effective method method ready for additional international assessment while also being used to provide affordable information to improve health services.

STRENGTHS AND LIMITATIONS OF THIS STUDY

- Strength: household survey data were captured as a stratified random sample leading to an efficient use of information.
- Strength: administrative data comprise 100% of the available recurrent information available in the two selected districts.
- Strength: the study population is very large covering a large geographical area, reducing the likelihood that the results are pertinent only to a small group of mothers with children; results may be generalizable.
- Strength: the process for combining probability and administrative data has been assessed using a statistically principled approach prior to use in this study.
- Limitation: the study is confined to two districts of Bihar, India which indicates the need for replicating the study in additional States of India and in other country settings.

review only

KEY WORDS

HMIS, recurrent information systems, hybrid prevalence, combining data, prevalence, indicators

INTRODUCTION

The 17 Sustainable Development Goals (SDGs) were adopted by all United Nations member states in 2015 as an urgent call for action to end poverty and deprivation by following strategies that improve health and education, reduce inequality and spur economic growth. For national strategies to be effective, they must be grounded in the local context. Good quality data to measure the prevalence of disease conditions, or the population's coverage with health services coverage, is an indispensable resource for programme managers and health policy makers to understand their context. This point is equally true in higher income countries as it is in low and middle-income countries (LMIC). In these latter settings, household surveys are generally considered resource intensive, and being carried out at 3-5 year intervals, are not sufficiently frequency for many decisions. As a result, data generated routinely through the Health Management Information Systems (HMIS) is more frequently used for decision-making and for annual reviews than household surveys. HMIS data consists of routine information reported from the health facilities to districts health offices, who submit it to Ministry of Health on a monthly, quarterly, semi-annual and annual basis. A key shortcoming of HMIS data is that it is only representative of those who can access health services. This shortcoming is especially problematic in LMIC where access to healthcare services is constrained by geographic, economic and social determinants, and in settings where there are competing services in the community that do not report into the HMIS.

Demand by global donors for timely and reliable data continues to increase[1]. The Sixtieth World Health Assembly underscored the importance of robust information to strengthen health systems and policies (resolution WHA60.27)[2], and to this end, established a Health Metrics Network (HMN) partnership to aid countries improve data quality of routine information systems to enable their use for health planning and decision-making[3-6]. This was followed by other resolutions and in 2015 by a

For peer review only - http://bmjopen.bmj.com/site/about/guidelines.xhtml

high-level summit on Measurement and Accountability for Results leading to the formation of the Health Data Collaborative (HDC) in March 2016 and supported by global partners. HDC's mandate is more extensive than that of HMN, having an ultimate objective of aiding countries to improve the quality and availability of health data and their ability to consistently and accurately report on progress towards the health-related Sustainable Development Goals.

The advent of electronic health records was heralded, as studies showed their association with improved clinical care, outcomes[7], and surveillance[8]. Consequently, the advancement of a computerised District Health Information System-2 (DHIS2) was implemented in 73+ countries to integrate data sources for their more rapid analyses, dissemination and use. An evaluation in Tanzania showed it improved timeliness and completeness of reporting[8]. South Africa established a dedicated HIV/AIDS information system recommending the use of the earlier DHIS for integrating different information sources[9] and as input for designing national program strategies[10]. A 2010-2015 study in Swaziland recommended embracing electronic medical data systems to reduce the discrepancies occurring between the existing three information systems put in place for malaria elimination[11]. Recently, a similar demand was issued by practitioners working in humanitarian settings for an *epidemiology and demography service* to develop robust and timely information from multiple sources to establish priorities that address the population's needs[12].

One important use of routine information systems is to monitor a population's coverage with health service, as Global Alliance on Vaccines and Immunisations (GAVI) has done for vaccination rates since the 1990s. They attempt to control for known weaknesses in administrative data by carrying out data quality audits by recounting, and contrasting, the number vaccinated in health centres with the number originally reported[13].

Although measurement improves all facets of health management, HMIS related studies mainly focus on improvements in obtaining numerators[1 5 14 15]. The future also demands accurate and precise denominators, and, more importantly, a principled method for assessing their accuracy (e.g.,

For peer review only - http://bmjopen.bmj.com/site/about/guidelines.xhtml

standard errors (SE)) so users understand their risk when using the data. Under normal circumstances, the error present in HMIS remains unknown.

The research question we investigate in this paper: How can we measure the error in the HMIS and improve its accuracy? We address this issue by presenting an innovation called *Statistical Annealing* (Annealing Technique or AT). It builds on our earlier pioneering work in 2011[16] and refined in 2018 [17] that provides a coverage estimator with a 95% Confidence Interval (CI) by combining data from a probability survey and HMIS data. HMIS coverage estimates have not before had a 95% Confidence Interval (CI) to inform users of their accuracy. We demonstrated the magnitude of HMIS error and how it can be improved by using AT.

By the early 20th century Bowley[18] and Neyman[19] proved the importance of sampling and the estimated magnitudes of their errors. The standard error (SE)[20] is a commonly used measure of error, which is an estimate of deviation of the sample mean from the actual population mean. The SE is used in calculating a 95%CI, which relates to the accuracy of the estimate. If survey data are combined (annealed) with HMIS appropriately they produce a single, more accurate coverage estimator[16]. However, concurrent well-designed surveys have been a surprisingly neglected sources of information for improving HMIS [21 22]. Recent efforts have used surveys to improve HMIS estimates[23 24], although the resulting revised estimates do not have a corresponding 95%CI.

The objective of AT is to produce a principled measure of health system performance by combining existing HMIS and survey information which also produces an accompanying measure of its accuracy. This hybrid estimate is more accurate than either data source alone. Our proof of principle study, used Child Health Day (CHD) administrative data from Benin and Madagascar provided by UNICEF country offices and household survey data collected at the same time, to verify CHD coverage and the quality of CHD administrative data[17]. This refined approach to AT resulted in the production of a 95% CI for the administrative data and for the combined result. See Supplementary Information 1 for the AT formulae.

For peer review only - http://bmjopen.bmj.com/site/about/guidelines.xhtml

The current study assesses the transferability of AT to a new setting, HMIS data from Bihar state located in northeast India. Bihar is the 5th poorest state of India where half the population lives below the poverty line. It is one of the most densely populated states (N = 110million), and has some of the weakest maternal and child health and nutrition indicators in India [25 26]. Both districts are primarily rural with a mix of upper caste and lower caste Hindu residents, and minority Muslim communities.

Also, this study is the first use of AT with true HMIS data, which in the future will be a more typical use for AT, and result in our conclusions about its global applicability. We apply AT to 10 indicators related to antenatal care (ANC), institutional delivery, and neonatal care using data from two districts, Aurangabad (N=3,695,928) and Gopalganj (N=2,558,037), and two sources of data collected for purposes other than this study. HMIS data from the two districts and a probability survey data collected as part of an earlier assessment of maternal and child health care coverage in Bihar [27]. This secondary data analysis uses datasets without personal identifiers.

METHODS

HMIS system in Bihar

The HMIS data in Bihar consists of 318 data elements. These are reported monthly by health sub-centres (HSC) and aggregated at the block primary health centre (BPHC). The *block* is the sub-district administrative unit in India. Staff at the HSCs tally information from paper registers and send a paper report to BPHCs where it is entered into a computerized HMIS system, and subsequently maintained electronically. District-level indicators comprise information aggregated across the block as well as data from district and referral hospitals.

For peer review only - http://bmjopen.bmj.com/site/about/guidelines.xhtml

Data

To develop AT in Bihar, we used the health facility-based HMIS and probability data from a household survey in two districts, Aurangabad and Gopalganj. From the Bihar State Health Society, we obtained two sources of HMIS data: the HMIS data reported by all health facilities in the two districts, at district level and disaggregated to the block-level; and the Expected Level of Achievement (ELA), which is projected data. They calculated the ELA data using the latest 2011 census and applied an average annual population growth rate using parameter values reported in the Annual Health Survey 2012-13 (e.g., crude birth rate) and in the HMIS data from previous years (e.g., percentage of pregnancies leading to a still birth in 2015-16). We averaged these two data sources, the HMIS and the ELA, to obtain the administrative estimate.

A household probability survey was conducted during 7-25 September 2016 in each district in a standardised manner[28 29] using Lot Quality Assurance Survey (LQAS). The LQAS survey is a stratified random sample used to assess health service coverage. The strata are administrative blocks (11 in Aurangabad, 14 in Gopalganj), which are sub-district units in India. The strata were weighted by their population size. The first stage sample uses probability proportional to size to randomly sample villages (typically, n=19 per stratum[28]). In each random location a second stage sample identifies an index household using segmentation sampling[30 31] from locally constructed hand-drawn maps. The next closest household is then selected for interview to reduce the likelihood that households not included in the map do not have zero probability of selection. In the selected household, individuals in three target groups (women with children 0-2, 3-5 and 0-5 months of age[32]) are listed with one selected randomly using a random number table. The remaining target group is selected in the next closest house using the same protocol[33]. The resulting sample of individuals is random and provides its own standard error (SE) estimator used for calculating a 95% CI. One member of each target group only was selected in a sampled village.

For peer review only - http://bmjopen.bmj.com/site/about/guidelines.xhtml

The total number of random samples for each indicator is 209 (Aurangabad) and 266 (Gopalganj). LQAS gives reliable estimates for indicators at the district level[34] and allows the classification of blocks, as "performing adequately" or not. However, in this paper we do not use the data for classification, but rather for estimation.

Indicators for annealing

We based the selection of 10 indicators for annealing on the ease of combining HMIS data temporally to the survey data (Table 1). For the LQAS survey, we measured antenatal care (ANC), institutional delivery, and neonatal care outcomes in the population of women with children 0-2, 3-5 and 0-5 months of age[32] resulting in a total of 627 (Aurangabad) and 798 (Gopalganj) interviews. We used the HMIS data from the last 5-months of reports (April-August 2016) preceding the LQAS survey (Table 1, Table 2), so that they matched temporally.

The Annealing Technique design

As in earlier work [35], we apply AT in each sub-district for each indicator. The district-level estimators are an aggregation of appropriate block-level coverage data, weighted by the population sizes. Data analysis was done using Stata SE ver15 and R ver-3.4.1.

Survey coverage estimator

For all but two indicators, the numerator of the LQAS coverage is the sum of correct responses in each of two target group (mothers of children 0-2 months and 0-5 months) in each block. The denominator is the sum of the denominators of both groups $(19 \times 2 = 38)$. For the two indicators we used additional data from a third target group, mothers of children 3-5 months, where the denominator is the sum of the denominators of each target group $(19 \times 3 = 57)$ (Table 2).

A step in the annealing process requires a weighted average of component parts; the LQAS estimator is one such component. For each component, the weight used is inversely proportional to its

For peer review only - http://bmjopen.bmj.com/site/about/guidelines.xhtml

variance. If the LQAS coverage, p, is away from the extremes of zero or one, we can use the standard binomial formula, $p(1-p)/n$. When the LQAS coverage is zero (or one), we calculate the weight by using a surrogate `standard error' from a 95% CI using Louis' approach[36] and dividing its length ($1-0.05^{(1/n)}$) by (2×1.96), and squaring the resultant.

To assess any block-level clustering effect between target groups sampled in parallel, we use the two-tail McNemar's test for correlated proportions for each indicator[37], across all blocks sampled in each district.

HMIS coverage estimator

The numerator of the HMIS coverage is reported in Table 1 (Column 4). The denominator is the average between the total number of ANC registration per month calculated by the ELA projected data and those reported in the HMIS data (averaged over April-August 2016) (Table 2).

For peer review only - http://bmjopen.bmj.com/site/about/guidelines.xhtml

1 Table 1: List of 10 indicators selected for applying the annealing technique in Bihar State, India

Generic name of Indicator	LQAS Indicator	Targeted groups (LQAS surveys)	HMIS indicator	Targeted months (HMIS reported data)
First trimester registration	Proportion of mothers of 0-2 months old whose pregnancy was registered in the first trimester in the last pregnancy	0-2 months<br>0-5 months	Number of pregnant women registered within first trimester	April-August 2016
3 or more ANC	Proportion of mothers of 0-2 months old who had 3 or more ANC's during their entire period of last pregnancy	0-2 months<br>0-5 months	Number of pregnant women received 3 ANC check-ups during pregnancy	April-August 2016
Received TT1	Proportion of mothers of 0-2 months who received TT1 during their last pregnancy	0-2 months<br>0-5 months	Number of pregnant women given TT1 during current pregnancy	April-August 2016
Received TT2	Proportion of mothers of 0-2 months who received TT2 during their last pregnancy	0-2 months<br>0-5 months	Number of pregnant women given TT2 or booster during current pregnancy	April-August 2016
Received 100 IFA tablets	Proportion of mothers of 0-2 months who received at least 100 IFA tablets during their last pregnancy	0-2 months<br>0-5 months	Total number of pregnant women given 100 IFA tablets	April-August 2016
Institutional deliveries (all)	Proportion of 0-3 months infants who had institutional delivery (survey conducted September 2016)	0-2 months<br>3-5 months<br>0-5 months	Total number of deliveries conducted in institutional setting (private and public) including C sections	April-August 2016

For peer review only - http://bmjopen.bmj.com/site/about/guidelines.xhtml

Public Institutional Deliveries	Proportion of 0-3 months infants who had institutional delivery at public facility (survey conducted September 2016)	0-2 months<br>3-5 months<br>0-5 months	Number of deliveries conducted in public institutions including C sections	April-August 2016
Baby weighed during delivery	Proportion of 0-2 months infants who were weighed during delivery	0-2 months<br>0-5 months	Number of new-borns weighed at birth	April-August 2016
Early Initiation of Breast feeding (EIBF)	Proportion of 0-2 months infants who were breast fed within an hour of their birth	0-2 months<br>0-5 months	Number of new-borns breast fed within one hour of birth	April-August 2016
Post-Partum home visit within 24 hours (home-deliveries only)	Home visit by any FLW within 24 hours of delivery	0-2 months<br>0-5 months	Number of new-borns visited within 24 hours of delivery for deliveries conducted at home.	April-August 2016

For peer review only

Table 2: Data characteristics

Characteristics	HMIS	Household Survey
Source	Bihar State Health Society	LSTM study
Sample size	Aurangabad:<br>5917 (ELA)<br>5350 (HMIS)<br>Gopalganj:<br>5952 (ELA)<br>6373 (HMIS)	Aurangabad (11 blocks):<br>19 x 11= 209 per target group<br>Gopalganj (14 blocks):<br>19 x 14 = 266 per target group
Time period	April-August 2016	7-25 September 2016

For peer review only - http://bmjopen.bmj.com/site/about/guidelines.xhtml

To calculate the weight, as we did for the LQAS estimator, to use for the HMIS estimator, we use the fact that both estimators are estimating the same quantity, so their difference should be estimating zero. To construct a variance for the HMIS coverage estimator, we use the average mean square error (MSE), which is the average of the squared difference between the HMIS and LQAS coverage estimates over all the blocks composing one district. The MSE is calculated for the whole district, and can be decomposed in two ways:

$$MSE = \frac{1}{K}\sum_{k=1}^{K}(p_{HMIS\_k} - p_{LQAS\_k})^2 \qquad \text{(first decomposition)}$$

$$= \sigma^2_{HMIS} + \sigma^2_{LQAS} \qquad \text{(second decomposition)}$$

where:

- K is the total number of blocks in the district;
- k is the index for the block in the concerned district, and ranges from 1 to K;
- σ_{HMIS} is the SE of the HMIS coverage p_{HMIS};
- σ_{LQAS} is the SE of the LQAS coverage p_{LQAS}.

The first decomposition measures how much the HMIS and LQAS coverage estimators differ across the blocks composing the district. The second decomposition shows the MSE is also the sum of the two variances of HMIS and LQAS estimators, assuming each source of data was collected independently from each other. This decomposition also assumes that both HMIS and LQAS are unbiased, i.e., that the expectation of both estimates is the same). We calculate the MSE using the first decomposition and subtract the LQAS variance to obtain the HMIS variance (and thus σ_{HMIS}). This can then be used to calculate the weight, as we show in [35] and in the Supplementary Information 1 and 2 (Tables S1-S9).

Assessing variation in local-level data quality

To advance the use of AT in multiple settings we developed a tool for sub-district-level analysis. We assess the variation in the quality of the HMIS indicators in 250 block-level comparisons of the 10 indicators ($10 \times (11\ Aurangabad + 14\ Gopalganj)$) and 20 district-level comparisons. We classify

For peer review only - http://bmjopen.bmj.com/site/about/guidelines.xhtml

the value of the HMIS coverage relative to the CI around the combined estimate. If the HMIS coverage is within 95% CI of $p_{combined}$ the cell is coloured green. If the HMIS estimate is within a reasonable percentage difference of each boundary of the CI, for example 10% (we colour those cells light red if it is above the high boundary or light blue if it is below the lower boundary), we are assured its value is not far from the survey estimate. While when the HMIS coverage is beyond the reasonable percentage difference of each boundary, the discrepancy might be too large to recommend combining the two estimates and to not use the HMIS estimate (we colour these dark red or dark blue).

Ethics Statement

The survey data were collected originally for assessment of a maternal and child health care programme in Bihar. The survey protocol was approved in 2016 by the Indian Institute of Public Health Bhubaneswar Institutional Ethics Committee (IIPHB-IEC-2016/010) and the Ethical Review Board of the Liverpool School of Tropical Medicine (research protocol 16-023RS). We obtained oral rather than written informed consent from all respondents, because of the high illiteracy rate. The survey data we later treated as secondary data for the current study which was supported by our donor and the Bureau of Statistics for the Ministry of Health for the State of Bihar. After giving permission to access the State HMIS, they facilitated the collection of the HMIS data.

Patient and Public Involvement

This study does not involve patients. Also, the public were not involved in the design, conduct and reporting of the research. The public was engaged as interviewees. To ensure local engagement all data capture was carried out in close coordination with the State Ministry of Health of Bihar. We also shared the results with them and offered further dissemination of results, and engaged them for data use and action planning activities.

For peer review only - http://bmjopen.bmj.com/site/about/guidelines.xhtml

RESULTS

Data characteristics and AT characteristics

Among the 10 indicators, the Mean Squared Error (MSE) between the two sources of data ranges from 0.013 to 0.193 in Aurangabad, and between 0.005 and 0.391 in Gopalganj (See Table 3 for the indicators). Three blocks in Gopalganj have an HMIS coverage larger than 100% (two or more tetanus toxoid vaccinations (TT2): 104% in Barauli and 103% in Kateya; iron folic acid (IFA) tablets distribution: 147% in Pach Deuri). The HMIS coverage averaged over blocks ranges between 12% and 80% in Aurangabad, and between 10% and 84% in Gopalganj. The LQAS coverage averaged over the blocks ranges between 3% and 98% in Aurangabad, and between 5% and 97% in Gopalganj. The McNemar tests did not detect block-level clustering except for the four tests with indicators measuring institutional delivery in all facilities or in public facilities.

For peer review only

For peer review only - http://bmjopen.bmj.com/site/about/guidelines.xhtml

Table 3: Summary information before application of AT

District	Indicator	MSE	Average value of p_{HMIS} over all blocks†	Average value of p_{LQAS} over all blocks†	McNemar test p-values ‡
Aurangabad	Pregnancy registered during first 3 months	0.105	0.66	0.42	0.47
	3 or more ANC	0.013	0.64	0.62	0.46
	Mothers who were protected against tetanus during pregnancy (Neonatal TT)	0.152	0.61	0.98	0.71
	Mothers who were protected against tetanus TT2	0.027	0.79	0.67	0.68
	Mothers who received iron folic acid for 100 days or more when they were pregnant	0.150	0.38	0.03	0.71
	Institutional delivery (Both private and public facility)	0.193	0.34	0.74	0-2 vs 3-5mo: 0.73<br>0-2 vs 0-5mo: 0.39<br>0-5 vs 3-5mo: 0.20
	Institutional delivery (public facility)	0.060	0.34	0.53	0-2 vs 3-5mo: 0.05<br>0-2 vs 0-5mo: 0.84<br>0-5 vs 3-5mo: 0.04§
	Baby Weighed within one hour of birth	0.078	0.50	0.67	0.65
	Early Initiation of Breast feeding (EIBF)	0.032	0.50	0.57	0.49
	Home visit by any FLW within 24 hours of delivery	0.018	0.12	0.06	0.18
Gopalganj	Pregnancy registered during first 3 months	0.078	0.72	0.48	0.86
	3 or more ANC	0.051	0.73	0.63	0.37

For peer review only

For peer review only - http://bmjopen.bmj.com/site/about/guidelines.xhtml

	Mothers who were protected against tetanus during pregnancy (Neonatal TT)	0.065	0.76	0.97	0.81
	Mothers who were protected against tetanus TT2 *	0.031	0.84	0.78	0.91
	Mothers who received iron folic acid for 100 days or more when they were pregnant *	0.391	0.63	0.05	0.20
	Institutional delivery (Both private and public facility)	0.245	0.35	0.85	0-2 vs 3-5mo: 0.04[§] 0-2 vs 0-5mo: 0.06 0-5 vs 3-5mo: 0.89
	Institutional delivery (public facility)	0.068	0.35	0.59	0-2 vs 3-5mo: 0.01[§] 0-2 vs 0-5mo: 0.03[§] 0-5 vs 3-5mo: 0.51
	Baby Weighed within one hour of birth	0.117	0.44	0.75	0.05
	Early Initiation of Breast feeding (EIBF)	0.051	0.45	0.65	0.17
	Home visit by any FLW within 24 hours of delivery	0.005	0.10	0.05	0.45

* Three blocks in Gopalganj have an HMIS coverage larger than 100% (TT2 indicator: 104% in Barauli and 103% in Kateya; IFA tablets indicator: 147% in Pach Deuri)

† 11 blocks in Aurangabad and 14 blocks in Gopalganj.

‡ McNemar test comparing 0-2 vs 0-5 months unless otherwise specified.

§ The McNemar test reports a major difference at p <0.05.

For peer review only

The weighting factor used in the calculation of the combined estimator varies for each indicator in each district (Figure 1). For six indicators (registration during first trimester of pregnancy, receiving TT1, receiving ≥100 IFA tablets, institutional delivery in any facility, deliveries in public facilities, baby weighed within 1 hour of birth), all block-level weighting factors w, are below 0.10 in both districts, indicating the contribution of HMIS estimator to the combined estimator is no higher than 10% (Table 3 and Supplementary Information 2 Tables S1-S9). We use a 1-tail test to check for difference of the weights between the two districts, based on the observed mean difference. The weights for Gopalganj are higher than Aurangabad's for three indicators: first trimester registration, receiving TT1 and having a post-partum visit – for all three, one-sided t-test p-values are <0.01. The Gopalganj weighting factors are lower than Aurangabad's for the other seven indicators: ≥3 ANC visits, receiving TT2, receiving ≥100 IFA tablets, institutional delivery in any facility, deliveries in public facilities, early initiation of breastfeeding (EIBF), baby weighed at delivery (all seven one-sided t-test p-values <0.05). This result indicates that there are more discrepancies between the HMIS estimates and the LQAS estimates in Aurangabad when compared to Gopalganj for the first three indicators, while the discrepancies are greater in Gopalganj for the latter seven indicators. All but one of the weights for the indicator ≥3 ANC visits in Aurangabad are between 0.4 and 0.5, indicating an almost equal contribution of the HMIS and LQAS estimators to the resulting combined estimator. Alternatively, there are large variations in the weights for the indicator measuring the one visit by any Frontline Worker (FLW) within 24-hours of delivery in Gopalganj; while, the MSE is small in this instance.

(Insert Figure 1 here)

[Footnote and Title for Figure 1: *Circles with no filling indicate outlier values

Figure 1: Distribution and average of w for each indicator and district*]

For peer review only

For peer review only - http://bmjopen.bmj.com/site/about/guidelines.xhtml

Table 4: Women who registered for ANC during the first three months of pregnancy

Block	p_{HMIS}	σ_{HMIS}	p_{lqas}	σ_{lqas}	w	$p_{combined}$	$\sigma_{combined}$	95% CI	$\sigma_{HMIS}/\ \sigma_{com}$
Aurangabad Sadar	0.42	0.31	0.39	0.08	0.06	0.4	0.077	(0.25,0.55)	4.08
Barun	0.55	0.31	0.58	0.08	0.06	0.58	0.078	(0.43,0.73)	4.04
Daudnagar	0.67	0.31	0.53	0.08	0.06	0.54	0.078	(0.38,0.69)	3.99
Deo	0.65	0.31	0.55	0.08	0.06	0.56	0.078	(0.41,0.71)	4.01
Goh	0.75	0.32	0.18	0.06	0.04	0.21	0.062	(0.08,0.33)	5.14
Haspura	0.46	0.31	0.55	0.08	0.06	0.55	0.078	(0.39,0.70)	4.01
Kutumba	0.78	0.31	0.42	0.08	0.06	0.44	0.078	(0.29,0.60)	4.04
Madanpur	0.74	0.31	0.32	0.08	0.05	0.34	0.073	(0.20,0.48)	4.29
Nabinagar	0.81	0.31	0.37	0.08	0.06	0.39	0.076	(0.25,0.54)	4.13
Obra	0.68	0.31	0.5	0.08	0.06	0.51	0.079	(0.36,0.67)	3.99
Rafiganj	0.77	0.32	0.26	0.07	0.05	0.29	0.070	(0.15,0.42)	4.53
**Aurangabad District Average**	**0.68**	**0.1**	**0.41**	**0.02**		**0.42**	**0.023**	**(0.38,0.47)**	**4.23**
Baikunthpur	0.73	0.27	0.53	0.08	0.08	0.54	0.078	(0.39,0.70)	3.46
Barauli	0.74	0.27	0.39	0.08	0.08	0.42	0.076	(0.27,0.57)	3.53
Bhorey	0.66	0.27	0.53	0.08	0.08	0.54	0.078	(0.39,0.69)	3.46
Bijaipur	0.81	0.27	0.47	0.08	0.08	0.5	0.078	(0.35,0.65)	3.46
Gopalganj Sadar	0.67	0.27	0.37	0.08	0.08	0.39	0.075	(0.24,0.54)	3.58
Hathua	0.94	0.27	0.71	0.07	0.07	0.73	0.071	(0.59,0.87)	3.81
Kateya	0.89	0.27	0.39	0.08	0.08	0.43	0.076	(0.29,0.58)	3.53
Kuchaikote	0.74	0.27	0.39	0.08	0.08	0.42	0.076	(0.27,0.57)	3.53
Manjha	0.67	0.27	0.61	0.08	0.08	0.61	0.076	(0.46,0.76)	3.53
Pach Deuri	0.49	0.27	0.47	0.08	0.08	0.47	0.078	(0.32,0.63)	3.46
Phulwaria	0.61	0.27	0.5	0.08	0.08	0.51	0.078	(0.36,0.66)	3.45
Sidhwalia	0.79	0.27	0.42	0.08	0.08	0.45	0.077	(0.30,0.60)	3.5
Thawe	0.67	0.27	0.42	0.08	0.08	0.44	0.077	(0.29,0.59)	3.5
Uchkagaon	0.71	0.27	0.42	0.08	0.08	0.44	0.077	(0.29,0.59)	3.5
**Gopalganj District Average**	**0.73**	**0.08**	**0.48**	**0.02**		**0.5**	**0.022**	**(0.46,0.54)**	**3.53**

Comparing indicator estimates of 20 p_{HMIS} versus p_{LQAS} across the two districts, five differed by <u><</u>0.10 and six others by <u><</u>0.20. The remaining nine indicator estimates show larger differences (Table 3; Tables S1-S9).

For peer review only - http://bmjopen.bmj.com/site/about/guidelines.xhtml

For peer review only

ANC services

All estimates, SEs and CIs are calculated at the block and district levels (as an illustration, see the detailed results for first trimester ANC registration in Table 4; Supplementary Information 2 Tables S1-S4 for other indicators). For the five indicators measuring coverage related to ANC and birth preparedness, the combined block-level estimate differs from the LQAS estimate by 1% (received ≥100 IFA tablets) to 10% (≥3 ANC visits); the block-level combined estimate differs from the HMIS estimate at most by 28% (TT1) to 133% (received ≥100 IFA tablets). SEs for the HMIS data are 1.4 to 32.3 times larger than those calculated for the combined estimates.

Institutional delivery

For the two indicators measuring the proportion of infants institutionally delivered (Tables S5-S6), the combined block-level estimate differs from the LQAS estimate at most by 1% (all facilities) and 4% (public facilities only); the combined estimate differs from the HMIS estimate at most by 84% (all facilities) and 58% (public facilities only). SEs for the HMIS data are 3.7 to 14.6 times larger than those calculated for the combined estimates.

Neonatal health

For the three indicators measuring coverages related to neonatal health (Supplementary Information 2 Tables S7-S9), the combined block-level estimate differs from the LQAS estimate at most by 3% (newborns who were visited by any FLW within 24-hours of home delivery) to 8% (EIBF); the combined estimate differs from the HMIS estimate at most by 36% (newborns who were visited by any FLW within 24-hours of home delivery) to 70% (EIBF). SEs for the HMIS data are 1.4 to 6.2 times larger than those calculated for the combined estimates.

For peer review only

For peer review only - http://bmjopen.bmj.com/site/about/guidelines.xhtml

Local level variation

We first look at the two rows of Figure 2 that summarize the district level measures and see that, on average, Aurangabad's HMIS measures (row 13) behave slightly better than Gopalganj's (row 28). We see that for two indicators both districts are red; for three they are both blue; for two they are both light red; and for two Aurangabad's are light blue versus Gopalganj's dark blue; and, for one Aurangabad's is green and Gopalganj's is light red.

(Insert Figure 2 here)

[Footnote and Title for Figure 2:

*5 categories: (Blue) HMIS coverage is more than 10% below the lower bound. (Light blue) HMIS coverage is less than 10% below the CI lower bound. (Green) HMIS coverage is within the CI. (Light red) HMIS coverage is less than 10% above the CI upper bound, (Red) HMIS coverage is more than 10% above the CI upper bound.

Figure 2: HMIS coverage value compared to the 95% CI of the $p_{combined}$ estimate for 10 Indicators*]

We get a more detailed contrast when investigating the block-level results. Of the 250 comparisons 36.4% of the HMIS estimates are within the CI of the combined estimate with Aurangabad displaying greater accuracy (44.5%) than Gopalganj (30%). For two indicators (100 IFA tablets received and facility based delivery (all)) the results are consistently poor across blocks. The same can be said of TT1 in Aurangabad, but not in Gopalganj. These indicators warrant further investigation into why the two sources of data have such discrepancies. 3+ANC displays good agreement in Aurangabad, but a mixed evaluation in Gopalganj. Similarly, with the remaining indicators, we can detect subtly different results within the two districts. These results suggest substantial variation across blocks, districts and indicators in the HMIS accuracy.

For peer review only

DISCUSSION

The results of our study show numerous discrepancies of block-level HMIS coverage estimates in both districts compared to the probability samples. However, the extent of these discrepancies varies across the blocks. We would expect consistency among block-level results given their geographic and political proximity within these two districts. Using the combined estimates as our guides, they differ from the respective probability estimates by at most 10%. In contrast, the combined estimates differ from the HMIS estimates by up to 84.2% (FBD (all) in Aurangabad's block Sadar), except for one instance where the difference is 133% due to the administrative coverage being >>100% (distribution IFA during pregnancy in Pach Deuri). SEs for the HMIS estimates are between 1.4 and 32.3 times larger than for the combined estimates (Supplementary Information 2 Tables S1, S9 and S4).

The HMIS data provides two sources of information to calculate the denominator (reported HMIS and Expected Level of Achievement-ELA). Across the 14 blocks of Gopalganj, the two values were very close. In Aurangabad we observe a linear relationship between the two sets of values, with the ELA values being slightly higher than the HMIS recorded values, suggesting the projected data overestimates the numbers reported. Since there was no evidence that one was better than the other, we chose to take the average of both values to define the denominator of the HMIS estimator. This step can be used in future applications.

A general attractiveness of the HMIS results for management purposes is their results apply at the block-level, and we do not wish to lose this property. We are fortunate because this level also corresponds to the strata in the LQAS surveys, and thus, this is the information level provided by the combined estimates. For greater granularity, LQAS is a favourable method to use for AT. In this study, we were also able to increase the sample size by pooling the data from two or three target populations surveyed in parallel within a block.

For peer review only - http://bmjopen.bmj.com/site/about/guidelines.xhtml

Our approach allows us to contrast the two districts of Bihar by studying the block-level (sub-district-level) weighting factors *w*. For example, six indicators across both districts, have weights lower than 0.10, highlighting a large standard error in the HMIS estimates versus those in the probability sample estimates which leads to a wide 95%CI in the HMIS estimates. The contribution of the HMIS estimator to the combined estimator for the indicator measuring visits by any Frontline Worker (FLW) within 24-hours of birth estimator is higher in Gopalganj; the weights range between 0.15 and 0.54, indicating a more balanced contribution between the convenience and the probability sample, meaning that some indicators have similar estimates while others do not. In Aurangabad the only indicator having a contribution weight of the HMIS estimator of over 0.35 in all blocks is three ANC visits. Hence, we are not observing a *systematic* discrepancy across indicators and districts. Some HMIS estimates are similar to the probability survey estimates for specific indicators, other indicators display similarity in only certain sub-districts or districts, while other indicators show consistent discrepancies between HMIS and survey result estimates. Ex post, we can learn from these concordance and discordance patterns how well some indicators are defined and measured across districts.

These Bihar-HMIS AT results differ from the Benin and Madagascar-CHD AT results. In the latter study the administrative data consistently over-estimated prevalence [35]. Such is not the case here as 25.6% of indicators over-estimate and 38% under-estimate coverage. Future research should map the variation in data quality by country and indicators as well as sub-nationally, and understand the reasons for the discrepancies between the HMIS and probability sample. These discrepancies might be due to one or more reasons: poor quality denominators in the HMIS in the state or in specific districts; inaccurate or incomplete recording of the HMIS numerator data in one district or at the block-level; or incomplete transmission of HMIS records from a health centre to the block-level for aggregation[38]. It is also possible that the survey and HMIS indicators are not measuring quite the same health system product. The perception of a respondent to a survey question in their own home and that of the health worker in a health centre may differ. Such indicators may need

For peer review only - http://bmjopen.bmj.com/site/about/guidelines.xhtml

replacement as well. Related to this issue is a question of a more general nature. In situations when two estimates differ substantially, is it advisable to combine conflicting estimates? We think this issue can be answered by the data: as the estimates are different, a compromise suggested by the data is to give appropriate weight to the disparate estimates when combining them – a principle which we demonstrate. Our AT method is grounded on the average squared difference between the HMIS and LQAS estimates of the same quantity (see formula in supplement 1). Two assumptions are essential for applying the formula: (a) the two estimates have the same expectation, i.e. they measure the same indicator, (b) the two estimates are uncorrelated, which is guaranteed by their independent source of data collection. The first assumption requires that the target population, time period and geographical areas are the same in each source of information. The resulting weights assigned to each estimate illustrate the reliance on the quality of the other source of data in terms of variability. The more variable the survey estimate is, the higher the weight for the HMIS contribution to the combined estimate, and vice and versa. As to the prescription for future strengthening of the HMIS and DHIS2, this is a separate issue the solution of which depends on information obtained from principled future research.

Our AT method uses statistical principles to combine data coming from different sources and calculates error for all sources as well as for the resulting combined estimate. This innovative product is valuable. The AT approach is different to WHO's well-intentioned attempt to improve HMIS estimates with Computation Logic; its estimates rely on professional judgements and do not produce error terms or CIs–a limitation noted by the authors[39]. Therefore, the risk associated with their resultant is unknown. The Institute for Health Metrics and Evaluation applies more complex quantitative analyses rather than just human judgments. It produces an estimate of the number of additional children covered or overestimated[40]. However, like Computational Logic, it does not report a measure of its error.

For peer review only - http://bmjopen.bmj.com/site/about/guidelines.xhtml

We are living in an information conscious era with continual demand for, and the ability to produce, more and better data[1 41] obtained from multiple sources. Using accurate information can improve a population's health and reduce waste. We guide the integration of existing data sources to provide a more complete assessment of national health and to reduce the cost to the health system. While we consider numerous data sources, HMIS, which generally do not produce SEs, are touted as having increasing importance for improving the coverage and quality of health services[42 43]. Yet, HMIS are not accurate. Improving and measuring the quality of health information systems is greatly needed[44]. Our examples exemplify a ubiquitous situation, namely, the difficulty with having a good estimator of the denominator. These problems occur in developed and low resource countries[15], leading some policy-makers to call for a restructuring of information systems[44]. The current strategy for improving information involves rolling-out a computerized District Health Information System-2 (DHIS2). Though promising, whether the DHIS2 improves data quality is yet to be determined. Nevertheless, its concern is production of quality numerators. Our study shows that AT detected variation in the quality of the HMIS by indicator and sub-district location, and can be used to strengthen systems like the DHIS2 by identifying indicators and their location where improvement is needed. While it does not tell us the reasons for the errors it shows where they exist and their magnitude. This information can be used to understand and correct the sources of errors, which can improve health programmes and reduce waste.

Although we have discussed in detail the statistical principles of our approach, we should make clear its limitations that the global health community can address. Availability of survey data across multiple districts and sub-districts (Blocks in the case of India) is an issue. Survey data are not always widely available or conducted at a frequency that is needed for timely monitoring of health programmes. Concomitant survey data is essential for using AT to improve routine data. However, one single survey is not necessarily what is needed. Multiple surveys carried out at approximately the same time can be used. What is needed is a central data warehouse where surveys sponsored by government, international and civil society are accessible for use in AT, as are clear descriptions of

For peer review only - http://bmjopen.bmj.com/site/about/guidelines.xhtml

sampling procedures, and data codebooks. We are early in this era of improving data quality. While we have focused in this paper on developing appropriate statistical solutions, other health systems strengthening innovations are also needed. The international community needs to consider survey data as a public good. Whilst their primary use may be for programme strengthening in a small catchment area, when considered together with other databases, survey data can have an enormous impact on improving data quality in the health system as a whole.

These results indicate the need to establish a service working side by side of the DHIS2 to inculcate hybrid estimation using AT as a standard component of district information systems. It should be an independent partner of the DHIS2, under the stewardship of lead international agencies such as UNICEF, the Global Fund to Fight AIDS, Tuberculosis and Malaria, USAID, World Bank and bi-lateral organisations (USAID, DFID). It would work independently and transparently to advance hybrid estimation not only in support of DHIS2, but also, and possibly more importantly, to support local public health practitioners and national policy-makers focus their programs to address priorities.

CONCLUSION

Our statistical innovation, hybrid prevalence estimation using statistical AT, requires concomitant data from a random survey sample[45] conducted at the sub-district-level, and sub-district-level HMIS data. The LQAS survey in Bihar was undertaken for another purpose and so its use in AT was at no additional expense. Other statistical surveys, including the Demographic and Health Survey, are potential candidates. Provided both datasets are available in multiple sub-districts, we can estimate the variability of each indicator between sub-districts, and thus, construct not only a combined estimate but also its 95%CI at both sub-district and district levels. and a 95%CI for the HMIS estimator. With the new visual tool presented here, AT results can be quickly interpreted.

AT is intended to improve the quality and use of the HMIS while reducing waste due to using inaccurate information. This study shows that bringing data from existing household surveys together with HMIS data permits the calculation of more accurate and precise decentralized

For peer review only - http://bmjopen.bmj.com/site/about/guidelines.xhtml

prevalence measures by combining HMIS and probability samples at very little cost. In addition to measuring coverage, it also allows us to evaluate the HMIS in practice and point to corrective measures. Such results lead to better systems for tracking the public's health.

FIGURE CAPTIONS

Figure 1: Distribution and average of w for each indicator and district*

Footnote Figure 1: *Circles with no filling indicate outlier values

Figure 2: HMIS coverage value compared to the 95% CI of the $p_{combined}$ estimate for 10 Indicators*

Footnote Figure 2: *5 categories: (Blue) HMIS coverage is more than 10% below the lower bound. (Light blue) HMIS coverage is less than 10% below the CI lower bound. (Green) HMIS coverage is within the CI. (Light red) HMIS coverage is less than 10% above the CI upper bound, (Red) HMIS coverage is more than 10% above the CI upper bound.

ACKNOWLEDGMENTS

We gratefully acknowledge the essential roles of Hemant Das, Alok Prahdan and Sanjay Biswa for their careful field work supporting the LQAS survey and the HMIS data retrieval. We thank Prof Imelda Bates, Prof Brian Faragher and Nancy Vollmer for their valuable feedback on an earlier version of this manuscript.

AUTHORS' CONTRIBUTIONS

JJV, MP developed the research question; MP, CJ led the development of the mathematical statistics for the annealing technique; JV, BD developed the survey design; JV developed the survey

For peer review only

For peer review only - http://bmjopen.bmj.com/site/about/guidelines.xhtml

methodology; BD managed the survey and data quality; CJ carried out the statistical analyses; JJV obtained the funding and donor support for the research; JJV, MP, CJ interpreted the data; CJ was responsible for data curation; all authors wrote and reviewed the paper.

FUNDING

This research was funded by the Bill and Melinda Gates Foundation Investment ID OPP1142889

DATA SHARING STATEMENT

Household survey data are available upon reasonable request. The recurrent data belong to the Ministry of Health of the State of Bihar and requests for data must be made to them.

COMPETING INTERESTS

The authors have read the statement on competing interests and declare that they have none.

For peer review only

REFERENCES

1. Chan M, Kazatchkine M, Lob-Levyt J, et al. Meeting the demand for results and accountability: a call for action on health data from eight global health agencies. *PLoS Med* 2010;7(1):e1000223. doi: 10.1371/journal.pmed.1000223 [published Online First: 2010/02/04]

2. World Health Organization. Sixtieth World Health Assembly: Resolution and Decisions. Geneva: World Health Organisation, 2007.

3. Mavimbe JC, Braa J, Bjune G. Assessing immunization data quality from routine reports in Mozambique. *BMC Public Health* 2005;5:108. doi: 10.1186/1471-2458-5-108

4. Odhiambo-Otieno GW. Evaluation of existing district health management information systems a case study of the district health systems in Kenya. *Int J Med Inform* 2005;74(9):733-44. doi: 10.1016/j.ijmedinf.2005.05.007

5. Aqil A, Lippeveld T, Hozumi D. PRISM framework: a paradigm shift for designing, strengthening and evaluating routine health information systems. *Health Policy Plan* 2009;24(3):217-28. doi: 10.1093/heapol/czp010

6. Mutemwa RI. HMIS and decision-making in Zambia: re-thinking information solutions for district health management in decentralized health systems. *Health Policy Plan* 2006;21(1):40-52. doi: 10.1093/heapol/czj003

7. Cebul RD, Love TE, Jain AK, et al. Electronic health records and quality of diabetes care. *N Engl J Med* 2011;365(9):825-33. doi: 10.1056/NEJMsa1102519

8. Mtema Z, Changalucha J, Cleaveland S, et al. Mobile Phones As Surveillance Tools: Implementing and Evaluating a Large-Scale Intersectoral Surveillance System for Rabies in Tanzania. *PLoS Med* 2016;13(4):e1002002. doi: 10.1371/journal.pmed.1002002

9. Kawonga M, Blaauw D, Fonn S. Aligning vertical interventions to health systems: a case study of the HIV monitoring and evaluation system in South Africa. *Health research policy and systems* 2012;10(1):2.

For peer review only - http://bmjopen.bmj.com/site/about/guidelines.xhtml

10. Phaswana-Mafuya N, Peltzer K, Ladzani R, et al. Pre- and post-intervention assessment of a PMTCT-programme-strengthening initiative in a rural area of the Eastern Cape, South Africa. *Afr J AIDS Res* 2011;10(1):83-93. doi: 10.2989/16085906.2011.575551

11. Zulu Z, Kunene S, Mkhonta N, et al. Three parallel information systems for malaria elimination in Swaziland, 2010–2015: are the numbers the same? *Public Health Action* 2018;8(1):S13-S17. doi: 10.5588/pha.17.0058

12. Checchi F, Warsame A, Treacy-Wong V, et al. Public health information in crisis-affected populations: a review of methods and their use for advocacy and action. *Lancet* 2017;390(10109):2297-313. doi: 10.1016/S0140-6736(17)30702-X [published Online First: 2017/06/13]

13. Ronveaux O, Rickert D, Hadler S, et al. The immunization data quality audit: verifying the quality and consistency of immunization monitoring systems. *Bull World Health Organ* 2005;83(7):503-10. [published Online First: 2005/09/24]

14. Admon AJ, Bazile J, Makungwa H, et al. Assessing and improving data quality from community health workers: a successful intervention in Neno, Malawi. *Public Health Action* 2013;3(1):56-59. doi: 10.5588/pha.12.0071 [published Online First: 2013/03/21]

15. Woodworth GF, Baird CJ, Garces-Ambrossi G, et al. Inaccuracy of the administrative database: comparative analysis of two databases for the diagnosis and treatment of intracranial aneurysms. *Neurosurgery* 2009;65(2):251-6; discussion 56-7. doi: 10.1227/01.NEU.0000347003.35690.7A [published Online First: 2009/07/25]

16. Hedt BL, Pagano M. Health indicators: eliminating bias from convenience sampling estimators. *Stat Med* 2011;30(5):560-8. doi: 10.1002/sim.3920

17. Jeffery C, Pagano M, Hemingway J, et al. Hybrid prevalence estimation: Method to improve intervention coverage estimations. *Proc Natl Acad Sci U S A* 2018;115(51):13063-68. doi: 10.1073/pnas.1810287115 [published Online First: 2018/12/07]

For peer review only

For peer review only - http://bmjopen.bmj.com/site/about/guidelines.xhtml

18. Bowley AL. Measurement of the precision attained in sampling. *Bulletin of the International Statistical Institute, 22, Supplement to Liv 1, [*1926;22(Supplement):6-62.

19. Neyman J. On the two different aspects of the representative method: the method of stratified and the method of purposive selection. *Journal of the Royal Statistical Society* 1934;97:558-606.

20. Campbell DT, Stanley J. Quasi Experimental Design. Chicago: Rand McNally 1963.

21. Wilbur CL. Vital statistics for the twelfth United States Census. *Quarterly Publications Journal of the American Statistical Association* 1896-97;5:188-200.

22. Observations et exp6riences concernant des d6nombrements repr6sentatifs. Bulletin of the International Statistical Institute; 1895-6; Berne.

23. Maina I, Wanjala P, Soti D, et al. Using health-facility data to assess subnational coverage of maternal and child health indicators, Kenya. *Bull World Health Organ* 2017;95(10):683-94. doi: 10.2471/BLT.17.194399 [published Online First: 2017/11/18]

24. Simmons EM, Singh K, Mpiima J, et al. Assessing coverage of essential maternal and child health interventions using health-facility data in Uganda. *Popul Health Metr* 2020;18(1):26. doi: 10.1186/s12963-020-00236-x [published Online First: 2020/10/11]

25. Care-Family-Health. Integrated Family Health Initiative: Catalysing change for healthy communities. Washington, D.C.: CARE, 2013.

26. NFHS. National Family Health Survey, India. India: Government of India (GOI), 2017.

27. Valadez JJ, Devkota B, Jeffery C, et al. How well do mothers recall their own and their infants' perinatal events? A two-district study using cross-sectional stratified random sampling in Bihar, India. *BMJ Open* 2019;9(12):e031289. doi: 10.1136/bmjopen-2019-031289 [published Online First: 2019/12/21]

28. Robertson SE, Valadez JJ. Global review of health care surveys using lot quality assurance sampling (LQAS), 1984–2004. *Social Science & Medicine* 2006;63:1648-60.

For peer review only - http://bmjopen.bmj.com/site/about/guidelines.xhtml

29. Valadez JJ. Assessing child survival programs in developing countries: testing lot quality assurance sampling. Boston: Harvard University Press 1991.

30. Turner AG, Magnani RJ, Shuaib M. A not quite as quick but much cleaner alternative to the Expanded Programme on Immunization (EPI) Cluster Survey design. *International journal of epidemiology* 1996;25(1):198-203.

31. Davis RH, Valadez JJ. Improving the collection of knowledge, attitude and practice data with community surveys: a comparison of two second-stage sampling methods. *Health Policy Plan* 2014;29(8):1054-60. doi: 10.1093/heapol/czt088

32. Wilbur C. Hadden BD, Hemant Das, Caroline Jeffery, Joseph J. Valadez,. Classical LQAS Compared to CARE LQAS+. Liverpool: Liverpool School of Tropical Medicine, 2017.

33. Valadez JJ, Weiss W, Leburg C, et al. Assessing Community Health Programs: A Trainer's Guide. Using LQAS for Baseline Surveys and Regular Monitoring. St Albans: Teaching-aids At Low Cost 2007.

34. Anoke SC, Mwai P, Jeffery C, et al. Comparing two survey methods of measuring health-related indicators: Lot Quality Assurance Sampling and Demographic Health Surveys. *Trop Med Int Health* 2015;20(12):1756-70. doi: 10.1111/tmi.12605

35. Jeffery C, Pagano M, Hemingway J, et al. Hybrid prevalence estimation: a method to improve intervention coverage estimations. *Proceedings of the National Academy of Sciences* In Press

36. Louis TA. Confidence intervals for a binomial parameter after observing no successes. *The American Statistician* 1981;35(3):154.

37. Rosner B. Fundamentals of Biostatistics. Belmont, CA: Thomson-Brooks/Cole 2006.

38. Chaulagai CN, Moyo CM, Koot J, et al. Design and implementation of a health management information system in Malawi: issues, innovations and results. *Health Policy Plan* 2005;20(6):375-84. doi: 10.1093/heapol/czi044

For peer review only

39. Burton A, Kowalski R, Gacic-Dobo M, et al. A formal representation of the WHO and UNICEF estimates of national immunization coverage: a computational logic approach. *PLoS One* 2012;7(10):e47806. doi: 10.1371/journal.pone.0047806 [published Online First: 2012/11/08]

40. Lim SS, Stein DB, Charrow A, et al. Tracking progress towards universal childhood immunisation and the impact of global initiatives: a systematic analysis of three-dose diphtheria, tetanus, and pertussis immunisation coverage. *Lancet* 2008;372(9655):2031-46. doi: 10.1016/S0140-6736(08)61869-3 [published Online First: 2008/12/17]

41. Boerma T, Eozenou P, Evans D, et al. Monitoring progress towards universal health coverage at country and global levels. *PLoS Med* 2014;11(9):e1001731. doi: 10.1371/journal.pmed.1001731

42. Bruce C. Health management information systems in lower income countries: an analysis of system design, implementation and utilization in Ghana and Nepal. Amsterdam: Royal Tropical Institute, 1997.

43. Lippeveld Theo, Suerborn R, Bodart C. Design and implementation of health information systems. Geneva: World Health Organization 2000.

44. Lippeveld T, Sauerborn R, Sapirie S. Health information systems--making them work. *World Health Forum* 1997;18(2):176-84.

45. Bethlehem JG. The rise of survey sampling. In: Institute ASoER, ed. CBS Discussion Paper: 1572-0314. The Hague: Statistics Netherlands, 2009:28.

For peer review only

For peer review only - http://bmjopen.bmj.com/site/about/guidelines.xhtml

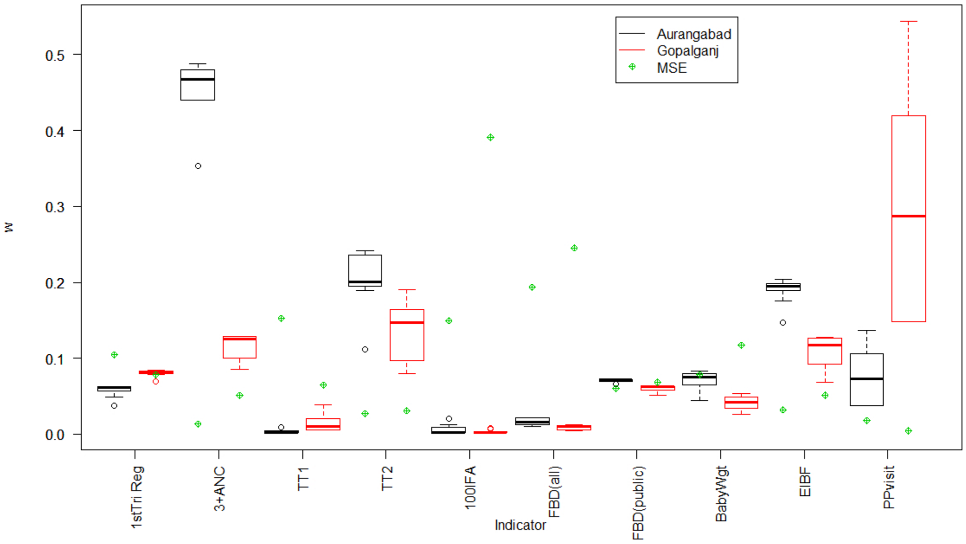

Figure 1: Distribution and average of w for each indicator and district*

407x257mm (72 x 72 DPI)

Block or district	1stTri Reg	3+ANC	TT1	TT2	100IFA	FBD(all)	FBD(public)	BabyWgt	EIBF	PPvisit
Aurangabad Sadar	green	green	blue	green	lightred	blue	blue	blue	blue	green
Barun	green	lightblue	blue	green	red	blue	blue	blue	blue	green
Daudnagar	green	green	blue	lightred	red	blue	green	green	green	green
Deo	green	green	blue	green	red	blue	green	green	green	green
Goh	red	green	blue	lightred	red	lightblue	green	green	green	green
Haspura	green	green	blue	lightred	green	blue	green	green	green	green
Kutumba	red	green	blue	green	red	blue	lightblue	lightblue	green	green
Madanpur	red	green	blue	lightred	red	blue	lightblue	green	lightred	lightred
Nabinagar	red	green	lightblue	green	red	blue	lightblue	green	green	red
Obra	lightred	green	blue	green	red	blue	blue	blue	green	lightred
Rafiganj	red	green	blue	green	red	lightblue	green	lightred	green	lightred
**Aurangabad District Average**	**red**	**green**	**blue**	**lightred**	**red**	**blue**	**blue**	**lightblue**	**lightblue**	**lightred**
Baikunthpur	lightred	lightred	blue	green	red	blue	lightblue	lightblue	green	green
Barauli	red	red	green	lightred	red	blue	lightblue	green	lightblue	green
Bhorey	green	green	lightblue	green	red	blue	green	green	lightblue	lightred
Bijaipur	red	green	lightblue	green	red	blue	blue	blue	blue	lightred
Gopalganj Sadar	red	lightred	green	lightred	red	blue	blue	blue	blue	green
Hathua	lightred	red	blue	lightred	red	blue	green	blue	green	green
Kateya	red	green	green	red	red	blue	lightblue	lightblue	green	green
Kuchaikote	red	red	lightblue	green	red	blue	blue	blue	green	green
Manjha	green	lightblue	lightblue	green	red	blue	blue	blue	lightblue	green
Pach Deuri	green	green	blue	blue	red	blue	lightblue	green	blue	green
Phulwaria	green	green	blue	lightblue	red	blue	green	lightblue	blue	green
Sidhwalia	red	green	blue	green	red	blue	blue	blue	lightblue	green
Thawe	lightred	lightblue	blue	lightred	red	blue	lightblue	blue	blue	green
Uchkagaon	red	green	lightblue	lightred	red	blue	blue	blue	green	lightred
**Gopalganj District Average**	**red**	**lightred**	**blue**	**lightred**	**red**	**blue**	**blue**	**blue**	**blue**	**lightred**

Figure 2: HMIS coverage value compared to the 95% CI of the pcombined estimate for 10 Indicators*

281x148mm (300 x 300 DPI)

Supplementary Information 1

Statistical Annealing Theory

To construct a standard error (SE) for the HMIS coverage estimator, we use the average mean square error (MSE) between the HMIS and LQAS coverage estimators over all the blocks composing one district. The MSE is calculated for the whole district, and can be decomposed in two ways:

$$MSE = \frac{1}{K}\sum_{k=1}^{K}(p_{HMIS\_k} - p_{LQAS\_k})^2 \qquad \text{(first decomposition)}$$
$$= \sigma_{HMIS}^2 + \sigma_{LQAS}^2 \qquad \text{(second decomposition)}$$

where:

* K is the total number of blocks in the district;
* k is the index for the block in the concerned district, and ranges from 1 to K;
* σ_{HMIS} is the SE of the HMIS coverage p_{HMIS};
* σ_{LQAS} is the SE of the LQAS coverage p_{LQAS}.

The first decomposition measures how much the HMIS and LQAS coverage estimators differ across the blocks composing the district. The second decomposition shows the MSE is also the sum of the two variances of HMIS and LQAS estimators, assuming each source of data was collected independently from each other. This decomposition also assumes that both HMIS and LQAS are unbiased, i.e. that the expectation of both estimates is the same). We calculate the MSE using the first decomposition and subtract the LQAS variance to obtain the HMIS variance (and thus σ_{HMIS}).

Combined coverage estimator, SE and confidence interval

We define the combined coverage estimator and its SE as a weighted average of the HMIS and LQAS estimators:

$$p_{combined} = w * p_{HMIS} + (1 - w) * p_{LQAS}$$
$$\sigma_{combined} = \sqrt{w^2 * \sigma_{HMIS}^2 + (1 - w)^2 * \sigma_{LQAS}^2}$$

Where the weighting factor w takes values between 0 and 1:

$$w = \frac{\sigma_{LQAS}^2}{\sigma_{HMIS}^2 + \sigma_{LQAS}^2} = \frac{\sigma_{LQAS}^2}{MSE - \sigma_{LQAS}^2 + \sigma_{LQAS}^2} = \frac{\sigma_{LQAS}^2}{MSE}$$

The 95% CI for the combined coverage estimator is calculated using the standard formula, $p_{combined} \pm 1.96 \times \sigma_{combined}$.

For peer review only

Supplementary Information 2

ANC & Birth preparedness indicators

For the proportion of women who had 3 or more ANC during their pregnancy, the weighting factor ranges from 0.35 to 0.49 in Aurangabad, and from 0.09 to 0.13 in Gopalganj (Table S1). The combined estimate differs from the LQAS estimate by no more than 10%. Standard errors for the HMIS data are 2.8 to 3.4 times larger than those calculated for the combined estimates.

Table S1: Women who had 3 or more ANC visits during their pregnancy.

Block	p_{HMIS}	σ_{HMIS}	p_{lqas}	σ_{lqas}	w	$p_{combined}$	$\sigma_{combined}$	95% CI	$\sigma_{HMIS} / \sigma_{combined}$
Aurangabad Sadar	0.55	0.08	0.55	0.08	0.48	0.55	0.058	(0.44,0.66)	1.44
Barun	0.48	0.09	0.76	0.07	0.35	0.66	0.055	(0.56,0.77)	1.68
Daudnagar	0.71	0.08	0.53	0.08	0.49	0.62	0.058	(0.5,0.73)	1.43
Deo	0.6	0.08	0.58	0.08	0.48	0.59	0.058	(0.48,0.7)	1.45
Goh	0.67	0.09	0.66	0.08	0.44	0.66	0.058	(0.55,0.78)	1.51
Haspura	0.54	0.08	0.61	0.08	0.47	0.58	0.058	(0.46,0.69)	1.46
Kutumba	0.8	0.09	0.66	0.08	0.44	0.72	0.058	(0.61,0.83)	1.51
Madanpur	0.7	0.09	0.66	0.08	0.44	0.67	0.058	(0.56,0.79)	1.51
Nabinagar	0.62	0.08	0.55	0.08	0.48	0.58	0.058	(0.47,0.7)	1.44
Obra	0.73	0.09	0.66	0.08	0.44	0.69	0.058	(0.58,0.8)	1.51
Rafiganj	0.61	0.08	0.61	0.08	0.47	0.61	0.058	(0.49,0.72)	1.46
**Aurangabad District Average**	**0.64**	**0.03**	**0.62**	**0.02**		**0.63**	**0.018**	**(0.6,0.67)**	**1.49**
Baikunthpur	0.72	0.21	0.53	0.08	0.13	0.55	0.076	(0.4,0.7)	2.79
Barauli	0.77	0.21	0.45	0.08	0.13	0.49	0.075	(0.34,0.64)	2.8
Bhorey	0.7	0.21	0.58	0.08	0.13	0.59	0.075	(0.45,0.74)	2.82
Bijaipur	0.8	0.21	0.74	0.07	0.1	0.74	0.068	(0.61,0.88)	3.17
Gopalganj Sadar	0.8	0.21	0.55	0.08	0.13	0.58	0.075	(0.44,0.73)	2.8
Hathua	0.97	0.21	0.47	0.08	0.13	0.54	0.076	(0.39,0.69)	2.79
Kateya	0.8	0.21	0.74	0.07	0.1	0.74	0.068	(0.61,0.88)	3.17
Kuchaikote	0.9	0.21	0.47	0.08	0.13	0.53	0.076	(0.38,0.68)	2.79
Manjha	0.61	0.22	0.79	0.07	0.09	0.77	0.063	(0.65,0.9)	3.42
Pach Deuri	0.5	0.21	0.47	0.08	0.13	0.48	0.076	(0.33,0.62)	2.79
Phulwaria	0.56	0.21	0.58	0.08	0.13	0.58	0.075	(0.43,0.72)	2.82
Sidhwalia	0.72	0.21	0.66	0.08	0.12	0.67	0.072	(0.52,0.81)	2.94
Thawe	0.62	0.22	0.79	0.07	0.09	0.77	0.063	(0.65,0.9)	3.42
Uchkagaon	0.75	0.22	0.76	0.07	0.09	0.76	0.066	(0.63,0.89)	3.28

For peer review only

**Gopalganj District Average**	**0.75**	**0.06**	**0.59**	**0.02**		**0.61**	**0.021**	**(0.57,0.65)**	**2.91**

For the proportion of women who received TT1 during their pregnancy, the weighting factor ranges from 0.002 to 0.009 in Aurangabad, and from 0.006 to 0.038 in Gopalganj (Table S2). The combined estimate differs from the LQAS estimate by no more than 1%. Standard errors for the HMIS data are 5.1 to 20.2 times larger than those calculated for the combined estimates.

Table S2 Women who received TT1 during their pregnancy.

Block	p_{HMIS}	σ_{HMIS}	p_{lqas}	σ_{lqas}	w	$p_{combined}$	$\sigma_{combined}$	95% CI	σ_{HMIS} / $\sigma_{combined}$
Aurangabad Sadar	0.64	0.39	0.95	0.04	0.01	0.94	0.036	(0.87,1)	10.78
Barun	0.61	0.39	0.97	0.03	0	0.97	0.026	(0.92,1)	15.04
Daudnagar	0.61	0.39	1	0.02	0	1	0.019	(0.96,1)	20.19
Deo	0.59	0.39	0.97	0.03	0	0.97	0.026	(0.92,1)	15.04
Goh	0.54	0.39	0.95	0.04	0.01	0.94	0.036	(0.87,1)	10.78
Haspura	0.59	0.39	1	0.02	0	1	0.019	(0.96,1)	20.19
Kutumba	0.62	0.39	1	0.02	0	1	0.019	(0.96,1)	20.19
Madanpur	0.52	0.39	1	0.02	0	1	0.019	(0.96,1)	20.19
Nabinagar	0.87	0.39	0.97	0.03	0	0.97	0.026	(0.92,1)	15.04
Obra	0.54	0.39	1	0.02	0	1	0.019	(0.96,1)	20.19
Rafiganj	0.54	0.39	1	0.02	0	1	0.019	(0.96,1)	20.19
**Aurangabad District Average**	**0.61**	**0.12**	**0.98**	**0.01**		**0.98**	**0.008**	**(0.97,1)**	**15.6**
Baikunthpur	0.65	0.25	1	0.02	0.01	1	0.019	(0.96,1)	13.15
Barauli	0.95	0.25	0.95	0.04	0.02	0.95	0.036	(0.88,1)	7.02
Bhorey	0.88	0.25	1	0.02	0.01	1	0.019	(0.96,1)	13.15
Bijaipur	0.81	0.25	0.95	0.04	0.02	0.94	0.036	(0.87,1)	7.02
Gopalganj Sadar	0.97	0.25	0.95	0.04	0.02	0.95	0.036	(0.88,1)	7.02
Hathua	0.48	0.25	0.97	0.03	0.01	0.97	0.026	(0.92,1)	9.79
Kateya	0.84	0.25	0.89	0.05	0.04	0.89	0.049	(0.8,0.99)	5.11
Kuchaikote	0.93	0.25	1	0.02	0.01	1	0.019	(0.96,1)	13.15
Manjha	0.75	0.25	0.92	0.04	0.03	0.92	0.043	(0.83,1)	5.81
Pach Deuri	0.6	0.25	1	0.02	0.01	1	0.019	(0.96,1)	13.15
Phulwaria	0.63	0.25	1	0.02	0.01	1	0.019	(0.96,1)	13.15
Sidhwalia	0.68	0.25	0.97	0.03	0.01	0.97	0.026	(0.92,1)	9.79
Thawe	0.72	0.25	1	0.02	0.01	1	0.019	(0.96,1)	13.15
Uchkagaon	0.87	0.25	0.95	0.04	0.02	0.95	0.036	(0.88,1)	7.02

For peer review only

**Gopalganj District Average**	**0.78**	**0.07**	**0.97**	**0.01**		**0.97**	**0.008**	**(0.95,0.98)**	**8.82**

For the proportion of women who received TT2 during their pregnancy, the weighting factor ranges from 0.11 to 0.24 in Aurangabad, and from 0.08 to 0.19 in Gopalganj (Table S3). The combined estimate differs from the LQAS estimate by no more than 7%. Standard errors for the HMIS data are 2 to 3.5 times larger than those calculated for the combined estimates.

Table S3 Women who received TT2 during their pregnancy.

Block	p_{HMIS}	σ_{HMIS}	p_{lqas}	σ_{lqas}	w	$p_{combined}$	$\sigma_{combined}$	95% CI	σ_HMIS / σ_combined
Aurangabad Sadar	0.55	0.14	0.63	0.08	0.23	0.61	0.069	(0.48,0.75)	2.1
Barun	0.8	0.15	0.71	0.07	0.2	0.73	0.066	(0.6,0.86)	2.23
Daudnagar	0.82	0.14	0.61	0.08	0.23	0.66	0.069	(0.52,0.79)	2.07
Deo	0.78	0.15	0.74	0.07	0.19	0.75	0.064	(0.62,0.87)	2.3
Goh	0.8	0.14	0.58	0.08	0.24	0.63	0.07	(0.5,0.77)	2.05
Haspura	0.8	0.14	0.58	0.08	0.24	0.63	0.07	(0.49,0.77)	2.05
Kutumba	0.82	0.15	0.71	0.07	0.2	0.73	0.066	(0.6,0.86)	2.23
Madanpur	0.84	0.14	0.55	0.08	0.24	0.62	0.07	(0.48,0.76)	2.03
Nabinagar	0.79	0.15	0.87	0.05	0.11	0.86	0.052	(0.76,0.96)	2.99
Obra	0.85	0.15	0.74	0.07	0.19	0.76	0.064	(0.63,0.88)	2.3
Rafiganj	0.85	0.15	0.71	0.07	0.2	0.74	0.066	(0.61,0.87)	2.23
**Aurangabad District Average**	**0.8**	**0.05**	**0.68**	**0.02**		**0.71**	**0.02**	**(0.67,0.75)**	**2.26**
Baikunthpur	0.86	0.17	0.87	0.05	0.1	0.87	0.052	(0.77,0.97)	3.22
Barauli	1.04	0.17	0.89	0.05	0.08	0.91	0.048	(0.81,1)	3.54
Bhorey	0.85	0.17	0.87	0.05	0.1	0.87	0.052	(0.76,0.97)	3.22
Bijaipur	0.9	0.17	0.84	0.06	0.11	0.85	0.056	(0.74,0.96)	2.98
Gopalganj Sadar	0.98	0.16	0.71	0.07	0.17	0.76	0.067	(0.63,0.89)	2.4
Hathua	0.93	0.16	0.79	0.07	0.14	0.81	0.061	(0.69,0.93)	2.67
Kateya	1.03	0.16	0.71	0.07	0.17	0.77	0.067	(0.64,0.9)	2.4
Kuchaikote	0.87	0.17	0.89	0.05	0.08	0.89	0.048	(0.8,0.99)	3.54
Manjha	0.87	0.16	0.76	0.07	0.15	0.78	0.063	(0.65,0.9)	2.56
Pach Deuri	0.4	0.16	0.74	0.07	0.16	0.68	0.065	(0.55,0.81)	2.47
Phulwaria	0.7	0.17	0.87	0.05	0.1	0.85	0.052	(0.75,0.95)	3.22
Sidhwalia	0.74	0.16	0.76	0.07	0.15	0.76	0.063	(0.63,0.88)	2.56
Thawe	0.84	0.16	0.66	0.08	0.19	0.69	0.069	(0.56,0.83)	2.29
Uchkagaon	0.92	0.16	0.76	0.07	0.15	0.79	0.063	(0.66,0.91)	2.56

For peer review only

**Gopalganj District Average**	**0.87**	**0.05**	**0.81**	**0.02**		**0.82**	**0.016**	**(0.79,0.85)**	**2.96**

For the proportion of women who received at least 100 IFA tablets during their pregnancy, the weighting factor ranges from 0.002 to 0.02 in Aurangabad, and from 0.001 to 0.01 in Gopalganj (Table S4). The combined estimate differs from the LQAS estimate by no more than 1%. Standard errors for the HMIS data are 7 to 32.3 times larger than those calculated for the combined estimates.

Table S4 Women who received at least 100 IFA tablets during their pregnancy.

Block	p_{HMIS}	σ_{HMIS}	p_{lqas}	σ_{lqas}	w	$p_{combined}$	$\sigma_{combined}$	95% CI	σ_HMIS / $\sigma\_combined$
Aurangabad Sadar	0.13	0.39	0.05	0.04	0.01	0.05	0.036	(0,0.12)	10.68
Barun	0.46	0.39	0.03	0.03	0	0.03	0.026	(0,0.08)	14.9
Daudnagar	0.59	0.39	0	0.02	0	0	0.019	(0,0.04)	20.01
Deo	0.45	0.39	0	0.02	0	0	0.019	(0,0.04)	20.01
Goh	0.18	0.39	0	0.02	0	0	0.019	(0,0.04)	20.01
Haspura	0.16	0.38	0.08	0.04	0.01	0.08	0.043	(0,0.17)	8.85
Kutumba	0.34	0.39	0.05	0.04	0.01	0.06	0.036	(0,0.13)	10.68
Madanpur	0.42	0.39	0	0.02	0	0	0.019	(0,0.04)	20.01
Nabinagar	0.6	0.38	0.13	0.05	0.02	0.14	0.054	(0.03,0.25)	7.06
Obra	0.34	0.39	0	0.02	0	0	0.019	(0,0.04)	20.01
Rafiganj	0.51	0.39	0	0.02	0	0	0.019	(0,0.04)	20.01
**Aurangabad District Average**	**0.39**	**0.12**	**0.03**	**0.01**		**0.04**	**0.01**	**(0.02,0.05)**	**12.02**
Baikunthpur	0.52	0.62	0.03	0.03	0	0.03	0.026	(0,0.08)	24.08
Barauli	0.4	0.62	0	0.02	0	0	0.019	(0,0.04)	32.33
Bhorey	0.79	0.62	0.11	0.05	0.01	0.11	0.05	(0.01,0.21)	12.56
Bijaipur	0.29	0.62	0.03	0.03	0	0.03	0.026	(0,0.08)	24.08
Gopalganj Sadar	0.63	0.62	0.05	0.04	0	0.05	0.036	(0,0.13)	17.26
Hathua	0.86	0.62	0.03	0.03	0	0.03	0.026	(0,0.08)	24.08
Kateya	0.8	0.62	0.03	0.03	0	0.03	0.026	(0,0.08)	24.08
Kuchaikote	0.6	0.62	0.03	0.03	0	0.03	0.026	(0,0.08)	24.08
Manjha	0.36	0.62	0.05	0.04	0	0.05	0.036	(0,0.12)	17.26
Pach Deuri	1.47	0.62	0.13	0.05	0.01	0.14	0.055	(0.03,0.25)	11.4
Phulwaria	0.4	0.62	0.05	0.04	0	0.05	0.036	(0,0.12)	17.26
Sidhwalia	0.38	0.62	0.05	0.04	0	0.05	0.036	(0,0.12)	17.26
Thawe	0.59	0.62	0.05	0.04	0	0.05	0.036	(0,0.13)	17.26
Uchkagaon	0.43	0.62	0	0.02	0	0	0.019	(0,0.04)	32.33

For peer review only

**Gopalganj District Average**	**0.59**	**0.18**	**0.04**	**0.01**		**0.04**	**0.009**	**(0.03,0.06)**	**20.2**

Birth history indicators

For the proportion of infants who had institutional delivery, the weighting factor ranges from 0.01 to 0.02 in Aurangabad, and from 0.005 to 0.01 in Gopalganj (Table S5). The combined estimate differs from the LQAS estimate by no more than 1%. Standard errors for the HMIS data are 6.7 to 14.6 times larger than those calculated for the combined estimates.

Table S5 Institutional Deliveries (all institutions)

Block	p_{HMIS}	σ_{HMIS}	p_{lqas}	σ_{lqas}	w	$p_{combined}$	$\sigma_{combined}$	95% CI	σ_HMIS / $\sigma\_combined$
Aurangabad Sadar	0.03	0.44	0.88	0.04	0.01	0.87	0.043	(0.78,0.95)	10.11
Barun	0.28	0.44	0.82	0.05	0.01	0.82	0.05	(0.72,0.92)	8.73
Daudnagar	0.41	0.44	0.81	0.05	0.01	0.8	0.052	(0.7,0.9)	8.41
Deo	0.38	0.43	0.63	0.06	0.02	0.63	0.063	(0.5,0.75)	6.88
Goh	0.38	0.43	0.6	0.06	0.02	0.59	0.064	(0.47,0.72)	6.77
Haspura	0.51	0.44	0.77	0.06	0.02	0.77	0.055	(0.66,0.88)	7.91
Kutumba	0.41	0.44	0.86	0.05	0.01	0.85	0.046	(0.77,0.94)	9.56
Madanpur	0.4	0.44	0.7	0.06	0.02	0.7	0.06	(0.58,0.81)	7.26
Nabinagar	0.25	0.43	0.63	0.06	0.02	0.62	0.063	(0.5,0.75)	6.88
Obra	0.3	0.44	0.84	0.05	0.01	0.84	0.048	(0.74,0.93)	9.1
Rafiganj	0.38	0.43	0.58	0.07	0.02	0.57	0.065	(0.45,0.7)	6.72
**Aurangabad District Average**	**0.34**	**0.13**	**0.73**	**0.02**		**0.72**	**0.018**	**(0.69,0.76)**	**7.59**
Baikunthpur	0.44	0.49	0.79	0.05	0.01	0.79	0.054	(0.68,0.89)	9.17
Barauli	0.47	0.49	0.84	0.05	0.01	0.84	0.048	(0.74,0.93)	10.25
Bhorey	0.48	0.49	0.81	0.05	0.01	0.8	0.052	(0.7,0.91)	9.47
Bijaipur	0.35	0.49	0.82	0.05	0.01	0.82	0.05	(0.72,0.92)	9.83
Gopalganj Sadar	0	0.49	0.84	0.05	0.01	0.83	0.048	(0.74,0.93)	10.25
Hathua	0.55	0.49	0.91	0.04	0.01	0.91	0.037	(0.84,0.98)	13.21
Kateya	0.48	0.49	0.93	0.03	0	0.93	0.034	(0.86,0.99)	14.63
Kuchaikote	0.34	0.49	0.77	0.06	0.01	0.77	0.055	(0.66,0.87)	8.91
Manjha	0.2	0.49	0.81	0.05	0.01	0.8	0.052	(0.7,0.9)	9.47
Pach Deuri	0.46	0.49	0.77	0.06	0.01	0.77	0.055	(0.66,0.88)	8.91
Phulwaria	0.42	0.49	0.84	0.05	0.01	0.84	0.048	(0.74,0.93)	10.25
Sidhwalia	0.31	0.49	0.81	0.05	0.01	0.8	0.052	(0.7,0.9)	9.47
Thawe	0.33	0.49	0.93	0.03	0	0.93	0.034	(0.86,0.99)	14.63
Uchkagaon	0.32	0.49	0.91	0.04	0.01	0.91	0.037	(0.84,0.98)	13.21

**Gopalganj District Average**	**0.37**	**0.14**	**0.84**	**0.01**		**0.83**	**0.014**	**(0.8,0.86)**	**9.96**

For the proportion of infants who had institutional delivery in a public facility, the weighting factor ranges from 0.066 to 0.073 in Aurangabad, and from 0.05 to 0.06 in Gopalganj (Table S6). The combined estimate differs from the LQAS estimate by no more than 4%. Standard errors for the HMIS data are 3.7 to 4.4 times larger than those calculated for the combined estimates.

Table S6 Public Institutional Deliveries

Block	p_{HMIS}	σ_{HMIS}	p_{lqas}	σ_{lqas}	w	$p_{combined}$	$\sigma_{combined}$	95% CI	σ_HMIS / $\sigma\_combined$
Aurangabad Sadar	0.03	0.24	0.65	0.06	0.07	0.61	0.061	(0.49,0.73)	3.88
Barun	0.28	0.24	0.56	0.07	0.07	0.54	0.063	(0.42,0.67)	3.73
Daudnagar	0.41	0.24	0.51	0.07	0.07	0.5	0.064	(0.38,0.63)	3.7
Deo	0.38	0.24	0.44	0.07	0.07	0.43	0.063	(0.31,0.56)	3.73
Goh	0.38	0.24	0.44	0.07	0.07	0.43	0.063	(0.31,0.56)	3.73
Haspura	0.51	0.24	0.61	0.06	0.07	0.61	0.062	(0.48,0.73)	3.8
Kutumba	0.41	0.24	0.61	0.06	0.07	0.6	0.062	(0.48,0.72)	3.8
Madanpur	0.4	0.24	0.58	0.07	0.07	0.57	0.063	(0.44,0.69)	3.75
Nabinagar	0.25	0.24	0.4	0.06	0.07	0.39	0.063	(0.27,0.52)	3.77
Obra	0.3	0.24	0.56	0.07	0.07	0.54	0.063	(0.42,0.67)	3.73
Rafiganj	0.38	0.24	0.44	0.07	0.07	0.43	0.063	(0.31,0.56)	3.73
**Aurangabad District Average**	**0.34**	**0.07**	**0.52**	**0.02**		**0.51**	**0.019**	**(0.47,0.54)**	**3.76**
Baikunthpur	0.44	0.25	0.58	0.07	0.06	0.57	0.063	(0.45,0.69)	4
Barauli	0.47	0.25	0.61	0.06	0.06	0.61	0.062	(0.48,0.73)	4.06
Bhorey	0.48	0.25	0.56	0.07	0.06	0.56	0.064	(0.43,0.68)	3.98
Bijaipur	0.35	0.25	0.65	0.06	0.06	0.63	0.061	(0.51,0.75)	4.14
Gopalganj Sadar	0	0.25	0.56	0.07	0.06	0.53	0.064	(0.4,0.65)	3.98
Hathua	0.55	0.25	0.65	0.06	0.06	0.64	0.061	(0.52,0.76)	4.14
Kateya	0.48	0.25	0.7	0.06	0.05	0.69	0.059	(0.57,0.81)	4.32
Kuchaikote	0.34	0.25	0.58	0.07	0.06	0.56	0.063	(0.44,0.69)	4
Manjha	0.2	0.25	0.53	0.07	0.06	0.51	0.064	(0.38,0.63)	3.96
Pach Deuri	0.46	0.25	0.6	0.06	0.06	0.59	0.063	(0.46,0.71)	4.03
Phulwaria	0.42	0.25	0.49	0.07	0.06	0.49	0.064	(0.36,0.61)	3.95
Sidhwalia	0.31	0.25	0.72	0.06	0.05	0.7	0.058	(0.58,0.81)	4.4
Thawe	0.33	0.25	0.47	0.07	0.06	0.46	0.064	(0.34,0.59)	3.96
Uchkagaon	0.32	0.25	0.58	0.07	0.06	0.56	0.063	(0.44,0.69)	4

For peer review only

**Gopalganj District Average**	**0.37**	**0.07**	**0.59**	**0.02**		**0.58**	**0.018**	**(0.54,0.61)**	**4.04**

FLW support and Neonatal Health indicators

For the proportion of infants who were weighed during delivery, the weighting factor ranges from 0.04 to 0.08 in Aurangabad, and from 0.03 to 0.05 in Gopalganj (Table S7). The combined estimate differs from the LQAS estimate by no more than 3%. Standard errors for the HMIS data are 3.5 to 6.2 times larger than those calculated for the combined estimates.

Table S7 Infants who were weighed during delivery.

Block	p_{HMIS}	σ_{HMIS}	p_{lqas}	σ_{lqas}	w	$p_{combined}$	$\sigma_{combined}$	95% CI	σ_HMIS / $\sigma\_combined$
Aurangabad Sadar	0.1	0.27	0.84	0.06	0.04	0.81	0.058	(0.7,0.92)	4.73
Barun	0.49	0.27	0.76	0.07	0.06	0.75	0.067	(0.62,0.88)	4.06
Daudnagar	0.52	0.27	0.63	0.08	0.08	0.62	0.075	(0.48,0.77)	3.58
Deo	0.46	0.27	0.58	0.08	0.08	0.57	0.077	(0.42,0.72)	3.5
Goh	0.52	0.27	0.55	0.08	0.08	0.55	0.077	(0.4,0.7)	3.47
Haspura	0.61	0.27	0.63	0.08	0.08	0.63	0.075	(0.48,0.78)	3.58
Kutumba	0.53	0.27	0.71	0.07	0.07	0.7	0.071	(0.56,0.84)	3.8
Madanpur	0.57	0.27	0.71	0.07	0.07	0.7	0.071	(0.56,0.84)	3.8
Nabinagar	0.66	0.27	0.66	0.08	0.08	0.66	0.074	(0.51,0.8)	3.64
Obra	0.46	0.27	0.82	0.06	0.05	0.8	0.061	(0.68,0.92)	4.45
Rafiganj	0.6	0.27	0.42	0.08	0.08	0.44	0.077	(0.29,0.59)	3.5
**Aurangabad District Average**	**0.51**	**0.08**	**0.66**	**0.02**		**0.65**	**0.022**	**(0.61,0.69)**	**3.75**
Baikunthpur	0.58	0.34	0.76	0.07	0.04	0.76	0.068	(0.62,0.89)	4.96
Barauli	0.52	0.33	0.61	0.08	0.05	0.6	0.077	(0.45,0.75)	4.31
Bhorey	0.65	0.33	0.63	0.08	0.05	0.63	0.076	(0.48,0.78)	4.37
Bijaipur	0.48	0.33	0.74	0.07	0.04	0.73	0.07	(0.59,0.86)	4.79
Gopalganj Sadar	0.05	0.33	0.74	0.07	0.04	0.71	0.07	(0.57,0.84)	4.79
Hathua	0.54	0.34	0.87	0.05	0.03	0.86	0.054	(0.75,0.97)	6.24
Kateya	0.6	0.34	0.82	0.06	0.03	0.81	0.062	(0.69,0.93)	5.44
Kuchaikote	0.4	0.34	0.79	0.07	0.04	0.77	0.065	(0.65,0.9)	5.17
Manjha	0.29	0.33	0.68	0.08	0.05	0.67	0.074	(0.52,0.81)	4.54
Pach Deuri	0.56	0.33	0.68	0.08	0.05	0.68	0.074	(0.53,0.82)	4.54
Phulwaria	0.5	0.33	0.66	0.08	0.05	0.65	0.075	(0.5,0.8)	4.45
Sidhwalia	0.37	0.34	0.79	0.07	0.04	0.77	0.065	(0.65,0.9)	5.17
Thawe	0.35	0.34	0.82	0.06	0.03	0.8	0.062	(0.68,0.92)	5.44
Uchkagaon	0.44	0.34	0.84	0.06	0.03	0.83	0.058	(0.72,0.94)	5.78

**Gopalganj District Average**	**0.45**	**0.09**	**0.74**	**0.02**		**0.73**	**0.019**	**(0.7,0.77)**	**4.94**

For the proportion of infants who were breast fed within an hour of their birth, the weighting factor ranges from 0.15 to 0.20 in Aurangabad, and from 0.07 to 0.13 in Gopalganj (Table S8). The combined estimate differs from the LQAS estimate by no more than 8%. Standard errors for the HMIS data are 2.2 to 3.8 times larger than those calculated for the combined estimates.

Table S8 Infants who were breast fed within an hour of their birth.

Block	p_{HMIS}	σ_{HMIS}	p_{lqas}	σ_{lqas}	w	$p_{combined}$	$\sigma_{combined}$	95% CI	σ_HMIS / $\sigma\_combined$
Aurangabad Sadar	0.1	0.16	0.5	0.08	0.2	0.42	0.072	(0.28,0.56)	2.22
Barun	0.49	0.17	0.76	0.07	0.15	0.72	0.064	(0.6,0.85)	2.61
Daudnagar	0.52	0.16	0.42	0.08	0.2	0.44	0.072	(0.3,0.58)	2.24
Deo	0.46	0.16	0.5	0.08	0.2	0.49	0.072	(0.35,0.63)	2.22
Goh	0.52	0.16	0.68	0.08	0.18	0.66	0.068	(0.52,0.79)	2.38
Haspura	0.61	0.16	0.61	0.08	0.19	0.61	0.071	(0.47,0.74)	2.27
Kutumba	0.53	0.16	0.63	0.08	0.19	0.61	0.07	(0.47,0.75)	2.3
Madanpur	0.57	0.16	0.37	0.08	0.19	0.41	0.07	(0.27,0.54)	2.3
Nabinagar	0.66	0.16	0.61	0.08	0.19	0.62	0.071	(0.48,0.76)	2.27
Obra	0.46	0.16	0.63	0.08	0.19	0.6	0.07	(0.46,0.74)	2.3
Rafiganj	0.6	0.16	0.58	0.08	0.2	0.58	0.072	(0.44,0.72)	2.24
**Aurangabad District Average**	**0.51**	**0.05**	**0.58**	**0.02**		**0.57**	**0.022**	**(0.52,0.61)**	**2.3**
Baikunthpur	0.58	0.21	0.66	0.08	0.12	0.65	0.072	(0.51,0.79)	2.94
Barauli	0.52	0.21	0.68	0.08	0.11	0.67	0.071	(0.53,0.81)	3
Bhorey	0.65	0.22	0.84	0.06	0.07	0.83	0.057	(0.72,0.94)	3.83
Bijaipur	0.48	0.22	0.76	0.07	0.09	0.74	0.066	(0.61,0.87)	3.28
Gopalganj Sadar	0.05	0.21	0.53	0.08	0.13	0.47	0.076	(0.32,0.61)	2.79
Hathua	0.58	0.21	0.58	0.08	0.13	0.58	0.075	(0.43,0.73)	2.83
Kateya	0.6	0.21	0.63	0.08	0.12	0.63	0.073	(0.48,0.77)	2.89
Kuchaikote	0.4	0.21	0.55	0.08	0.13	0.53	0.075	(0.39,0.68)	2.81
Manjha	0.3	0.21	0.53	0.08	0.13	0.5	0.076	(0.35,0.65)	2.79
Pach Deuri	0.56	0.22	0.82	0.06	0.08	0.8	0.06	(0.68,0.91)	3.6
Phulwaria	0.5	0.22	0.76	0.07	0.09	0.74	0.066	(0.61,0.87)	3.28
Sidhwalia	0.47	0.21	0.71	0.07	0.11	0.69	0.07	(0.55,0.82)	3.08
Thawe	0.35	0.21	0.63	0.08	0.12	0.6	0.073	(0.45,0.74)	2.89
Uchkagaon	0.44	0.21	0.47	0.08	0.13	0.47	0.076	(0.32,0.62)	2.79

**Gopalganj District Average**	**0.46**	**0.06**	**0.64**	**0.02**		**0.62**	**0.02**	**(0.58,0.66)**	**2.95**

For the proportion of newborns who were visited by any FLW within 24hours of home delivery, the weighting factor ranges from 0.04 to 0.14 in Aurangabad, and from 0.15 to 0.54 in Gopalganj (Table S9). The combined estimate differs from the LQAS estimate by no more than 3%. Standard errors for the HMIS data are 1.4 to 5.2 times larger than those calculated for the combined estimates.

Table S9 Home visit by any FLW within 24 hours of delivery (home deliveries only)

Block	p_{HMIS}	σ_{HMIS}	p_{lqas}	σ_{lqas}	w	$p_{combined}$	$\sigma_{combined}$	95% CI	$\sigma\_HMIS / \sigma\_combined$
Aurangabad Sadar	0.02	0.13	0.03	0.03	0.04	0.03	0.025	(0,0.08)	5.18
Barun	0.16	0.12	0.11	0.05	0.14	0.11	0.046	(0.02,0.2)	2.7
Daudnagar	0	0.13	0.03	0.03	0.04	0.03	0.025	(0,0.08)	5.18
Deo	0.01	0.13	0.08	0.04	0.11	0.07	0.041	(0,0.15)	3.08
Goh	0.03	0.13	0.05	0.04	0.07	0.05	0.035	(0,0.12)	3.71
Haspura	0.1	0.12	0.11	0.05	0.14	0.11	0.046	(0.01,0.2)	2.7
Kutumba	0.08	0.13	0.05	0.04	0.07	0.05	0.035	(0,0.12)	3.71
Madanpur	0.18	0.13	0.05	0.04	0.07	0.06	0.035	(0,0.13)	3.71
Nabinagar	0.4	0.13	0.03	0.03	0.04	0.04	0.025	(0,0.09)	5.18
Obra	0.12	0.13	0.03	0.03	0.04	0.03	0.025	(0,0.08)	5.18
Rafiganj	0.23	0.13	0.08	0.04	0.11	0.1	0.041	(0.01,0.18)	3.08
**Aurangabad District Average**	**0.14**	**0.04**	**0.06**	**0.01**		**0.06**	**0.011**	**(0.04,0.08)**	**3.68**
Baikunthpur	0.09	0.05	0.08	0.04	0.42	0.08	0.033	(0.02,0.15)	1.54
Barauli	0.06	0.05	0.08	0.04	0.42	0.07	0.033	(0,0.14)	1.54
Bhorey	0.18	0.06	0.03	0.03	0.15	0.05	0.024	(0,0.1)	2.6
Bijaipur	0.14	0.06	0.03	0.03	0.15	0.04	0.024	(0,0.09)	2.6
Gopalganj Sadar	0.05	0.06	0.05	0.04	0.29	0.05	0.031	(0,0.11)	1.86
Hathua	0.06	0.05	0.08	0.04	0.42	0.07	0.033	(0,0.13)	1.54
Kateya	0.13	0.06	0.05	0.04	0.29	0.07	0.031	(0.01,0.13)	1.86
Kuchaikote	0.07	0.06	0.03	0.03	0.15	0.03	0.024	(0,0.08)	2.6
Manjha	0.09	0.05	0.11	0.05	0.54	0.1	0.034	(0.03,0.16)	1.36
Pach Deuri	0.11	0.06	0.05	0.04	0.29	0.07	0.031	(0.01,0.13)	1.86
Phulwaria	0.08	0.06	0.05	0.04	0.29	0.06	0.031	(0,0.12)	1.86
Sidhwalia	0.16	0.05	0.08	0.04	0.42	0.11	0.033	(0.05,0.18)	1.54
Thawe	0.03	0.06	0.05	0.04	0.29	0.05	0.031	(0,0.11)	1.86
Uchkagaon	0.12	0.06	0.03	0.03	0.15	0.04	0.024	(0,0.09)	2.6

**Gopalganj District Average**	**0.09**	**0.02**	**0.06**	**0.01**		**0.06**	**0.008**	**(0.05,0.08)**	**1.91**

For peer review only
